# 3D organization of regulatory elements for transcriptional regulation in *Arabidopsis*

**DOI:** 10.1186/s13059-023-03018-4

**Published:** 2023-08-07

**Authors:** Li Deng, Qiangwei Zhou, Jie Zhou, Qing Zhang, Zhibo Jia, Guangfeng Zhu, Sheng Cheng, Lulu Cheng, Caijun Yin, Chao Yang, Jinxiong Shen, Junwei Nie, Jian-Kang Zhu, Guoliang Li, Lun Zhao

**Affiliations:** 1 National Key Laboratory of Crop Genetic Improvement, Hubei Hongshan Laboratory, Huazhong Agricultural University, Wuhan, 430070 China; 2 Agricultural Bioinformatics Key Laboratory of Hubei Province and Hubei Engineering Technology Research Center of Agricultural Big Data, 3D Genomics Research Center, Huazhong Agricultural University, Wuhan, 430070 China; 3 Vazyme Biotech Co., Ltd., Nanjing, 210000 China; 4 Institute of Advanced Biotechnology and School of Life Sciences, Southern University of Science and Technology, Shenzhen, 518055 China; 5 Center for Advanced Bioindustry Technologies, Chinese Academy of Agricultural Sciences, Beijing, 100081 China

**Keywords:** Chromatin architecture, Transcriptional regulation, ChIA-PET, *Arabidopsis*

## Abstract

**Background:**

Although spatial organization of compartments and topologically associating domains at large scale is relatively well studied, the spatial organization of regulatory elements at fine scale is poorly understood in plants.

**Results:**

Here we perform high-resolution chromatin interaction analysis using paired-end tag sequencing approach. We map chromatin interactions tethered with RNA polymerase II and associated with heterochromatic, transcriptionally active, and Polycomb-repressive histone modifications in *Arabidopsis*. Analysis of the regulatory repertoire shows that distal active *cis*-regulatory elements are linked to their target genes through long-range chromatin interactions with increased expression of the target genes, while poised *cis*-regulatory elements are linked to their target genes through long-range chromatin interactions with depressed expression of the target genes. Furthermore, we demonstrate that transcription factor MYC2 is critical for chromatin spatial organization, and propose that MYC2 occupancy and MYC2-mediated chromatin interactions coordinately facilitate transcription within the framework of 3D chromatin architecture. Analysis of functionally related gene-defined chromatin connectivity networks reveals that genes implicated in flowering-time control are functionally compartmentalized into separate subdomains via their spatial activity in the leaf or shoot apical meristem, linking active mark- or Polycomb-repressive mark-associated chromatin conformation to coordinated gene expression.

**Conclusion:**

The results reveal that the regulation of gene transcription in *Arabidopsis* is not only by linear juxtaposition, but also by long-range chromatin interactions. Our study uncovers the fine scale genome organization of *Arabidopsis* and the potential roles of such organization in orchestrating transcription and development.

**Supplementary Information:**

The online version contains supplementary material available at 10.1186/s13059-023-03018-4.

## Background

The spatial organization of the genome has a profound impact on transcriptional regulation [1–5]. The 3D genome architecture is widely studied using chromatin interaction mapping approaches. For example, Hi-C and its variants capture genome-wide chromatin interactions [6–13], while ChIA-PET, PLAC-seq, and HiChIP identify target protein-mediated chromatin interactions at nucleotide/binding site resolution through chromatin immunoprecipitation (ChIP) [14–18]. In mammals, chromatin is folded into multi-scale organization, including chromatin loops, architectural stripes, topologically associating domains (TADs)/chromatin contact domains (CCDs), and compartments with various features [19–21]. In contrast, Hi-C studies revealed a different hierarchical organization in plants [22–36], probably due to the absence of the architectural protein CCCTC-binding factor (CTCF), and considerable variations in chromosome number and length, genome size and composition. TAD-like structures had been identified in a broad range of plant species [22, 24, 26–29, 32–36]. In *Arabidopsis*, the TAD-like structures were proposed to correspond to heterochromatic compartments and Polycomb repressive histone modifications-associated interacting domains, suggesting that they should rather be referred to as compacted chromatin domains, as they are not functionally equivalent to TADs in animals [23, 25, 36–38]. In mammals, recent studies revealed that ectopic inter-TAD contacts can occur when CTCF binding at boundaries is abrogated or diminished, and novel loops can lead to misexpression of important genes and severe phenotypical consequences [39–41]. High-throughput methods that aim for systematic identification of chromatin loops, such as ChIA-PET and HiChIP, were recently applied to rice and maize, which confirmed extensive chromatin loops connecting proximal and distal *cis*-regulatory elements (CREs) linking gene expression and key agronomic traits [42–44]. These results provide evidence for the widespread existence of chromatin loops that act over long genomic distances to influence gene expression and phenotypes in plants. However, these fine-scale structures and their effects on transcriptional regulation and development in the model plant *Arabidopsis* remain largely unexplored.

*Trans*-regulatory factors and CREs shape chromatin interaction landscape and coordinate genome transcription [12, 45, 46]. CREs are devoid of nucleosomes, thereby rendering chromatin accessible for transcription factor (TF) binding. Accessible chromatin regions or DNase I hypersensitive sites (DHSs) are the hallmark of regulatory DNA in eukaryotic genomes, and the assay for transposase-accessible chromatin using sequencing (ATAC-seq) and DNase I hypersensitivity mapping have been extensively employed to delineate *cis*-regulatory DNA at nucleotide resolution in *Arabidopsis* [47–51]. CREs are divided into two clusters, proximal regulatory elements (PREs, such as promoters) and distal regulatory elements (DREs, such as enhancers and repressors). However, due to limitations of conventional Hi-C, the long-range target genes of the distal regulatory elements are largely unknown. Structural details about the accurate spatial positioning of these factors for genome transcription activation or repression remain to be explored by higher-resolution approaches in *Arabidopsis*.

The organization of functionally related genes ("operon-like" gene clusters) throughout genome is far from random in eukaryotes, which presents an ideal opportunity to understand the spatial positioning of genes that affects their transcriptional activity as well as to understand the underlying principles of the higher-order genomic architectures regulating specific biological processes [52–54]. Flowering is one of the most crucial events in the plant life cycle. The change from adult to reproductive stage is controlled by floral induction pathways [55] that converge on the upregulation of floral pathway integrators in the shoot meristem, which trigger conversion from a vegetative to an inflorescence meristem identity [56, 57]. Although many key regulators of floral induction and inflorescence meristem identities have been identified in *Arabidopsis*, it is still not clear how they are organized in the context of the promoter/enhancer-centered transcriptional activation network or repressor-mediated transcriptional repression network, and how chromatin conformation relates to their transcriptional activity in *Arabidopsis*.

Recent studies revealed that the genomes of mammals, *Drosophila*, and rice are folded into different spatial subdomains with distinct epigenetic states: transcriptionally active, inactive, and Polycomb-repressive [8, 44, 58–61]. Here, we employed the ChIA-PET approach to investigate the 3D organization of *Arabidopsis* genome with different epigenetic states at peak/binding site resolution. We then dissected the chromatin loops in order to probe how CREs and TFs are organized in the complex milieu of *Arabidopsis* chromosomes and to explore the links between this refined scale of 3D genome architecture and transcriptional regulation. Our comprehensive 3D genome map serves as a valuable resource and provides a deeper understanding of the complex transcription and development regulatory network.

## Results

### Mapping multiscale 3D genome organization in *Arabidopsis*

**Fig. 1:**
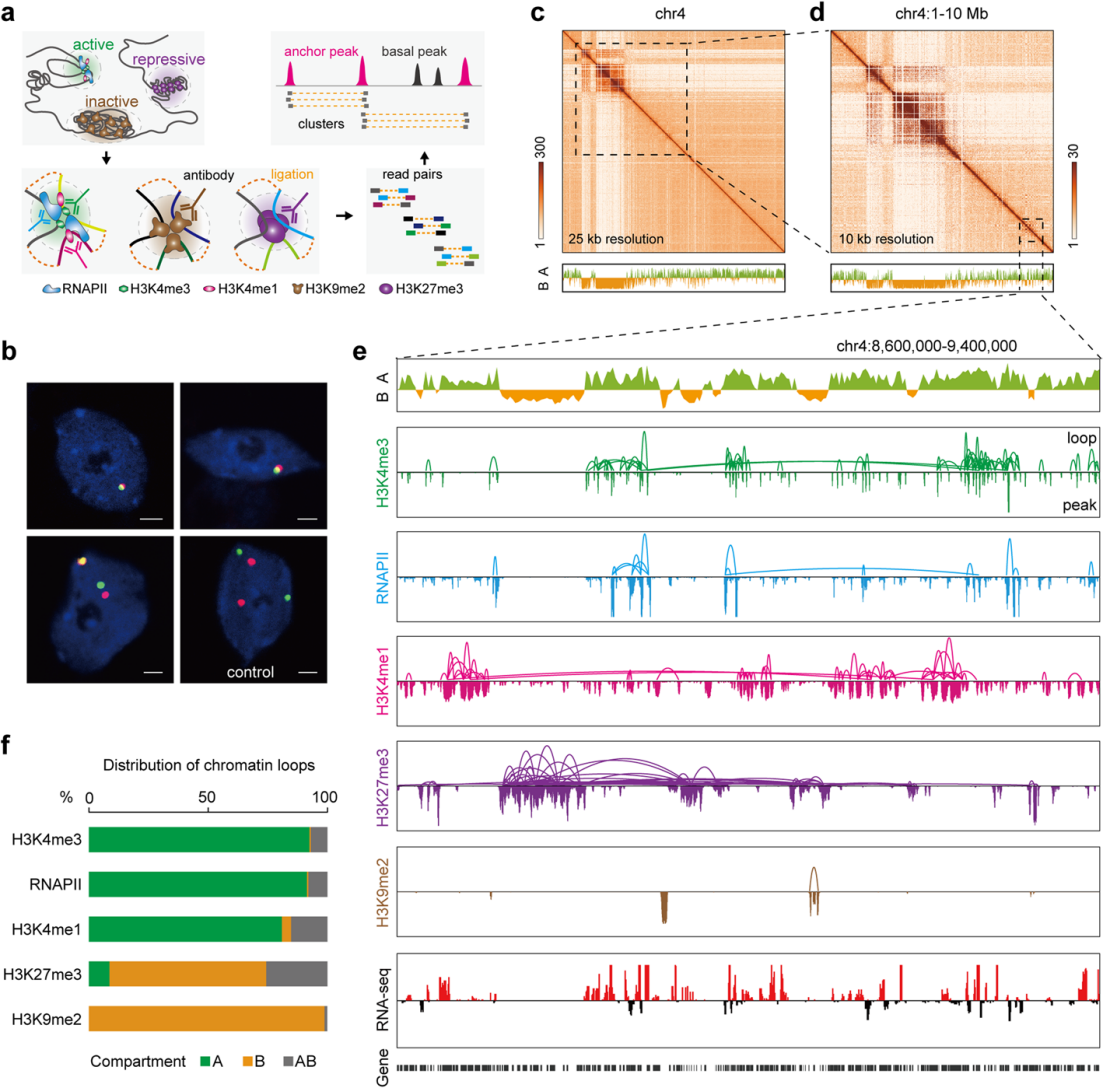
ChIA-PET analysis defines chromatin interactions in *Arabidopsis*. **a** Cross-linked chromatin was fragmented, subjected to ChIP enrichment of three types of representative chromatin marks in *Arabidopsis*, namely transcriptionally active (H3K4me3, RNAPII, and H3K4me1), Polycomb-repressive (H3K27me3), and heterochromatic (H3K9me2), followed by proximity ligation. Deoxyribonucleic acid constructs consisting of two tags from interacting DNA fragments were sequenced. Overlapping regions of inter-ligation paired-end tags (PETs) were used to define chromatin interactions. Anchor peak, a ChIP-seq peak involved in chromatin interaction; basal peak, a ChIP-seq peak not involved in chromatin interaction (see Additional file 2: Table S1 for details). **b** Examples of DNA FISH-analyzed nuclei. Anchor regions are stained red and green, whereas DNA is stained blue. Bar = 2 µm. **c**, **d** Upper: ChIA-PET interaction heatmaps of chromosome 4 at 25 and 10 kb resolution. The graphs are from the combined ChIA-PET data. Lower: A/B compartments. The graphs show the A (green histogram) and B (orange histogram) compartments represented by the first eigenvector from principal component analysis (PCA). **e** Compartments, and chromatin loops and binding peaks of the indicated factor in the box region of panel (**d**). The data tracks show A/B compartments, chromatin loops, profiling of representative histone modifications and RNAPII occupancy, and gene transcription. Of the RNA-seq track, the red and black wiggle plots represent forward and reverse strand RNA-seq reads, respectively. **f** Distribution of the indicated factor-connected interactions among compartments A and B and across A-B

To effectively interrogate high-resolution 3D genome organization in *Arabidopsis* that is largely inaccessible by conventional Hi-C approaches, we captured chromatin loops involved in active genes/transcription, Polycomb-repressive regions, and heterochromatin regions by analyzing H3K4me3, RNAPII and H3K4me1, H3K27me3, and H3K9me2 ChIA-PET data, respectively (Fig. 1a, Additional file 1: Fig. S1). We generated a total of 308 million uniquely mapped paired-end tags from total ChIA-PET libraries (Additional file 2: Table S1). Given the high reproducibility (> 0.95) of biological replicates for each ChIA-PET dataset category (Additional file 1: Fig. S1), we combined the replicate data for further analysis. To confirm the robustness of ChIA-PET and the existence of the chromatin interactions, three paired anchor loci involved in chromatin interactions were validated by DNA fluorescence in situ hybridization (FISH) in *Arabidopsis* seedling nuclei. As expected, DNA FISH confirmed a high frequency of interaction between the two anchor loci than randomly selected regions (Fig. 1b, Additional file 1: Fig. S2a, Additional file 3: Table S2).

In addition to distinguishing chromatin loops with separate representative chromatin states at binding site resolution, it is theoretically possible to reconstruct comprehensive genome architecture by combining all categories of the ChIA-PET data, similar to the Hi-C approach (Fig. 1a, Additional file 1: Fig. S3a). In a side-by-side comparison of pooled ChIA-PET to the high-depth Hi-C data [62] in *Arabidopsis*, ChIA-PET recapitulated all the reported chromatin structures such as compartments and *KNOTs* with comparable data quality (Fig. 1c–e, Additional file 1: Fig. S3a). In addition, ChIA-PET captured extensive RNAPII- and histone modification-associated chromatin loops (Additional file 1: Fig. S3b), which allow us to investigate the features and functions of these chromatin loops in subsequent analysis.

To identify the architectural features of the combined ChIA-PET data, we performed the aggregate chromosome analysis (ACA) [63], whereby contact maps for each chromosome are rescaled, summed, and used to score each feature in an unbiased manner. The ACA map exhibited prominent contacts within and between centromeres, and marginally frequent chromatin interactions both between the two telomeres of each single chromosome and among telomeres of different chromosomes (Additional file 1: Fig. S2b), which suggests that *Arabidopsis* chromosomes adopt a Rabl-like configuration, at least in some developmental stages or types of cells, similar to previous studies [23, 64].

Principal component analysis using the combined ChIA-PET data revealed that the *Arabidopsis* genome was partitioned into two categories of compartments (A and B) (Fig. 1c–e, Additional file 1: Fig. S4), which are Hi-C-defined megabase-scale domains. Compared with B compartments, A compartments exhibited significantly higher peak intensities of active histone marks (H3K4me3, H3K9ac, H3K27ac, and H3K4me1) and RNAPII, as well as significantly higher DNase I signal and transcript levels (Additional file 1: Fig. S5). In contrast, the peak intensities of heterochromatic (H3K9me2) and repressive (H3K27me3) marks were significantly lower in A compartment than those in B compartment (Additional file 1: Fig. S5). Accordingly, A compartments, which covered ~ 57% of the *Arabidopsis* genome, were significantly enriched with active chromatin loops (*n* = 12,230, 93% of H3K4me3; *n* = 7,207, 91% of RNAPII; *n* = 8,464, 81% of H3K4me1) and had fewer Polycomb-repressive (*n* = 431, 9% of H3K27me3) and inactive (*n* = 9, 0.1% of H3K9me2) chromatin loops than B compartments (Fig. 1f).

TADs are sub-megabase scale domains defined by Hi-C and are conserved between different cell types and across mammalian species [20]. TAD-like regions were also detected in rice, wheat, cotton, and several other plant species [65]. With careful scrutiny of our combined ChIA-PET data, TAD-like structures were not prominent in the *Arabidopsis* genome (Additional file 1: Fig. S3a), consistent with previous Hi-C studies [25, 31]. The strongest interactions were exhibited by the blocks of centromeric/pericentromeric heterochromatin, both among sequences within the same centromere/pericentromere and between sequences of different centromeres/pericentromeres (Additional file 1: Fig. S3a). This is in line with previous observations that *Arabidopsis* chromosomes interact extensively through their centromeric/pericentromeric regions, which is visible by light microscopy [25, 66–68]. Loop visualization showed that almost all heterochromatin interaction domains were enriched with H3K9me2-associated loops but lacked transcriptional- and Polycomb-repressive-associated chromatin contacts (Additional file 1: Fig. S4). Active transcriptional- and Polycomb-repressive region-associated chromatin contacts were enriched in the chromosome arms (Fig. 1e), indicating that chromosome arm regions are spatially separated from centromeric/pericentromeric regions and form distinct spatial interacting modules in the nucleus.

Together, our ChIA-PET data not only recapitulated the high-order organization of *Arabidopsis* genome, as well as the spatial separation of the euchromatin and heterochromatin for different epigenetic states at low resolution, but also detected chromatin loops connecting the target protein-bound and histone modification-associated DNA elements at high resolution.

### Characterization of distinct *Arabidopsis* chromatin loops

The resolution gap between 1 and 3D genome maps and the scarcity of known features of chromatin loops significantly limited our understanding of the genome architecture and transcriptional regulation. Taking advantage of the ChIA-PET resolution, we next searched for features underlying chromatin loops to probe the relevance of 3D genome architecture in transcriptional regulation.

**Fig. 2:**
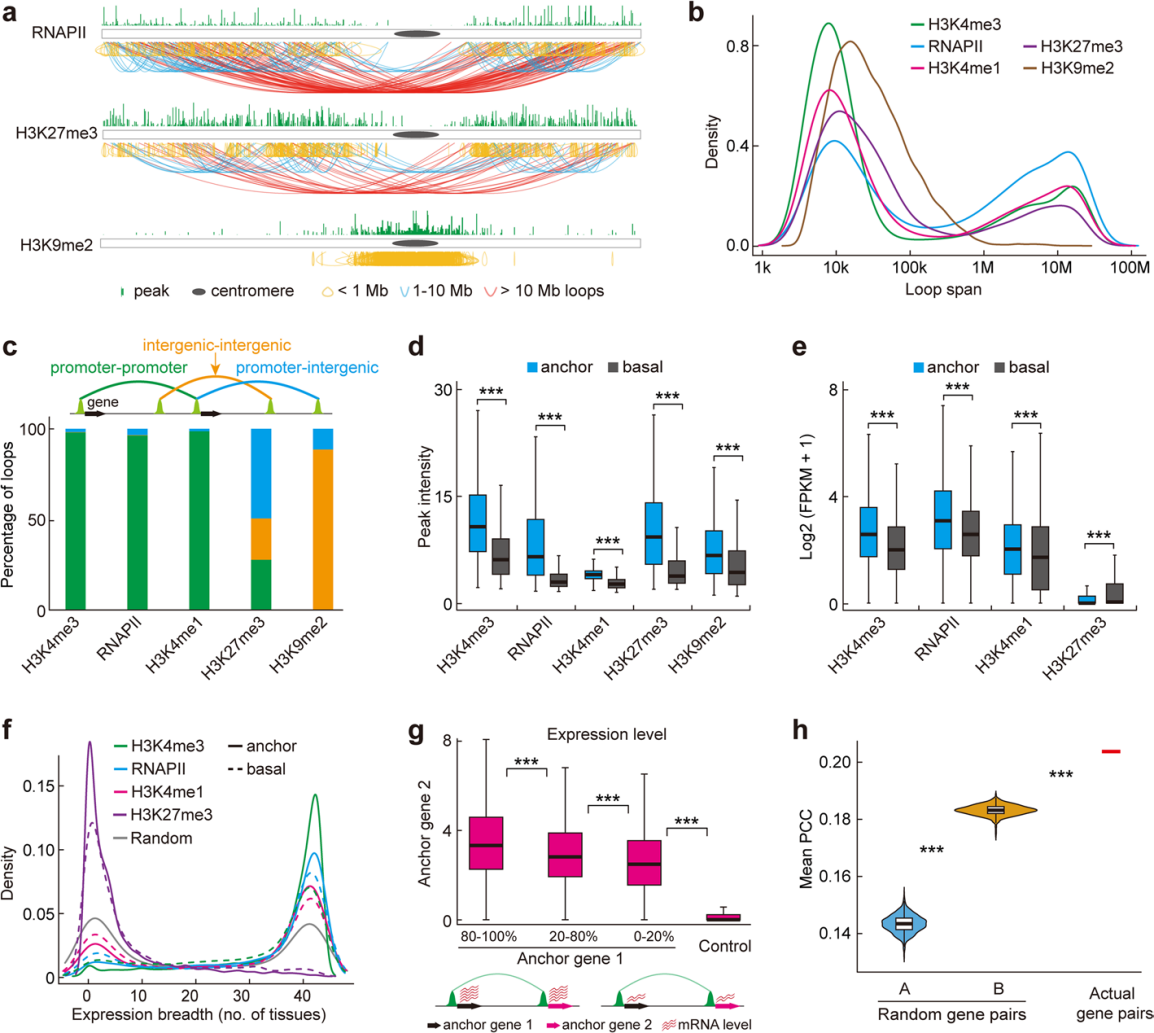
Characterization of ChIA-PET loops. **a** Global views of intrachromosomal interactions tethered by RNAPII, H3K27me3, and H3K9me2 of chromosome 3 in *Arabidopsis*. Green peaks above chromosomes indicate binding sites, and curves under chromosomes indicate chromatin interactions. Yellow, blue, and red curves indicate interaction spans smaller than 1 Mb, between 1 to 10 Mb, and larger than 10 Mb, respectively. **b** Loop span distribution of interactions connected by the indicated factors. **c** Percentages of promoter-promoter, intergenic-intergenic, and promoter-intergenic interactions mediated by the indicated factors. The regions of 1 kb upstream to 0.5 kb downstream of the transcription start site (TSS) of annotated protein-coding genes were defined as promoters. Intergenic regions referred to the regions excluding 1 kb upstream of the TSS to the transcription termination site. **d** Boxplot for intensities of the indicated factor-associated anchor peaks and basal peaks. ***p < 0.001 from Wilcoxon test. **e** Expression levels of the indicated factor-associated anchor genes and basal genes. ***p < 0.001 from Wilcoxon test. **f** Expression breadth (defined as the number of tissues in which a transcript is detected) of the indicated factor-associated anchor genes and basal genes. Random genes served as control. **g** Expression levels of the paired active anchor genes. Anchor genes from H3K27me3 and H3K9me2 loops served as control. We sorted the gene expression levels from high to low and divided them into three categories: 80–100%, 20–80%, and 0–20%. More red wavy lines indicate higher expression. ***p < 0.001 from Wilcoxon test. **h** Co-expression analysis of the active anchor gene pairs. The mean Pearson correlation coefficient (PCC) of anchor gene pairs is much higher than that of both randomly simulated gene pairs A and randomly selected active anchor gene pairs B, which have the same physical distance as anchor gene pairs. ***p < 2.2e-16 from Wilcoxon test

Global chromatin connectivity maps contained ~ 59,000 long-range interactions with various chromatin properties (Additional file 2: Table S1). Of the total 14,063 H3K4me3-binding sites, ~ 68% served as anchors involved in chromatin interactions; and ~ 43% of 10,622 RNAPII, 45% of 14,361 H3K4me1, 55% of 5,790 H3K27me3, and 68% of 4,169 H3K9me2 binding sites also served as anchors (Additional file 1: Fig. S6a). We identified 13,220 H3K4me3-, 7,885 RNAPII-, 10,506 H3K4me1-, and 5,020 H3K27me3-associated intrachromosomal interaction loops (Additional file 2: Table S1). These loops were enriched in the chromosome arms and possessed two loop spans: on one side of the chromosome arm (~ 3–100 kb and ~ 1–10 Mb) and across the centromere (> 10 Mb) (Fig. 2a, b, Additional file 1: Fig. S7, S8, and S10). By contract, H3K9me2 loops were enriched in the centromeric/pericentromeric regions with a medium loop span (~ 39–341 kb; median width, 105 kb) (Fig. 2a, b, Additional file 1: Fig. S9). Of note, the distribution pattern of chromatin loops in *Arabidopsis* differs from those in rice, in which most H3K4me3 intrachromosomal interaction loops were shorter than 1 Mb and formed local chromatin domains (Additional file 1: Fig. S7) whereas broader span loops (1–10 Mb) formed H3K9me2-associated chromatin domains (Additional file 1: Fig. S9) [44]. These observations highlight the pronounced difference in chromosome configurations in the two different model plants.

Three categories of chromatin interactions were identified based on the genome distribution of anchors: promoter-promoter interaction, promoter-intergenic interaction, and intergenic-intergenic interaction (Fig. 2c, Additional file 1: Fig. S6b). Approximately 88% of H3K9me2 loops were intergenic-intergenic interactions, whereas the vast majority (> 96%) of the active chromatin interactions were promoter-promoter interactions, and H3K27me3 was associated with all three interaction categories (Fig. 2c). The peak intensity of all three categories of anchor sites and the expression levels of active anchor genes tended to be higher than those not involved in interactions (basal sites) (Fig. 2d, e). We further adopted the transcriptome data from 43 different tissues (Additional file 4: Table S3) to characterize the expression patterns (including expression breadth and cooperative transcription) of the basal and anchor genes defined in seedlings. We found that the active anchor genes showed wider expression breadth than the active basal genes (Fig. 2f). These results indicated that higher expressed genes tend to be universally expressed and involved in chromatin interactions. However, more Polycomb-repressive anchor genes were in tissue-specific categories compared to Polycomb-repressive basal genes (Fig. 2f). In addition, the paired active anchor genes were simultaneously highly expressed (Fig. 2g, Additional file 1: Fig. S6c–e), and the mean Pearson correlation coefficient of active anchor gene pairs was far beyond that of randomly simulated gene pairs with the same physical distance (Fig. 2h), suggesting that chromatin looping contributes to the co-expression of interacting gene pairs. We also investigated the transcriptional activity of active anchor genes with chromatin loops spanning or not spanning H3K27me3 and/or H3K9me2 peaks. No significant difference in expression levels of active anchor genes with loop regions covered by or not covered by H3K27me3 and/or H3K9me2 peaks were found (Additional file 1: Fig. S6g), suggesting that H3K27me3 and H3K9me2 within loop region had no significant effect on the transcriptional regulation of active anchor genes. In summary, ChIA-PET contact maps at peak/binding site resolution bring previously obscured chromatin loops into sharp focus, and these finer-scale structures may facilitate coordinated transcription.

### Interweaving modular chromosome interacting domains (CIDs) form *Arabidopsis* genome architecture

**Fig. 3:**
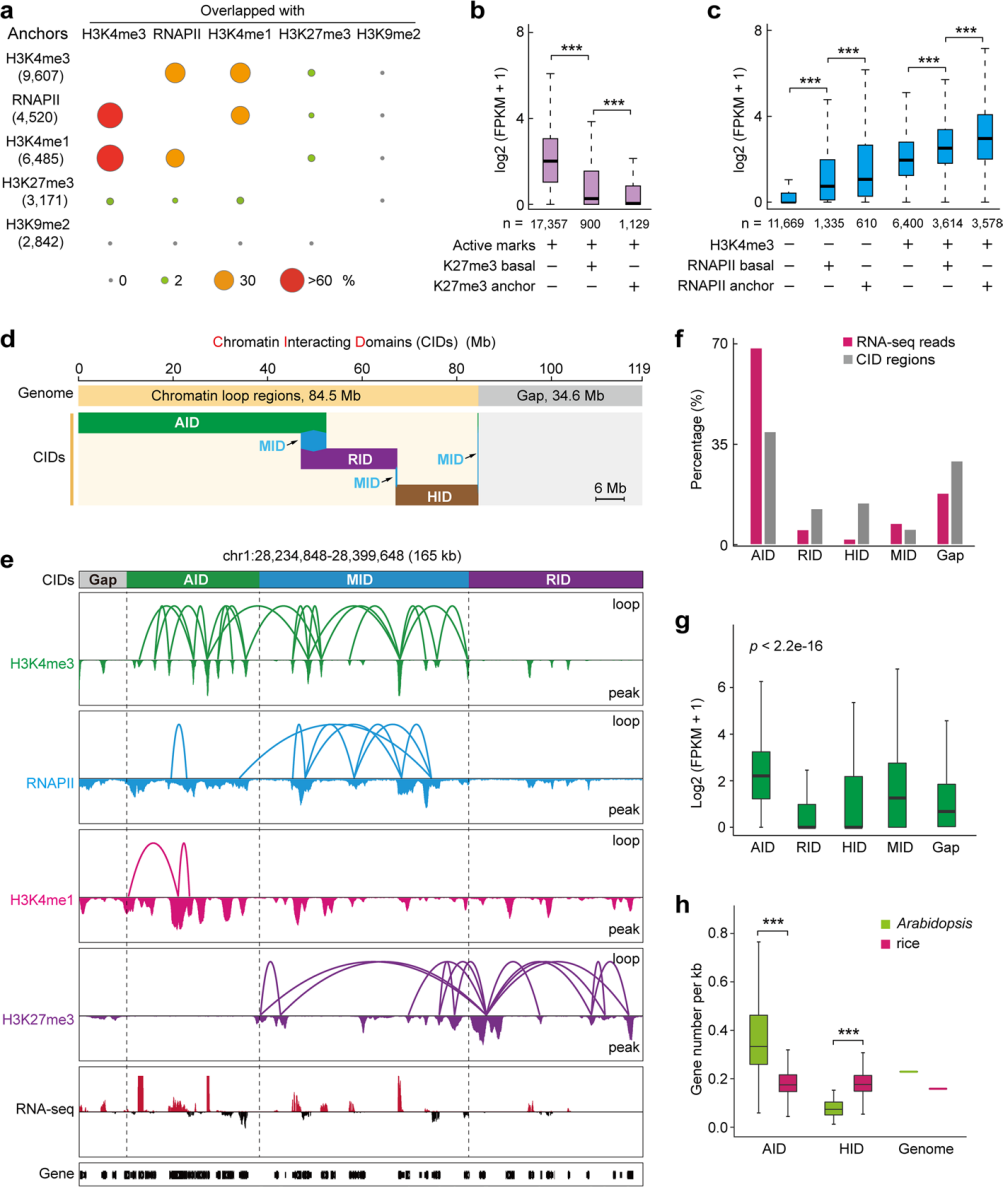
High-order chromatin organization and its transcriptional implications. **a** Overlapping of different types of histone modification and RNAPII determined anchor peaks. The number of anchor peaks for a given histone modification and RNAPII are shown on the left. The percentages of overlap between two indicated factor determined anchor peaks is represented by the area of the circle. **b**, **c** Expression levels among genes with different binding (basal) and interaction (anchor) patterns. ***p < 2.2e-16 from Wilcoxon test. **d** Bar chart of genomic coverage by different chromatin interacting domains (CIDs). CIDs cover 71% of the *Arabidopsis* genome, whereas a minor portion is represented by gap regions with no covering by chromatin interactions (35 Mb, 29%). AID (active interacting domains), 47 Mb, 39%; RID (repressive interacting domains), 15 Mb, 12%; HID (heterochromatin interacting domains), 17 Mb, 14%; and MID (mix interacting domains), 6 Mb, 5%. **e** Browser view of different CIDs showing interval arrangement. The data tracks show CIDs, chromatin loops, profiling of representative histone modifications and RNAPII occupancy, and gene transcription. Within the CID-associated genomic landscape, different CID module categories represent distinct transcriptional activities. **f** Percentage of RNA-seq reads mapped to the indicated CID regions. **g** Boxplot for transcription levels of anchor genes in four CID categories. Genes from gap regions served as control. *p* < 2.2e-16 from Kruskal–Wallis test. **h** Gene density of the AID and HID in *Arabidopsis* and rice. Genome, gene density of genome. ns, no significant difference. ***p < 2.2e-16 from Wilcoxon test

To dissect the associations among different types of epigenetic mark-associated long-range chromatin interactions, we first investigated the relationships between these anchors. H3K4me3-, RNAPII-, and H3K4me1-associated chromatin interactions shared the most anchors (Fig. 3a). Thus, we combined H3K4me3, RNAPII, and H3K4me1 datasets to reconstruct active mark-associated chromosome interacting domains (AIDs). H3K27me3-marked Polycomb targets established physical interactions forming chromatin repressive domains, which had a small fraction of anchor genes overlapped with active mark-associated anchor genes, whereas H3K9me2-marked heterochromatin domains showed almost no overlap with active or Polycomb-repressive mark-associated anchor genes (Fig. 3a), indicating that domains holding distinct epigenetic properties are relatively independent topological units.

We then proceeded to investigate the effects of active- and H3K27me3-associated chromatin interactions on gene transcription. The bivalent genes modified simultaneously with active marks and H3K27me3 displayed lower expression levels than genes with active mark alone (Fig. 3b). In addition, bivalent genes marked with anchor H3K27me3 showed lower expression than those marked with basal H3K27me3 (Fig. 3b). These results suggest that both H3K27me3 enrichment and/or H3K27me3-associated spatial connectivity exert repressive effects on gene transcription. Next, we explored the relationship between H3K4me3- and RNAPII-associated chromatin interactions and their roles in gene transcription. In contrast to genes without the detected active marks, which showed extremely low or no expression, genes occupied by basal RNAPII showed relatively higher expression, which was nevertheless lower than those occupied by anchor RNAPII (Fig. 3c). Furthermore, compared with genes marked with H3K4me3 only, genes occupied by basal and anchor RNAPII along with H3K4me3 showed significantly higher and highest expression levels, respectively (Fig. 3c). Similarly, basal and anchor H3K4me3 was closely tied to incremental effects on gene transcription, which was associated with or without RNAPII occupancy (Additional file 1: Fig. S11), suggesting that RNAPII- and H3K4me3-associated spatial connectivity cooperatively facilitate transcription.

Chromatin interactions further aggregated into higher-order clusters. Based on connectivity and contact frequency, the *Arabidopsis* genome architecture was separated into distinct independent spatial interacting modules. The AID (47 Mb, 39% of the genome), RID (15 Mb, 12%), and HID (17 Mb, 14%) regions refer to active mark-, H3K27me3-, and H3K9me2-associated CIDs, respectively, while the mixed interacting domains (MIDs) refer to heterogeneous chromosome interacting modules (Fig. 3d, e, Additional file 1: Fig. S12a). Distinct CIDs were arranged in intervals in *Arabidopsis* chromosomes with HIDs enriched in centromeric and pericentromeric regions (Additional file 1: Fig. S12b). The greatest transcription abundance (69%) and expressed genes (60%) were enriched in the active mark-related modules (Fig. 3f, Additional file 1: Fig. S12c). We also investigated the transcriptional activity of anchor genes in different CIDs and found that the transcriptional levels of anchor genes in different modules were correlated with the percentages of RNA-seq reads (Fig. 3g), suggesting that the spatially separated modules are independent transcriptional units. Of note, AID regions displayed a higher gene density in *Arabidopsis* than in rice; in contrast, HID regions displayed a lower gene density in *Arabidopsis* compared with rice (Fig. 3h). These observations are in line with the fact that, in *Arabidopsis*, HIDs were exclusively enriched in centromeric and pericentromeric regions, whereas approximately 20% of HIDs were located at euchromatin in rice (Additional file 1: Fig. S9), suggesting a substantial difference in the chromatin organization between these two model plants.

### Definition of CREs with distinct chromatin signatures

**Fig. 4:**
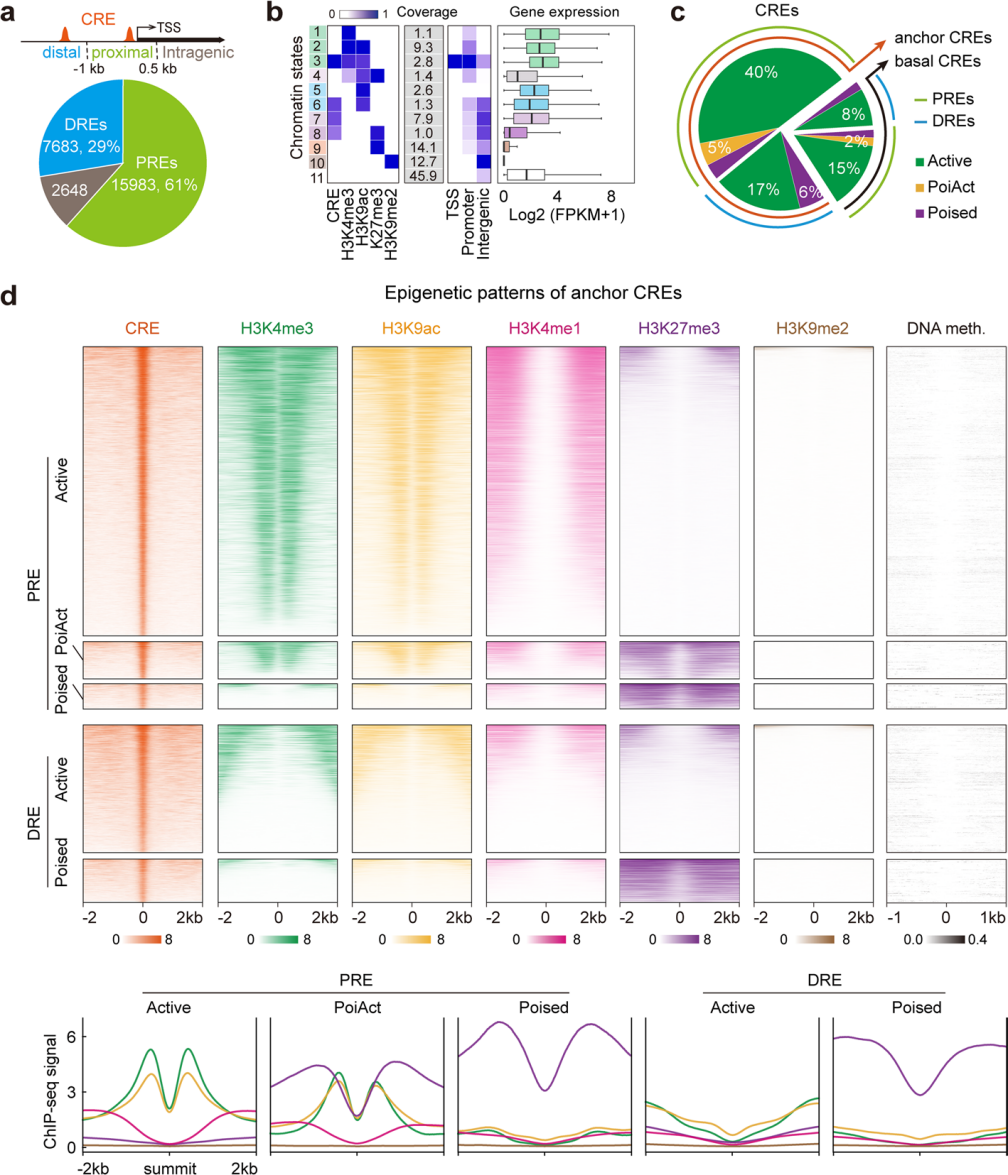
Epigenomic and spatial organization identification of distinct *cis*-regulatory elements (CREs). **a** Distribution of CREs defined by ATAC-seq approach in different genomic regions. PREs, proximal *cis*-regulatory elements; DREs, distal *cis*-regulatory elements. **b** Chromatin states definition, composition (emission probability), genome coverage and genomic annotation enrichments, and expression levels of genes associated with each chromatin state. **c** Percentages of PREs and DREs with and without chromatin interactions that with distinct epigenomic properties. **d** Epigenome heatmaps (upper) and profiles (lower) of different clusters of anchor CREs. Regions shown are ± 2 kb (DNA methylation regions are ± 1 kb) from ATAC-seq peak summits

**Fig. 5:**
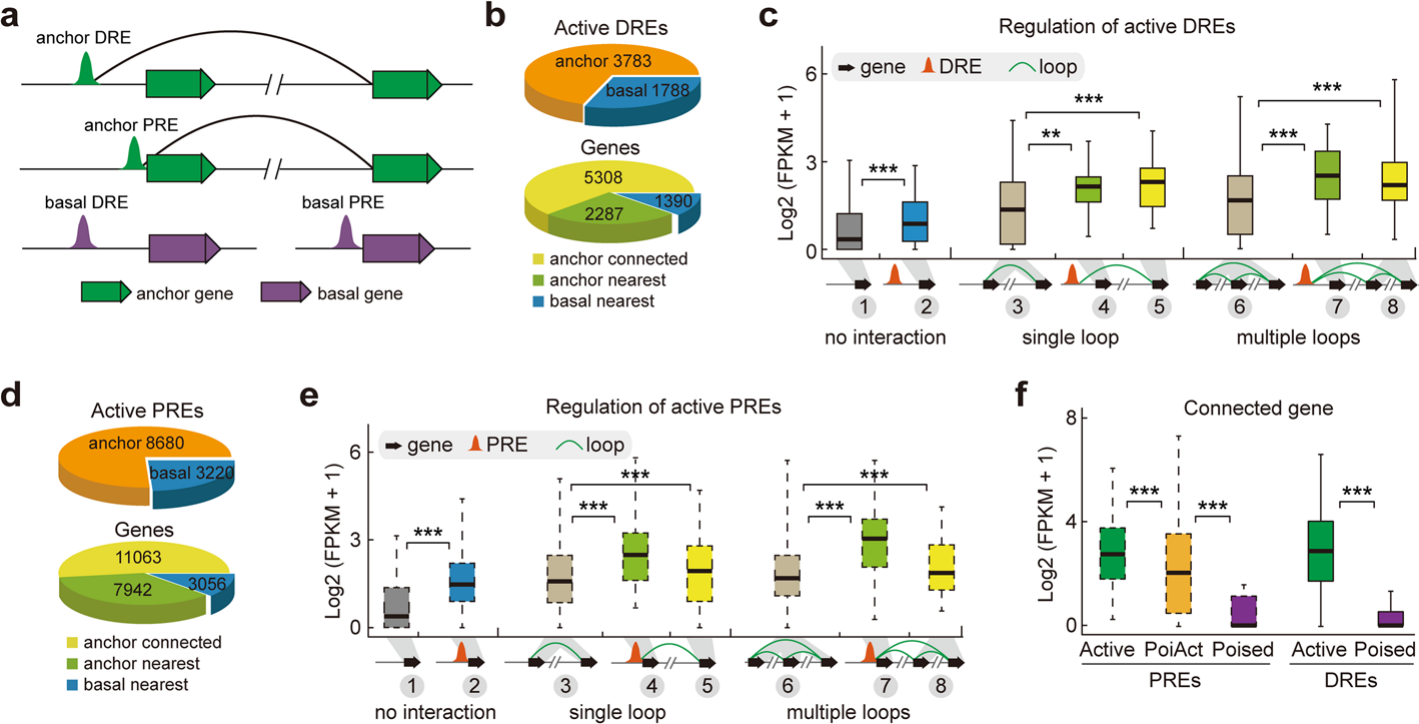
The effects of distinct CREs on transcription. **a** *Cis*-regulatory elements (CREs) models. The anchor DRE model, distal CREs involved in chromatin interactions. The anchor PRE model, proximal CREs involved in chromatin interactions. The basal DRE model, distal CREs not involved in chromatin interactions. The basal PRE model, proximal CREs not involved in chromatin interactions. Solid curve, chromatin loop. **b** Proportion and number of active DREs (upper) and their corresponding genes (lower) involved or not involved in chromatin interactions. **c** Boxplots showing the expression levels of active DRE-associated nearest basal genes, nearest anchor genes, and long-range connecting genes. ***p < 0.001, **p < 0.01 from Wilcoxon test. **d** Proportion and number of active PREs (upper) and their corresponding genes (lower) involved or not involved in chromatin interactions. **e** Boxplots showing the expression levels of active PRE-associated nearest basal genes, nearest anchor genes, and long-range connecting genes. ***p < 0.001 from Wilcoxon test. **f** Boxplots showing the expression levels of PRE- and DRE-associated long-range connecting genes. ***p < 0.001 from Wilcoxon test

To delineate the spatial organization of the regulatory DNA landscape and explore their implications on gene transcription, we generated ATAC-seq data to define the accessible chromatin regions, also known as CREs (Additional file 1: Fig. S13a, b), and performed a comprehensive analysis by combining these accessible chromatin regions, chromatin interactome and transcriptome data. Of the total 26,314 CREs, ~ 61% were located in promoters (1 kb upstream to 0.5 kb downstream of transcription start sites (TSS)) and were defined as proximal CREs or PREs, ~ 29% were located in intergenic regions and were defined as distal CREs or DREs, and the remaining sites were regarded as intragenic CRE (Fig. 4a). Using ChromHMM [59], we established a 11-chromatin state (CS) model, including several combinatorial patterns of CREs and histone modifications (active for CS3 and CS6; repressive/poised for CS8; and CREs alone for CS7) (Fig. 4b). Of the total CREs, most (80%) were active, small proportions were bivalent (7%) and poised (13%) (Fig. 4c), and the two H3K27me3-associated CSs were previously unreported in plants. The same categories of anchor and basal CREs defined based on chromatin interactions showed similar epigenomic properties: active PREs were flanked by active histone marks, whereas active DREs coexisted with none of the examined marks, consistent with the previous concept that active histone marks are not hallmarks of enhancers in plants [47]; bivalent PREs were flanked by active histone marks and H3K27me3 simultaneously and both poised PREs and DREs were flanked with H3K27me3 only (Fig. 4d, 5a, Additional file 1: Fig. S13c). Remarkably, H3K9me2 and DNA methylation were generally excluded from CRE regions (Fig. 4d, Additional file 1: Fig. S13c, S14a).

### Active PREs and DREs are associated with higher transcriptional activity of their nearest and long-range connecting genes

Gene regulatory networks are organized by spatial connectivity between PREs and DREs in mammals [3, 5]. In *Arabidopsis*, previous studies without fine-scale chromatin interaction information confirmed the positive effects of active CREs on their nearest gene expression [47, 48], consistent with our results obtained using the same strategy (Additional file 1: Fig. S14b). Herein, we investigated the transcriptional regulatory effects of active CREs based on the chromatin connectivity maps. Of the total 5,571 active DREs, ~ 70% served as anchors for spatial interactions to regulate 5,308 long-range connecting genes and 2,287 nearest anchor genes, while the rest without detectable interactions were only associated with their nearest basal genes (Fig. 5b). As expected, the expression levels of connecting genes associated with active DREs in both single and multiple loops were significantly higher than those without active DREs (gene category 3 vs. 4, 5; gene category 6 vs. 7, 8) (Fig. 5c). In addition, active DREs were associated with higher transcriptional activity of their nearest genes, irrespective of whether these DREs were involved in chromatin interactions or not (gene category 1 vs. 2, 4, 7) (Fig. 5c). Dozens of DREs were validated as enhancers using a well-established β-glucuronidase reporter assay [47, 48]. Thus, active DREs are putative enhancer candidates and play significant roles in not only their nearest genes but also a large number of interacting genes through long-range chromatin loops.

The transcriptional regulatory pattern of active PREs showed a similar tendency to that of active DREs. For example, the PRE-associated nearest basal genes showed higher expression levels than those without active PREs (gene category 1 vs. 2), and PRE-associated nearest anchor genes (gene category 3 vs. 4; gene category 6 vs. 7) and long-range connecting genes (gene category 3 vs. 5; gene category 6 vs. 8) showed higher expression levels than those anchor genes without active PREs (Fig. 5e). In total, we identified 11,063 long-range connecting genes that were regulated by active PREs (Fig. 5d). These results indicate that enhancer-like PREs are correlated with the increased transcription of both their nearest and beyond their nearest active genes in the spatial connectivity networks.

Next, we investigated the relationship between active PREs and DREs. We found that the nearest anchor genes with both PREs and DREs showed significantly higher expression levels than those with either PREs or DREs alone (Additional file 1: Fig. S14c), implying that PREs and DREs exhibit additive effects in regulating gene transcription in the context of 3D chromatin architecture. However, the additive effect may not exist in CRE-associated basal category, as the nearest basal genes with both PREs and DREs did not display significantly higher expression levels than those with PREs alone in the basal category (Additional file 1: Fig. S14c). Interestingly, PREs produced larger effect in regulating their nearest genes but had smaller effect on long-range genes than DREs, regardless of their participation in chromatin interactions (Additional file 1: Fig. S14c, d). Collectively, we propose that active PREs and DREs serve as enhancer candidates to regulate gene transcription through chromatin interactions independently at some genomic regions while cooperatively at many other regions in the plant cell nucleus.

### Poised CREs act as transcriptional repressors

A distinctive feature of poised CREs is that they were flanked by H3K27me3 only, while PoiAct CREs (bivalent state) were flanked simultaneously by both active marks and H3K27me3 (Fig. 4d, Additional file 1: Fig. S13c). Next, we explored the transcriptional effects of these two H3K27me3-associated CREs on their connecting genes. The expression levels of poised PRE-connecting genes were significantly lower than those linked to active PREs; similarly, poised DRE-connecting genes exhibited lower expression levels compared with those related to active DREs (Fig. 5f). In addition, PoiAct PRE-connecting genes exhibited significantly higher expression levels compared with those associated with poised PREs (Fig. 5f). These results suggest that H3K27me3-associated CREs serve as putative repressor candidates in mediating transcriptional gene repression through long-range chromatin loops.

### Chromatin interactions and TF occupancy coordinately facilitate transcription in the context of 3D chromatin architecture

**Fig. 6:**
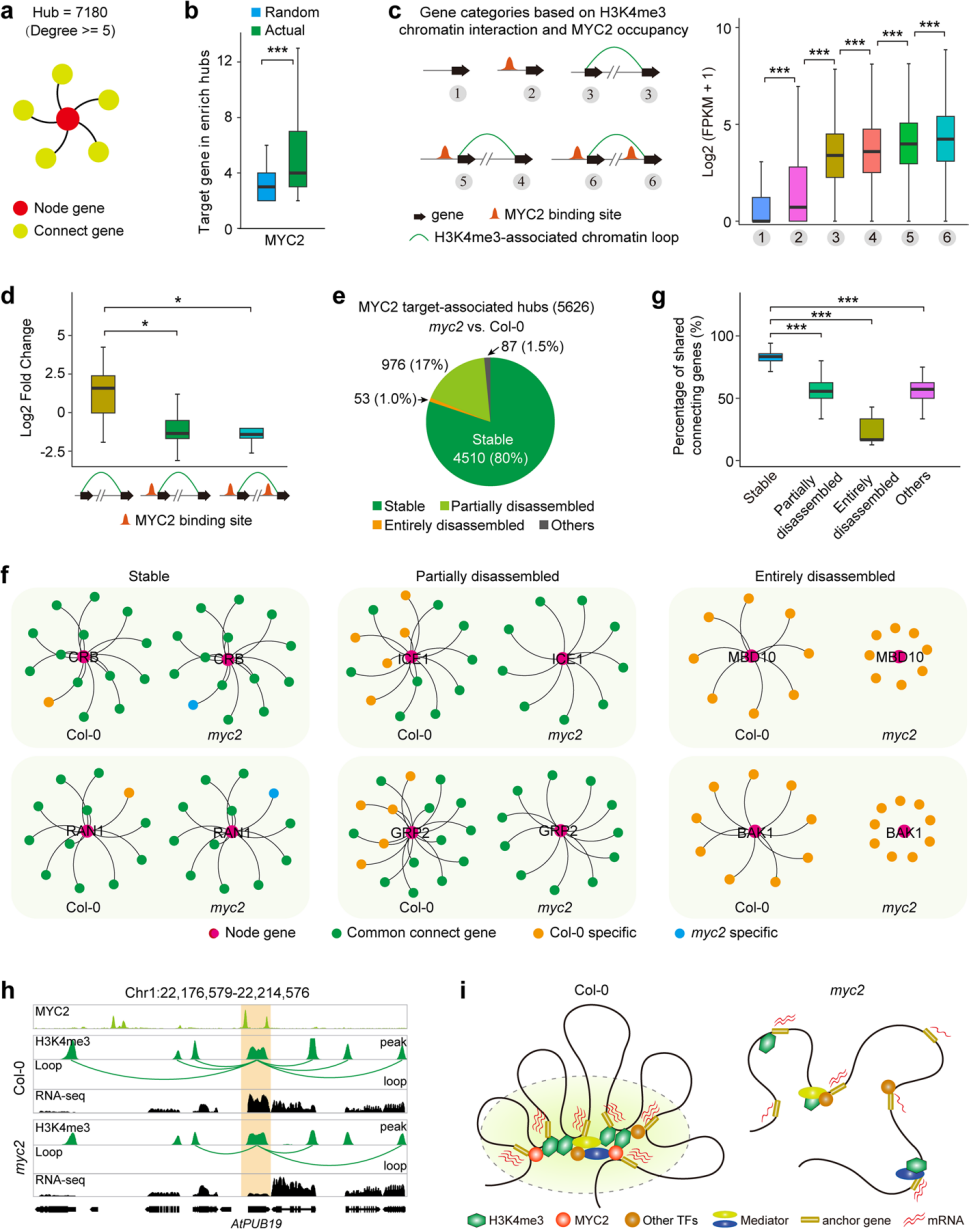
TF-associated chromatin topology and its transcriptional function. **a** Illustration of chromatin hub model of chromatin interactions using the contact frequency (degree >  = 5). The total number of H3K4me3-associated chromatin hubs is 7180. **b** Distribution of the number of MYC2 target genes and randomly selected genes in chromatin hubs. ***p < 2.2e-16 from Wilcoxon test. **c** Expression levels of genes in different categories based on active mark-associated chromatin interaction models and MYC2 occupancy. ***p < 0.001 from Wilcoxon test. **d** Log2 fold change in expression of looping genes associated with lost loops in *myc2* mutant compared to the wild-type. Fold-changes represent the expression changes of each gene in *myc2* mutant relative to the wild-type. The lost loops were divided into three categories based on the MYC2 occupancy model. *p < 0.05 from Wilcoxon test. **e** Changes of MYC2 target-associated chromatin hubs in *myc2* mutant compared to wild-type. These hubs were classified into four categories: stable, entirely and partially disassembled, and others. The number and proportion of hubs included in each category were given. **f** Representative examples of stable, partially, and entirely disassembled hubs in *myc2* and wild-type. **g** Percentages of shared connecting genes in the indicated hub categories between *myc2* and wild-type. ***p < 0.001 from Wilcoxon test. **h** Browser screenshot showing the chromatin connectivity and gene transcription within AtPUB19-centric hub. Light orange box indicates reduction of chromatin connectivity within the AtPUB19-centric hub and downregulation of *AtPUB19* gene expression. Each MYC2 binding site, chromatin interaction, H3K4me3 peak, and RNA-seq track represent the normalized loops and read coverage in *myc2* and wild-type. **i** A proposed model for the impacts of MYC2 in 3D genome organization and gene transcription. In the MYC2 dominated chromatin hub, MYC2 coupled with other TFs and mediators to create microenvironment that facilitates gene transcription in the wild-type. As MYC2 activity is abolished, the MYC2-centric hub is disassembled and the gene expression decreases. More red wavy lines indicate higher expression

Chromatin conformation is thought to shape TF activity, for example, by looping TF-bound CREs to promoters of distally located target genes for transcription regulation in mammalian cells [46, 69]. To investigate the relationship between TF-associated chromatin topology and gene transcription in *Arabidopsis*, we performed comprehensive analysis of five published TF (ABI5, ATHB5, MYC2, CCA1, and SOC1) ChIP-seq data (Additional file 11: Table S10), chromatin interactome and transcriptome data. The TF binding sites were preferentially enriched in promoter and intergenic regions (Additional file 1: Fig. S15a). Compared with basal TF binding sites, anchor TF binding sites displayed higher and lower distributions in active and bivalent regions, respectively (Additional file 1: Fig. S15b, c). As previously reported [3, 70], regulatory element-associated chromatin loops form chromatin hubs; herein, the node gene was defined as the anchor site from which multiple interactions were emanating, and the divergent interaction sites were considered as the connecting genes (Fig. 6a). To explore the spatial organization of target genes of each specific TF, we first investigated the distribution of TF target genes in chromatin hubs (Additional file 5: Table S4). We found that 1,208 chromatin hubs were shared by all five TF target genes, and only a small fraction of hubs were specific to each TF target genes (Additional file 1: Fig. S15d–f). The number of TF target genes were greater than the number of randomly selected genes in chromatin hubs (Fig. 6b, Additional file 1: Fig. S15g), indicating that TF target genes are significantly enriched in specific hubs (See Methods). Together, these results suggest that TF target genes tend to tether together in the context of 3D chromatin architecture.

We further investigated the relationships among long-range chromatin interactions, TF occupancy, and transcription. Taking MYC2 as an example, the genes were divided into six categories based on H3K4me3-associated chromatin interactions and MYC2 occupancy model (Fig. 6c). The expression levels of anchor genes with MYC2 co-occupancy on one anchor (gene category 5) were significantly lower than those on both anchors (gene category 6), but higher than those without MYC2 binding (gene categories 3 and 4) (Fig. 6c). In addition, for basal genes not involved in chromatin interaction, MYC2 bound genes (gene category 2) displayed higher expression levels than MYC2 unbound genes (gene category 1) (Fig. 6c). A similar tendency was also observed in other TF and chromatin topology combination models (Additional file 1: Fig. S15h–k), indicating that chromatin loops and TFs coordinately facilitate transcription in the context of 3D chromatin architecture. To further evaluate whether TF-associated long-range chromatin interactions are related to additive effects on gene expression, we explored the transcription levels of genes with two TF ChIP-seq data combined interaction maps. We found that anchor genes involved in TF-bound CRE associated multiple loops (gene category IN) displayed higher expression levels than those involved in single loops (gene category 4) (Additional file 1: Fig. S15l). These results suggest that TF-associated chromatin topology influence gene transcription in an additive manner in nuclear microenvironments.

### Roles of transcription factor MYC2 in regulating 3D genome architecture and gene transcription

To explore whether transcription factors, such as MYC2, may have a regulatory role in 3D genome organization and gene transcription, we generated chromatin interactome (H3K4me3 ChIA-PET) and transcriptome (RNA-seq) data using an improved method in *myc2* mutant [71] and wild-type plants (Additional file 6: Table S5). Considering the high reproducibility (> 0.95) of biological replicates for each ChIA-PET and RNA-seq dataset category (Additional file 1: Fig. S16, Additional file 6: Table S5), we combined the replicate data for downstream analysis.

We identified 32,312 H3K4me3-associated chromatin loops in *myc2*. Compared to the wild-type, approximately 19% H3K4me3-associated chromatin loops were lost in *myc2*, suggesting that MYC2 is partially responsible for the formation of chromatin loops. To investigate the impact of chromatin interaction and TF occupancy on gene expression, we evaluated the expression changes (*myc2* vs. Col-0) of looping genes within those lost loops in *myc2*. Based on the MYC2 occupancy model, we categorized those lost loops into three categories (Fig. 6d). As expected, the Log2 fold change of the looping genes with MYC2 binding were lower than those without MYC2 binding (Fig. 6d). These results suggest that MYC2 occupancy and chromatin interaction play a crucial role in positively regulating gene transcription.

We next explored whether chromatin hubs mediated by MYC2 will be disassembled if the MYC2 activity is abolished. Of the total 7,971 H3K4me3-associated chromatin hubs detected in *myc2* and wild-type, approximately 71% (5,626) contained MYC2 ChIP-seq target genes (Additional file 1: Fig. S17a). These MYC2 target-associated hubs were classified into four categories based on the change ratios of connecting genes between *myc2* and wild-type: stable, entirely and partially disassembled, and others (Fig. 6e, f). Of the 5,626 MYC2 target-associated hubs, 80% were stable hubs where most of their connecting genes were identical between wild-type and *myc2* (e.g., CRB and RAN1 hubs); 17% were partially disassembled hubs in *myc2* compared to wild-type (e.g., ICE1 and GRP2 hubs); 1% were entirely disassembled hubs where most of their connecting genes were disassembled in *myc2* (e.g., MBD10 and BAK1 hubs) (Fig. 6e–g). The remaining hubs (1.5%, others) either were formed newly or exhibited significant changes in size in *myc2* (Fig. 6e). These results suggest that MYC2 may not impact all chromatin interactions associated with H3K4me3 but plays a crucial role in determining the spatial architecture of the *Arabidopsis* genome, particularly in relation to chromatin hubs associated with MYC2 targets (Fig. 6e, Additional file 1: Fig. S17b).

Furthermore, we classified the genes within the disassembled hubs into two categories: genes that retained chromatin interaction in *myc2* compared to the wild-type, referred to as aggregated genes, and genes that lost chromatin interaction in *myc2*, referred to as separated genes. Notably, the expression of the separated genes was significantly inhibited in *myc2* compared to the aggregated genes (Additional file 1: Fig. S17c). This observation suggests a positive correlation between chromatin interactions and gene transcription within the high-order 3D genome organization, specifically in chromatin hubs. For instance, AtPUB19, a U-Box E3 ubiquitin ligase involved in regulating abscisic acid and drought responses [72], was bound by MYC2 and served as the node gene within the AtPUB19-centric hub in wild-type (Fig. 6h). The absence of MYC2 led to reduction of chromatin connectivity within the AtPUB19-associated hub and also downregulation of *AtPUB19* gene expression (Fig. 6h). These results suggest that TFs may create a conducive microenvironment for gene transcription by reorganizing the spatial genome organization, particularly in chromatin hubs.

Collectively, our results demonstrate the regulatory roles of TFs, exemplified by MYC2, in governing 3D genome organization and gene transcription (Fig. 6i). We propose that MYC2, in conjunction with other TFs and mediators, orchestrates the formation of specialized microenvironments called chromatin hubs, which actively facilitate gene transcription in the wild-type. Notably, MYC2 is necessary for a subset of these chromatin hubs. Therefore, the removal of MYC2 disrupts this microenvironment, leading to the disassembly of MYC-centric hubs and a subsequent downregulation of gene transcription.

### Chromatin loops are associated with single-nucleotide polymorphism (SNP)-affected phenotype variation

**Fig. 7:**
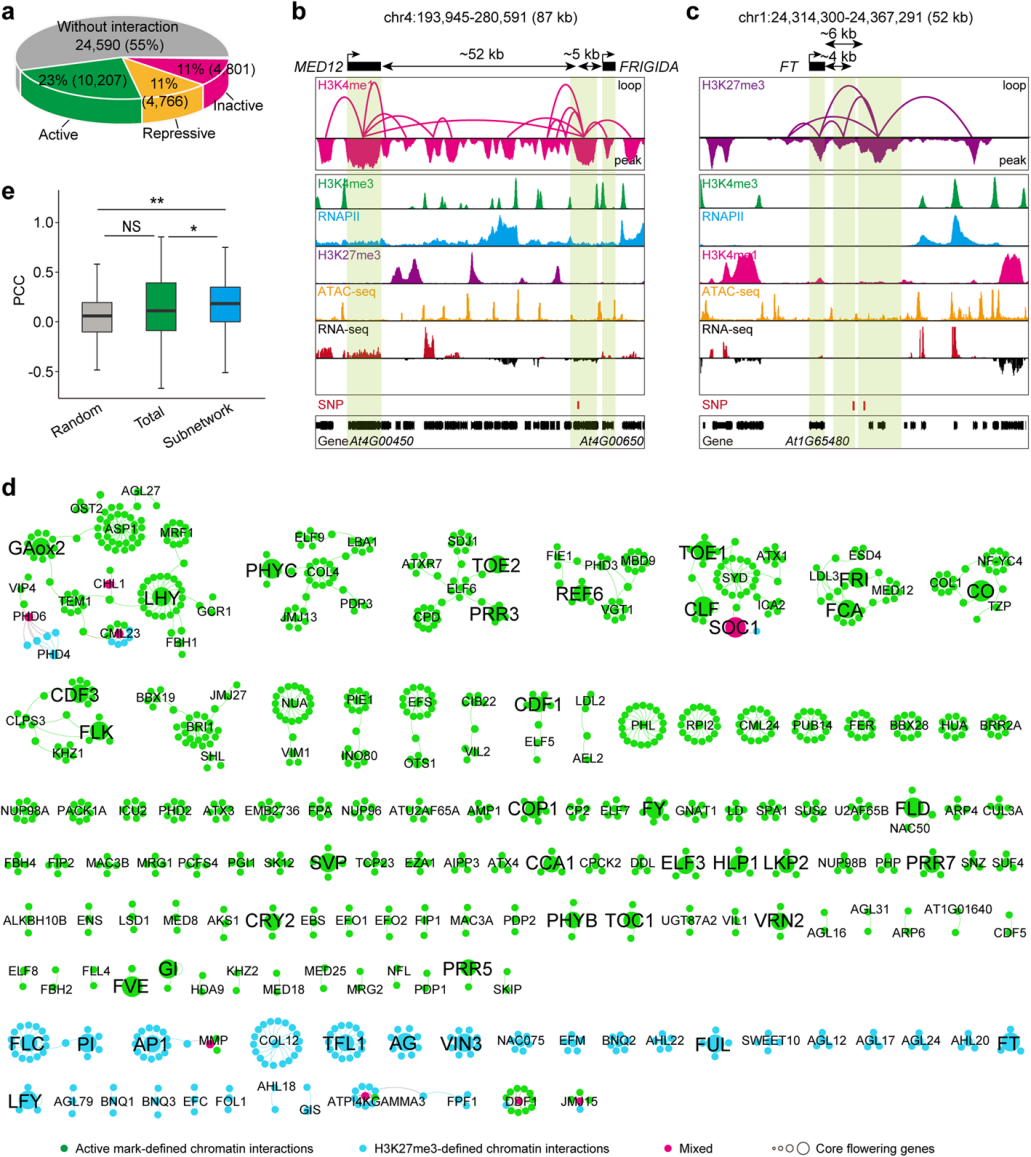
Chromatin loops connect SNPs to the genes causing phenotypic changes and spatially distinct chromatin connectivity networks defined by flowering-time control genes. **a** Pie chart for the proportion of trait-associated significant SNPs involved in active-, repressive- and inactive-mark associated chromatin loops and not involved in chromatin interactions. **b**, **c** Mapping browser screenshot showing the H3K4me1-associated chromatin interactions, ChIP-seq, ATAC-seq, and RNA-seq profiles, and SNPs at *MED12* and *FRIGIDA* loci (**b**) and H3K27me3-associated chromatin interactions, ChIP-seq, ATAC-seq, and RNA-seq profiles, and SNPs at *FT* locus (**c**). Light green boxes indicate the SNP-associated anchor peaks and flowering-time control gene loci. **d** Flowering-time control gene-associated chromatin connectivity network during floral induction. The connectivity was built through one hop of all interactions mediated from 190 genes. Large circles represent the key flowering-time control genes. Small circles with gene symbol represent the node genes implicated in flowering-time control. Small circles without gene symbol represent the connecting genes. Colors represent their 3D network specificities. **e** Co-expression analysis of genes in floral transition-associated subnetworks. The PCC of subnetworks were calculated. Random, randomly selected gene pairs with the same physical distance. Total, all representative chromatin mark-associated anchor gene pairs. **p < 0.01, *p < 0.05 from Wilcoxon test. NS, no significant difference

In mammals, many SNPs have been identified to influence target genes that are hundreds of kilobases away via chromatin interactions [16]. Here, in *Arabidopsis*, we found approximately 23%, 11%, and 11% of phenotype-associated significant SNPs fall in active, repressive, and inactive mark-associated chromatin loops, respectively (Fig. 7a). To test whether long-range chromatin interactions spatially link distal elements in which SNPs are located to target genes and thereby contribute to phenotypic variations in *Arabidopsis*, we selected the complex trait of flowering time control. We found 14 distal elements overlapping flowering time-associated SNPs were involved in chromatin interactions and connected the targeted genes implicated in flowering-time control, suggesting that these SNPs may be associated with flowering time control via loop formation (Additional file 7: Table S6). For instance, an SNP regulating *Arabidopsis* flowering time was mapped to intron of At4G00630, 52 kb downstream of *MED12*, a transcription regulator [73], and 5 kb upstream of *FRIGIDA*, a regulator of vegetative phase change [74]. We have now detected abundant interactions between the SNP-located distal elements and *MED12*, and *FRIGIDA* loci, respectively (Fig. 7b). Moreover, two SNPs mapped to the intergenic region, which were found to be located in a distal element interacting with the florigen gene *FLOWERING LOCUS T* (*FT*) frequently (Fig. 7c). These results indicate that SNPs potentially affect phenotypic variation by influencing the target genes through chromatin interactions. However, this functional chromatin interaction model requires further functional validations, such as transgenic or genome editing assays.

### Flowering-time control genes are functionally compartmentalized into spatially distinct chromatin connectivity networks

To further investigate the properties and potential function of flowering-time control gene-associated spatial chromatin connectivity networks, we explored spatial chromatin connectivity of these flowering-time control genes through active (H3K4me3/RNAPII/H3K4me1) and H3K27me3 interaction maps. Based on the isolation of loss-of-function mutations or analysis of transgenic plants, approximately 241 genes known to govern flowering time were collected (Additional file 8: Table S7) [55]. Interestingly, 190 of these flowering-time control genes were integrated to the chromatin connectivity maps, with 153 genes involved in active connectivity networks, 29 genes implicated in H3K27me3-associated connectivity networks, and 8 genes in active/H3K27me3-associated (mixed) chromatin networks (Fig. 7d, Additional file 8: Table S7, Additional file 9: Table S8); only 51 genes were not detected to be involved in chromatin interactions in our data (Additional file 8: Table S7). The genes involved in floral induction, such as those related to photoperiod, circadian clock, metabolic status, and gibberellin pathway, and regulators of *FLC*, were largely in active chromatin networks (Fig. 7d). Shoot apical meristem responses genes [55], such as *LEAFY* (*LFY*), *TERMINAL FLOWER 1* (*TFL1*), *AGAMOUS-LIKE 24* (*AGL24*), *APETALA1* (*AP1*), *FRUITFULL* (*FUL*), inflorescence identity genes [56], such as *AGAMOUS* (*AG*) and PISTILLATA (*PI*), florigen gene *FT*, and *FLOWERING LOCUS C* (*FLC*), were implicated in H3K27me3-associated connectivity networks (Fig. 7d). These results implied that the chromatin conformation coordinates the initiation of the floral induction programs and repression of inflorescence meristem identification programs to properly control vegetative to reproductive phase switches. Moreover, the spatially distinct chromatin connectivity networks are approximately in line with the spatio-temporal activity of flowering time genes in the leaf and the shoot apical meristem. In addition, the anchor genes linked by flowering-time control gene-networks tended to be more co-expressed than all the interacting genes and randomly simulated gene pairs (Fig. 7e), suggesting that functionally related genes tend to tether together for coordinated transcription.

When the network analysis was extended from one to two hops of connectivity, the flowering-time control genes were found to be connected within five major subnetworks, except for a small fraction of sporadic interactions (Additional file 1: Fig. S18, Additional file 10: Table S9). Among them, circadian clock-associated genes had extensive connectivity (Additional file 1: Fig. S18), in line with the notion that the circadian rhythm governs a large variety of physiological and metabolic functions and the associated genes are more likely to be involved in chromatin interactions [44, 70]. Florigen gene locus has limited connected edges in one H3K27me3-associated spatial hub (Fig. 7c, d), implying the repressive role of local microenvironments in *FT* transcription regulation. Gene ontology (GO) analysis of these interacting genes suggests that their functions are significantly enriched in the regulation of reproductive processes (Additional file 1: Fig. S19), consistent with the known biological processes involved in flowering time control.

## Discussion

In this study, we provided an overall glimpse of *Arabidopsis* 3D genome organization at multiple scales and investigated the roles of genome organization in linking gene transcription. Functional chromatin loops are increasingly being recognized to play an important role in regulating many important genes [75, 76]. Although potential regulatory elements have been identified using high throughput approaches in *Arabidopsis* [47–50], it is still challenging to connect distal DNA elements to their target genes, since a large percentage of distal elements may regulate genes beyond the closest ones [3, 5]. Thus, methods to identify such long-range relationships and recognize their targets is urgently needed. Unlike chromosome conformation capture (3C)/Hi-C derivative approaches, ChIA-PET is a robust method for capturing specific target protein-associated chromatin interactions through proximity ligation and chromatin immunoprecipitation (ChIP). ChIA-PET can capture long-range chromatin loops between *cis*-regulatory elements, such as promoters and enhancers, at nucleotide/binding-site resolution. Since TADs or TAD-like domains were not prominent in *Arabidopsis*, ChIA-PET is more suitable than Hi-C to uncover previously obscured chromatin loops and thereby to establish functional connections between chromatin loops and gene regulation in *Arabidopsis*. Using the ChIA-PET method, we identified extensive chromatin loops connecting proximal and distal regulatory elements, revealing that long-range associations and chromatin loops are widespread finer-scale structures in *Arabidopsis*. Recently, by analyzing mutants defective in DNA methylation, ATP-dependent chromatin remodeling complexes, or histone H3K27 methylation or demethylation, researchers uncovered that DNA methylation-linked chromatin accessibility, chromatin remodeling complexes, and reversible histone modifications influence genome architecture in *Arabidopsis* [38, 77, 78]. Given the important roles of the epigenetic marks in chromatin packing, we mapped 3D chromatin interactomes of the DNA elements with different histone modifications and investigated the regulatory relationships of spatial subdomains with distinct epigenetic states in orchestrating transcription in *Arabidopsis*.

Emerging models propose that phase separation, a phenomenon of macromolecule compartmentalization without subcellular membranes, is critically involved in nuclear organization and function. Heterochromatin regions form liquid-like foci mediated by H3K9me2/3 and its readers [79, 80], acetylated chromatin form multivalent interactions with multi-bromodomain proteins and facilitate the phase separation of chromatin [81], and the transcriptional machinery at genomic loci are formed by phase-separated proteins that contain intrinsically disordered domains, such as RNAPII, TFs, and coactivators [82, 83] in mammal cells. The phase separation-based regulation is proposed to promote spatial proximity between regulatory elements with similar states of activity and/or biophysical properties. Thus, our chromatin interactome profiling of three types of representative chromatin marks imply that active, Polycomb-repressive, and inactive regions of the genome containing three different sets of multivalent proteins may be able to interact with members of their own class, forming three different phases that preclude inter contacts. A mechanistic link between transcription, regulatory element-bound TFs and chromosome folding in the dynamic assembly of phase-separated condensates is fascinating, and requires extensive functional validations in future research.

As demonstrated here, long-range chromatin interactions derived from ChIA-PET data could provide the connectivity of GWAS-identified significant SNPs to their target genes, and thus offer possible mechanistic explanations to the function of trait-associated elements in plants. Further investigation of chromatin connectivity networks revealed that the pivotal genes in flowering-time control were functionally compartmentalized into separate subnuclear domains according to their spatial activity in the leaf or the shoot apical meristem. Most genes involved in floral induction pathways were embedded in active mark-associated “transcription factories” segregated from the surrounding chromosome environment. Shoot apical meristem response genes were associated with Polycomb-repressive chromosomal domains, indicating that H3K27me3-centered chromatin connectivity configures 3D genomic structures as transcription-repressing foci, and maintains meristem identity genes in a repressive/poised chromatin state. In the switch to inflorescence meristem identity, we assumed that meristem identity genes may be released from the H3K27me3-associated repression domains and sequestered into active transcription factories, where transcriptional activation may be facilitated. Since cell/stage-specific chromatin organization may reflect transcriptional regulatory circuitry [3, 56], meristem identity gene-associated 3D genome organization may contribute to developmental phase switches by presumably orchestrating gene expression changes in meristems to repress previous developmental programs and establish new ones.

## Conclusions

Taken together, this study not only provided the landscape of 3D genome architecture in different epigenetic states at peak/binding site resolution in *Arabidopsis*, but also yielded new insights into the links between 3D genome organization and transcriptional regulation, particularly how proximal and distal CREs are associated with the transcription of connecting genes near or distant, how TF-associated chromatin interactions and TF occupancy coordinately facilitate gene transcription within the context of 3D chromatin architecture, and how flowering-time gene-defined distinct chromatin connectivity networks are coordinated in their expression regulation.

## Methods

### Plant material preparation

*Arabidopsis* accession Columbia (Col-0) and *myc2* were grown at 22 °C under a 16 h light/8 h dark photoperiod. The aerial part of two-week-old seedlings were dual cross-linked with 1.5 mM ethylene glycol bis (succinimidylsuccinate) (Thermo Fisher Scientific, 21,565) for 30 min and 1% formaldehyde (SIGMA, F8775) for 10 min, and then quenched with 0.2 M glycine for 5 min at room temperature. Harvested samples were stored at -80 °C for ChIA-PET assay. For ChIP-seq assay, samples were crosslinked with 1% formaldehyde for 10 min, quenched with 0.2 M glycine at room temperature, and then stored at -80 °C until use. Samples were immediately frozen in liquid nitrogen and stored at -80 °C for RNA-seq assay.

### ChIA-PET library preparation

The ChIP procedure was performed according to the enhanced ChIP method [84]. In brief, about 5 g of sample was used for each ChIA-PET assay. Tissues were ground to fine powder in liquid nitrogen and lysed in 10 ml of Buffer S (50 mM HEPES–KOH (pH 7.5), 150 mM NaCl, 1 mM EDTA, 1% Triton X-100, 0.1% sodium deoxycholate, 1% SDS) for 20 min at 4 °C. The homogenate was mixed with 20 ml of Buffer F (50 mM HEPES–KOH (pH 7.5), 150 mM NaCl, 1 mM EDTA, 1% Triton X-100, 0.1% sodium deoxycholate), and the chromatin was fragmented into 1–3 kb by sonication using a Bioruptor (Diagenode). The lysates were centrifuged at 20,000 × *g* for 5 min at 4 °C and 20 ml of Buffer F were added to the supernatant for ChIP. ChIP was performed using antibodies against the following: H3K4me1 (ABclonal, A2355), H3K4me3 (ABclonal, A2357), H3K9me2 (Abcam, ab1220), H3K27me3 (ABclonal, A2363), and RNAPII (BioLegend, 920,102). The antibodies were validated by the manufacturers and our team [84]. Antibody (50–80 μg) was added to Dynabeads® protein G beads (Life Technologies, 10003D) and incubated for 6 h on a rotator at 4 °C. Beads were washed with phosphate-buffered saline with Tween® (PBST) twice and incubated with sonicated chromatin overnight at 4 °C with rotation. Subsequently, beads were washed twice for 5 min each at 4 °C in 5 ml low-salt buffer (50 mM HEPES–KOH, 150 mM NaCl, 1 mM EDTA, 1% Triton X-100, 0.1% sodium deoxycholate, 0.1% SDS), followed by two washes in 5 ml high-salt buffer (low salt ChIP buffer in which 150 mM NaCl was replaced with 350 mM NaCl), one wash in 5 ml LiCl wash buffer (10 mM Tris–HCl pH 8.0, 250 mM LiCl, 0.5% NP-40, 1 mM EDTA, 0.1% sodium deoxycholate), and one wash in TE buffer (10 mM Tris–HCl, pH 8.0, and 1 mM EDTA). The following procedures were performed as previous reported [14, 44]. Briefly, ChIP DNA on beads was used for end-repair and A-tailing using T4 DNA polymerase (Promega, cat. no. M421F) and Klenow enzyme (NEB, cat. no. M0212L). Proximity ligation of ChIP DNA was performed using biotinylated bridge-linker, Forward strand: 5′-[5Phos]CGCGATATC/iBIOdT/TATCTGACT-3′, Reverse strand: 5′-[5Phos]GTCAGATAAGATATCGCGT-3′). Proximity ligation DNA was reverse cross-linked, and the ChIA-PET libraries were prepared using Tn5 transposase (VAHTS; cat. no. TD501). DNA fragments containing the bridge-linker at ligation junctions were captured by Dynabeads™ M-280 Streptavidin (Invitrogen, 11205D), and used as templates for PCR amplification. The libraries were then subjected to size-selection and sequenced (2 × 150 bp) using Illumina Hiseq X-Ten.

### ATAC-seq library preparation

The ATAC-seq libraries were constructed according to the previous study with some modifications [85]. Approximately 1 g of tissue was used for each ATAC-seq assay. The samples were chopped with a razor blade in 1 × PBS buffer to obtain the intact nuclei. After filtration through Miracloth, Triton X-100 was added and the samples were incubated for 12 min on ice. The nuclei were pelleted by centrifugation and were resuspended in 20 μl TTBL buffer (VAHTS, TD501). Approximately 10,000 nuclei were treated with Tn5 (VAHTS, TD501) at 37 °C for 30 min. Then, the sample was immediately purified using a Qiagen MinElute kit and the purified fragments were amplified for 7–10 cycles to construct a library, according to the instructions (VAHTS, TD501). The libraries were then subjected to size-selection and sequenced (2 × 150 bp) using Illumina Hiseq X-Ten.

### ChIP-seq library preparation

ChIP-seq was performed as previously described [84] with minor modifications. Approximately 0.2 g of tissue was used for ChIP-seq assay. The fragmented chromatin was incubated with antibodies against H3K4me3 (ABclonal, A2357), which has verified the specificity [84]. ChIP DNA libraries were prepared using the NEBNext® Ultra™ II DNA Library Prep Kit for Illumina® (New England BioLabs, E7645). DNA fragments were sequenced using an Illumina HiSeq X Ten System (paired-end 150 bp reads).

### RNA-seq library preparation

Total RNA was isolated using the RNeasy Plant Mini Kit (QIAGEN, 74,904). Approximately 1 μg of RNA was used for library preparation using an Illumina TruSeq RNA kit, according to the manufacturer’s instructions. The libraries were sequenced on a BGI MGISEQ-2000 instrument with 2 × 150 bp reads.

### RNA-seq analysis

We downloaded 43 tissue RNA-seq datasets from GEO, and calculated FPKM using the following analysis pipeline. RNA-seq raw reads were processed using fastp [86] with default parameters. Clean reads were aligned to TAIR10 genome using hisat2 [87] with "–dta-cufflinks" parameters. Thereafter, we used SAMtools [88] to filter out sequences with low alignment quality (-q 30). FPKM values were calculated using StringTie [89] with default parameters. According to FPKM values from 43 tissues, we analyzed the expression breadth of H3K4me3-, H3K4me1-, H3K27me3-, and RNAPII-associated anchor and basal genes (Fig. 2f). We also analyzed the co-expression correlation of H3K4me3-, H3K4me1-, and RNAPII-mediated interacting gene pairs (Fig. 2h). The PCC of per anchor gene pair was calculated, and the mean PCC of all interacting anchor gene pairs was considered as the actual co-expression correlation coefficient. Thereafter, we randomly simulated gene pairs 1000 times and set these pairs as controls. Random gene pairs A: randomly simulated gene pairs with the same physical distance as anchor gene pairs. Random gene pairs B: randomly selected active anchor gene pairs with the same physical distance as anchor gene pairs.

### Analysis of ChIP-seq data

The quality of raw reads was evaluated and trimmed using fastp. The parameters of fastp were as follows: the window size option shared by sliding (-W) was set to 4, the mean quality requirement option shared by sliding (-M) was set to 20, the quality threshold for a qualified base (-q) was set to 15, the percentage of bases allowed to be unqualified (-u) was set to 40%, and one read’s N base number (n) was set to 5. Clean reads were mapped to the *Arabidopsis* genome (TAIR10) using Burrows-Wheeler Aligner (BWA)-mem [90] with default parameters. Duplicate reads were filtered using SAMtools. Low-quality reads were filtered using SAMtools with the mapping quality (-q) set to 30. Peak calling was performed using the MACS2 [91] tool. The parameters of MACS2 for a narrow peak model were as follows: -f BAMPE -B -g 119,667,750 -q 0.00001, with –broad -f BAMPE -B -g 119,667,750 -q 0.00001 parameters for a broad peak model.

### ChIA-PET data processing

ChIA-PET data were processed using ChIA-PET Tool V3 [92, 93], including linker filtering, read mapping, redundancy removal, and chromatin interaction identification. In the ChIA-PET Tool pipeline, we chose ChIP-seq peaks as the given anchors to call clusters. Considering the technical noise, we identified high confidence clusters by FDR < 0.05 and a given PET count, which was decided by data sequencing depth. To call chromatin interactions in Col-0 and *myc2*, we merged the H3K4me3 ChIP-seq peaks in both Col-0 and *myc2* as given anchors.

### WGBS-seq data analysis

Raw reads were analyzed using fastp to detect and filter out low-quality sequences. The parameters of fastp were the same as ChIP-seq data analysis, except that the threshold for the low complexity filter (-Y) was set to 0. Clean reads were analyzed using BatMeth2 [94] and aligned to the *Arabidopsis* genome (TAIR10). Thereafter, SAMtools was used to filter out sequences with duplicate reads and low alignment quality reads. Finally, the calmeth program in BatMeth2 was used to calculate of DNA methylation levels. Sequences with a map quality score lower than 20 were filtered out, and cytosine sites with coverage of 5 or more were considered effective methylation sites for further analysis.

### DNA FISH

The specific probes for target DNA were designed by Spatial FISH Ltd. Nuclei isolated from crosslinked (4% formaldehyde) *Arabidopsis* seedlings were spread on slides, then samples were covered with reaction chamber to perform the following reactions. After dehydration and denaturation of samples with methanol, the hybridization buffer with specific targeting probes was added to the chamber for incubation at 37℃ overnight. Then samples were washed three times with PBST, followed by ligation of target probes in ligation mix at 25℃ for 3 h. Next, samples were washed three times with PBST and subjected to rolling circle amplification by Phi29 DNA polymerase at 30℃ for overnight. Subsequently, the fluorescent detection probes in hybridization buffer were applied to samples. Finally, samples were dehydrated with an ethanol series and mounted with mounting medium, followed by observation of DNA spatial position information under a Leica TCS SP8 STED microscope. After capturing images, signal dots were decoded to interpret DNA spatial position information.

### Prediction of anchor and basal PREs and DREs

The gene annotation file (TAIR10_GFF3_genes.gff) was downloaded from the TAIR website. The genomic coordinates of proximal CREs or PREs were defined as 1 kb upstream to 0.5 kb downstream of the transcription start site (TSS) of annotated protein-coding genes. Intragenic regions refer to the region between the 0.5 kb downstream of the TSS and the transcription termination site (TTS), i.e., the gene body. The rest of the intergenic regions were annotated as distal CREs or DREs. CREs were assigned to each category using a 50% peak overlap. If a peak overlaps multiple categories, only one category is used based on the following priority: PREs > DREs > intragenic regions.

The categories of anchor and basal CREs were defined based on chromatin interactions. To link CREs with their associated genes, each CRE was assigned to its nearest TSS of annotated protein-coding genes. If DRE-associated nearest (< 500 bp) anchor peak was H3K27me3 only, we defined this DRE as an anchor poised DRE. If DRE-associated nearest (< 500 bp) basal peak was H3K27me3 only, we defined this DRE as a basal poised DRE. Excluding poised DRE, if the remaining DRE-associated nearest protein-coding gene was anchor gene, we defined this DRE as an anchor active DRE. If PRE overlapped H3K27me3 anchor peak only, we defined this PRE as an anchor poised PRE. If PRE overlapped H3K27me3 and active anchor peak simultaneously, we defined this PRE as an anchor poiAct PRE. If PRE linked a basal gene, we defined this PRE as a basal PRE. Excluding poised and poiAct PRE and basal PRE, the remaining PREs were defined as anchor active PREs.

### A/B compartment

We used an eigenvector program from juicer software [95] to delineate A/B compartments in ChIA-PET data at 25, 10, and 5 kb resolution. In this study, each chromosome was divided into fixed windows at coarse resolution. The first principal component of the correlation matrix indicated the compartments.

### Construction of contact maps

We obtained the ChIA-PET contact matrix using the bedpe2Matrix program of ChIA-PET2 software [92, 96] at 25, 10, and 5 kb resolution with “–all –matrix-format complete” parameters from the ChIA-PET unique mapping reads, and the matrix was normalized by iterative correction and eigenvector decomposition using HiC-Pro [97]. The matrix was normalized and visualized using HiCExplorer [98].

### Analysis of chromatin states

ChromHMM v1.12 [59], a multivariate Hidden Markov Model, was used for unsupervised segmentation of the *Arabidopsis* genome into a certain number of states based on the combination of histone modifications and CRE. The genome was divided into 200 bp bins. Four histone marks and CRE were used to divide the *Arabidopsis* genome into 10 states, since they captured all the key information of chromatin states.

### Hub analysis

Regulatory element-associated chromatin loops form spatial clusters, in which multiple interactions could be seen emanating from a single anchor site termed the node gene; the divergent interaction sites were considered the connecting genes. First, the chromatin hub was defined as chromatin interactions with contact frequency (degree) >  = 5. Thereafter, TF target genes distribution in each hub were count and the enrichment of TF target genes in each hub was calculated using Fisher’s exact test. The hub was screened as a TF enrichment one with *p* value < 0.05 and TF target genes in the enrichment hubs were counted. Random analysis: according to the size of all hubs enriched by each TF target genes, we selected the same number of genes from all anchor genes to form a new hub. The enrichment of the TF target genes in the hub was calculated and the TF-enriched one was finally screened with *p* value < 0.05. Lastly, the TF target genes in the enriched hub were counted as a control.

We used bedtools pairtopair program of BEDTools software [99] to calculate the differences in chromatin loops between wild-type and *myc2*. Additionally, we analyzed the differences of hubs between wild-type and *myc2*. For this analysis, we defined a chromatin hub with a contact frequency (degree) of 5 or more. We considered hubs shared the same node genes as the same hub and compared the changes of connecting genes between wild-type and *myc2*. We defined the number of connecting genes in wild-type as N_c_ and the number of connecting genes in *myc2* as N_m_. We calculated the connecting gene change as N_s_ = N_c_-N_m_. Furthermore, we defined entirely disassembled hubs (EDH) as:

$$EDH= \left\{\begin{array}{c}\frac{Ns}{Nc}\ge 0.7\\ Ns\ge 3\end{array}\right.$$

Partially disassembled hubs (PDH) as:

$$PDH= \left\{\begin{array}{c}\frac{Ns}{Nc}\ge 0.3\\ \frac{Ns}{Nc}<0.7\\ Ns\ge 3\end{array}\right.$$

Newly formed or markedly increased in size hubs (FH) as:

$$FH= \left\{\begin{array}{c}\frac{-Ns}{Nm}\ge 0.3\\ Nc-Nm\le -3\end{array}\right.$$

The resthubs were defined as stable hubs.

### Chromatin connectivity network analysis

We merged active anchors to produce a list of non-overlapping chromatin interactions. If an anchor overlapped the region between the 1 kb upstream of the TSS and the TTS of annotated protein-coding genes by 50%, we defined this gene as an anchor gene. If an anchor overlapped two or more genes, we selected the gene with the highest FPKM value as the anchor gene candidate. Regarding H3K27me3 associated-anchor, if the anchor overlapped the region between the 1 kb upstream of the TSS and the TTS of annotated protein-coding genes by 50%, we defined this gene as an anchor gene. If an anchor overlapped two or more genes, we retained all the genes as anchor gene candidates. The chromatin connectivity networks were constructed through one hop or two hops of active mark- and H3K27me3-associated interacting gene pairs originating from flowering time regulator genes. Nodes were connected based on chromatin interactions in the ChIA-PET libraries and visualized using Gephi [100]. Embedded meta-information was used for color coding.

### Gene ontology analysis

Gene Ontology (GO) term enrichment was performed using the AmiGO online toolkit [101–103].

### Supplementary Information

**Additional file 1: Fig. S1.** ChIA-PET data reproducibility. **Fig. S2.** Validation of chromatin interactions by DNA-FISH and aggregate chromosome analysis (ACA) in *Arabidopsis*. **Fig. S3.** Comparison of chromatin interaction patterns based on ChIA-PET and Hi-C data in *Arabidopsis*. **Fig. S4.** Inactive chromatin loops of the indicated chromatin regions. **Fig. S5.** Features of epigenetic modifications and gene transcription in compartments A and B. **Fig. S6.** Characterization of chromatin interactions. **Fig. S7.** Global patterns of H3K4me3-associated peaks and chromatin interactions in *Arabidopsis* and rice. **Fig. S8.** Global patterns of RNAPII-associated peaks and chromatin interactions in *Arabidopsis* and rice. **Fig. S9.** Global patterns of H3K9me2-associated peaks and chromatin interactions in *Arabidopsis* and rice. **Fig. S10.** Global patterns of H3K4me1- and H3K27me3-associated peaks and chromatin interactions in *Arabidopsis*. **Fig. S11.** Expression level among genes with different binding (basal) and interactions (anchor) patterns. **Fig.** **S12.** High-order chromatin organization in *Arabidopsis*. **Fig. S13.** Reproducibility of ATAC-seq analysis and epigenomic identification of different clusters of basal CREs in *Arabidopsis*. **Fig. S14.** Effects of CREs on the expression of nearest and long-range genes. **Fig. S15.** Transcription factors (TFs)-associated chromatin topology and its transcriptional function. **Fig. S16.** Assessment of reproducibility between two ChIA-PET biological replicates. **Fig. S17.** Characterization of chromatin hubs. **Fig. S18.** Connectivity networks converged by flowering-time control genes. **Fig.** **S19.** Enriched GO terms of genes involved in one hop (A) and two hops (B) of active mark-associated 3D network, repressive mark-associated 3D network, and mixed 3D network.

**Additional file 2: Table S1.** Summary of ChIA-PET libraries.

**Additional file 3: Table S2.** Chromatin interactions validated by DNA fluorescence in situ hybridization.

**Additional file 4: Table S3.** *Arabidopsis* RNA-seq data of different tissues used in this study.

**Additional file 5: Table S4.** Distribution of the indicated TF targets in each hub.

**Additional file 6: Table S5.** Summary of H3K4me3 ChIA-PET, H3K4me3 ChIP-seq, and RNA-seq libraries in Col-0 and *myc2*.

**Additional file 7: Table S6.** List of the long-range chromatin interactions spatially linking SNPs to flowering-time-associated target genes.

**Additional file 8: Table S7.** Summary of flowering-time control genes used in this study.

**Additional file 9: Table S8.** One-hop interactions mediated by genes implicated in flowering-time control.

**Additional file 10: Table S9.** Two-hop interactions mediated by genes implicated in flowering-time control.

**Additional file 11: Table S10.** Summary of ChIP-seq, RNA-seq, and DNA methylation data used in this study.

**Additional file 12. **Review history.

## Data Availability

The raw data of ChIA-PET, ATAC-seq, ChIP-seq, and RNA-seq are available at NCBI GEO under accession number GSE207010 [104] and GSE233528 [105]. The ChIP-seq of histone modifications and WGBS raw datasets are available at NCBI GEO under accession number GSE183987 [106, 107]. The RNA-seq and TF ChIP-seq raw datasets were collected from NCBI GEO, and accession numbers are listed in Additional file 4: Table S3 and Additional file 11: Table S10. No other scripts and software were used other than those mentioned in the Methods section. All data supporting the findings of this study are available within the manuscript and its supporting information are available from the corresponding author upon request.
