# Supplementary material for: 3D organization of regulatory elements for transcriptional regulation in Arabidopsis
Source: Genome Biol. 2023 Aug 7;24:181. doi: 10.1186/s13059-023-03018-4 (PMC10405511; doi:10.1186/s13059-023-03018-4)
Supplement: Supplementary file 1 — Additional file 1: Fig. S1. ChIA-PET data reproducibility. Fig. S2. Validation of chromatin interactions by DNA-FISH and aggregate chromosome analysis (ACA) in Arabidopsis. Fig. S3. Comparison of chromatin interaction patterns based on ChIA-PET and Hi-C data in Arabidopsis. Fig. S4. Inactive chromatin loops of the indicated chromatin regions. Fig. S5. Features of epigenetic modifications and gene transcription in compartments A and B. Fig. S6. Characterization of chromatin interactions. Fig. S7. Global patterns of H3K4me3-associated peaks and chromatin interactions in Arabidopsis and rice. Fig. S8. Global patterns of RNAPII-associated peaks and chromatin interactions in Arabidopsis and rice. Fig. S9. Global patterns of H3K9me2-associated peaks and chromatin interactions in Arabidopsis and rice. Fig. S10. Global patterns of H3K4me1- and H3K27me3-associated peaks and chromatin interactions in Arabidopsis. Fig. S11. Expression level among genes with different binding (basal) and interactions (anchor) patterns. Fig. S12. High-order chromatin organization in Arabidopsis. Fig. S13. Reproducibility of ATAC-seq analysis and epigenomic identification of different clusters of basal CREs in Arabidopsis. Fig. S14. Effects of CREs on the expression of nearest and long-range genes. Fig. S15. Transcription factors (TFs)-associated chromatin topology and its transcriptional function. Fig. S16. Assessment of reproducibility between two ChIA-PET biological replicates. Fig. S17. Characterization of chromatin hubs. Fig. S18. Connectivity networks converged by flowering-time control genes. Fig. S19. Enriched GO terms of genes involved in one hop (A) and two hops (B) of active mark-associated 3D network, repressive mark-associated 3D network, and mixed 3D network. [file 13059_2023_3018_MOESM1_ESM.pdf]

3D organization of regulatory elements for transcriptional regulation in *Arabidopsis*

Supplementary Figures

Deng et al., 2023

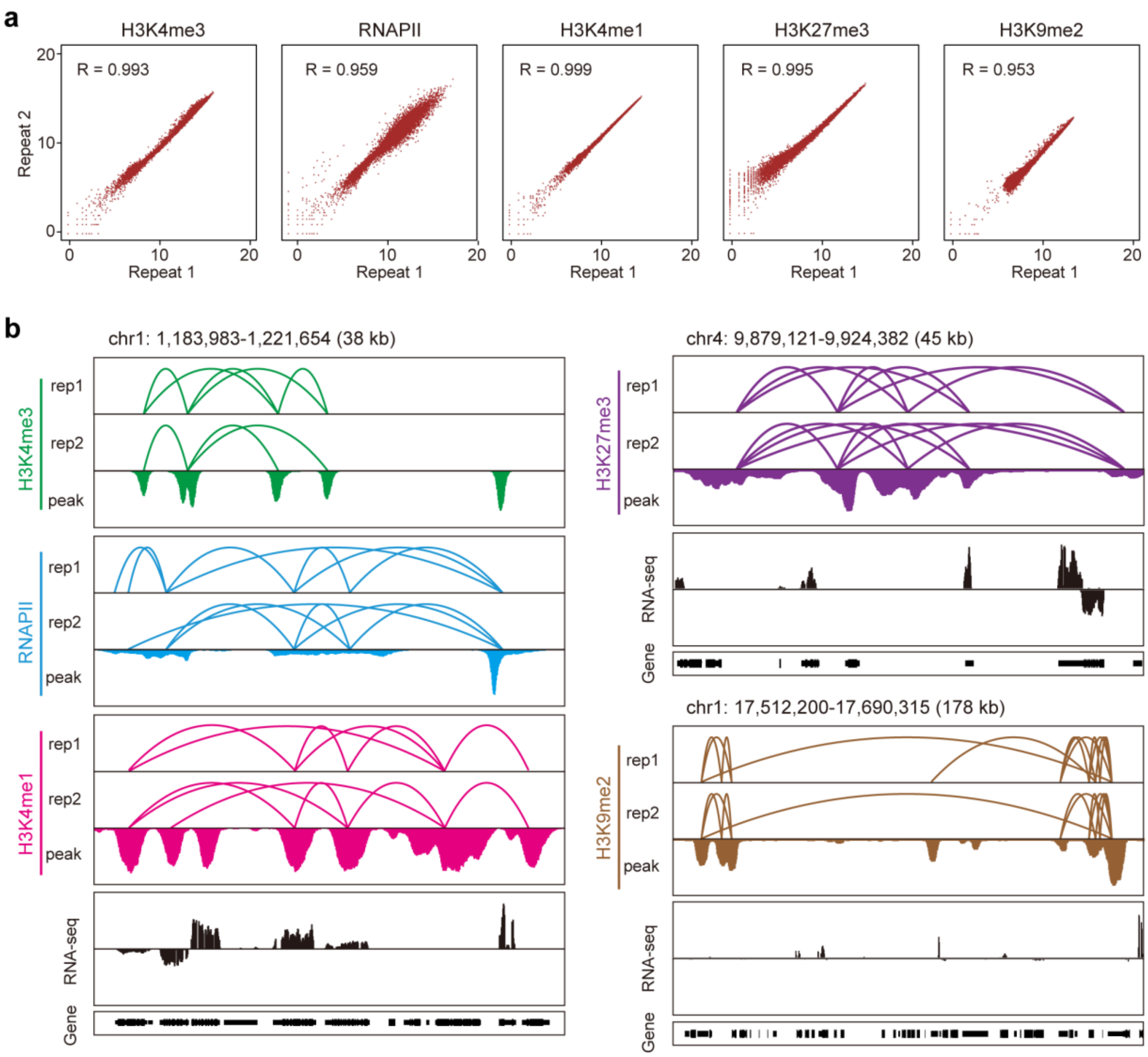

Fig S1. ChIA-PET data reproducibility. **a** Scatter plots showing the reproducibility between two ChIA-PET replicates of H3K4me3, RNAPII, H3K4me1, H3K27me3, and H3K9me2. Spearman correlation coefficients are shown. **b** Genome browser at the indicated region showing the high reproducibility of ChIA-PET data.

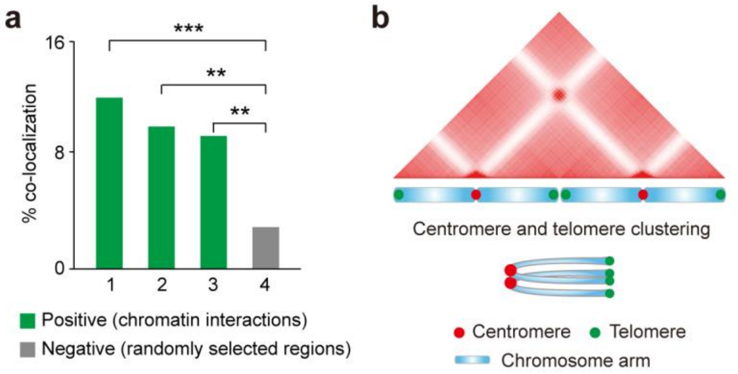

Fig S2. Validation of chromatin interactions by DNA-FISH and aggregate chromosome analysis (ACA) in *Arabidopsis*. **a** Quantitative DNA FISH data for positive (interaction) and negative (no interaction) hits selected from *Arabidopsis* ChIA-PET data. 200 nuclei were counted for each DNA FISH experiment. ***$p < 0.001$, **$p < 0.01$ from the two-sided Fisher's exact test. **b** Aggregate chromosome analysis (ACA) map and a schematic drawing of the chromosome scale features of nuclear architecture in *Arabidopsis*. ACA map was visualized using Juicebox.js.

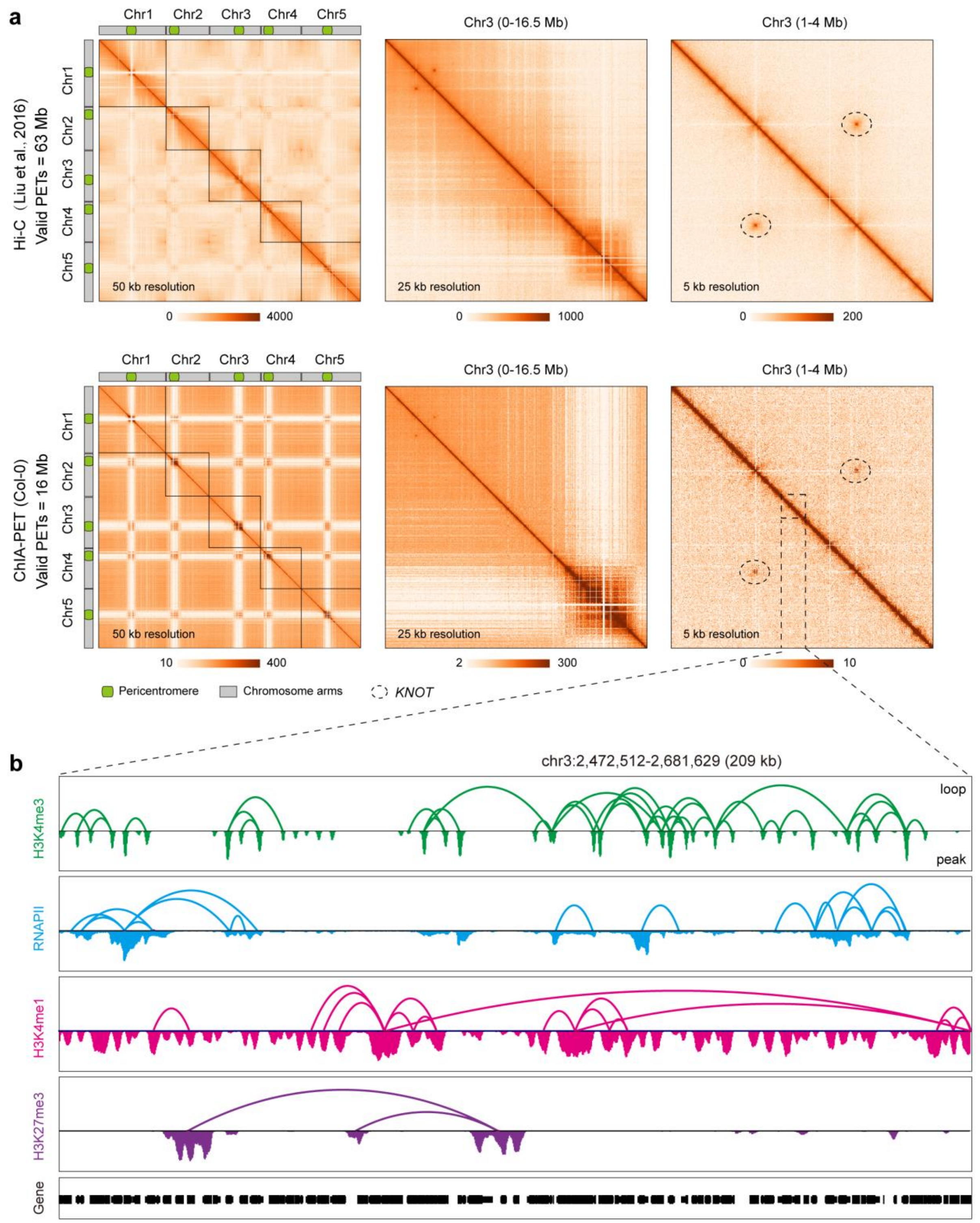

Fig S3. Comparison of chromatin interaction patterns based on ChIA-PET and Hi-C data in *Arabidopsis*. **a** Comparison of ChIA-PET data and Hi-C data from Liu et al. ChIA-PET recapitulates the primary chromatin structures as previously reported in Hi-C data. Pericentromeric regions are shown as heterochromatic compartments. Black dotted ovals indicate the *KNOT* nuclear structure. Genome-wide contact heatmaps in *Arabidopsis* at 50, 25, and 5 kb resolution. Elements represent normalized contact strength. **b** Chromatin

loops and binding peaks of the indicated factor in the box region of panel (**a**). The data tracks show chromatin loops and profiling of representative histone modifications and RNAPII occupancy.

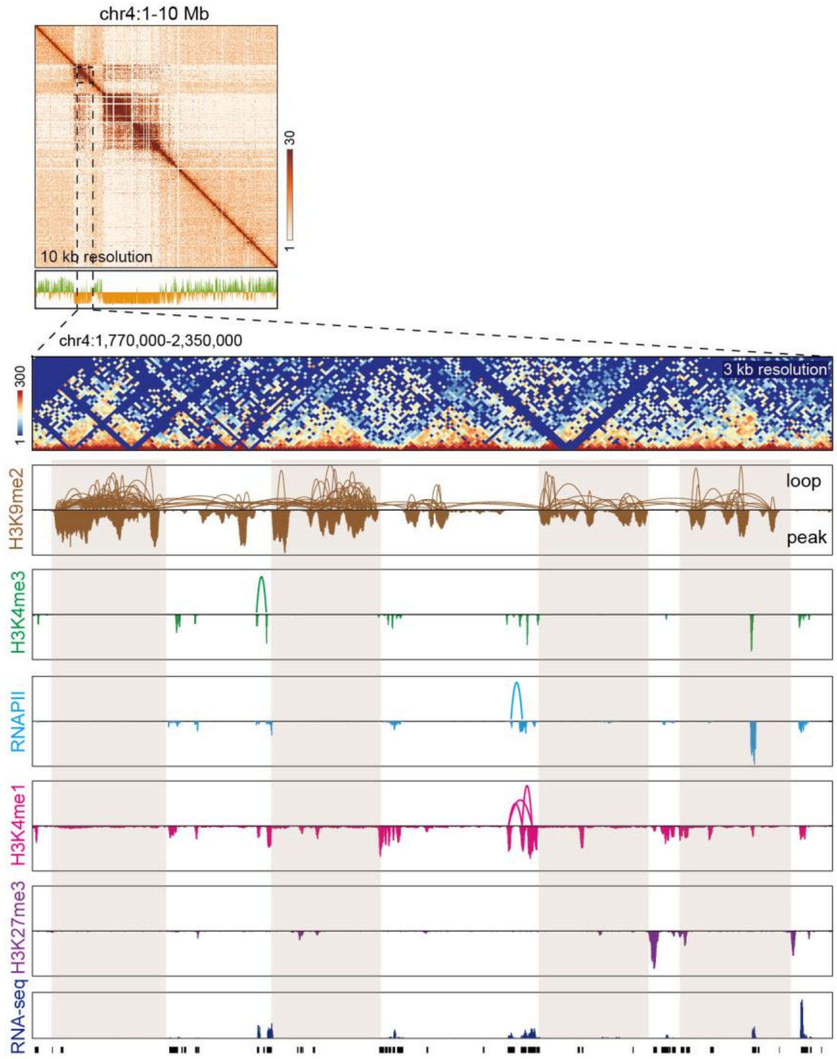

Fig S4. Inactive chromatin loops of the indicated chromatin regions.

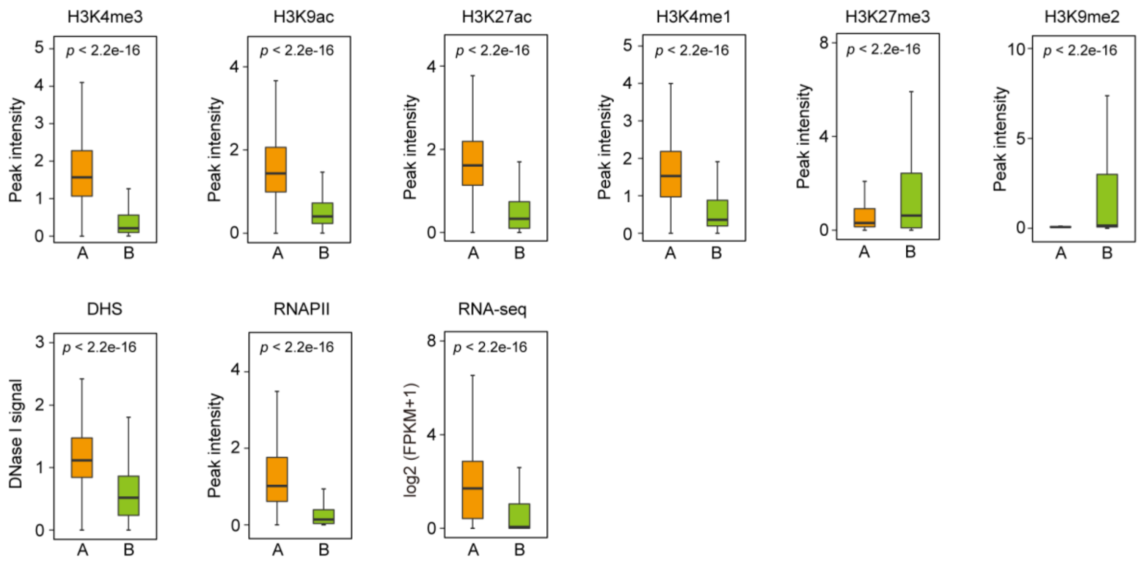

Fig S5. Features of epigenetic modifications and gene transcription in compartments A and B. Boxplots show the median, third and first quartiles. DHS, DNase I hypersensitive sites.

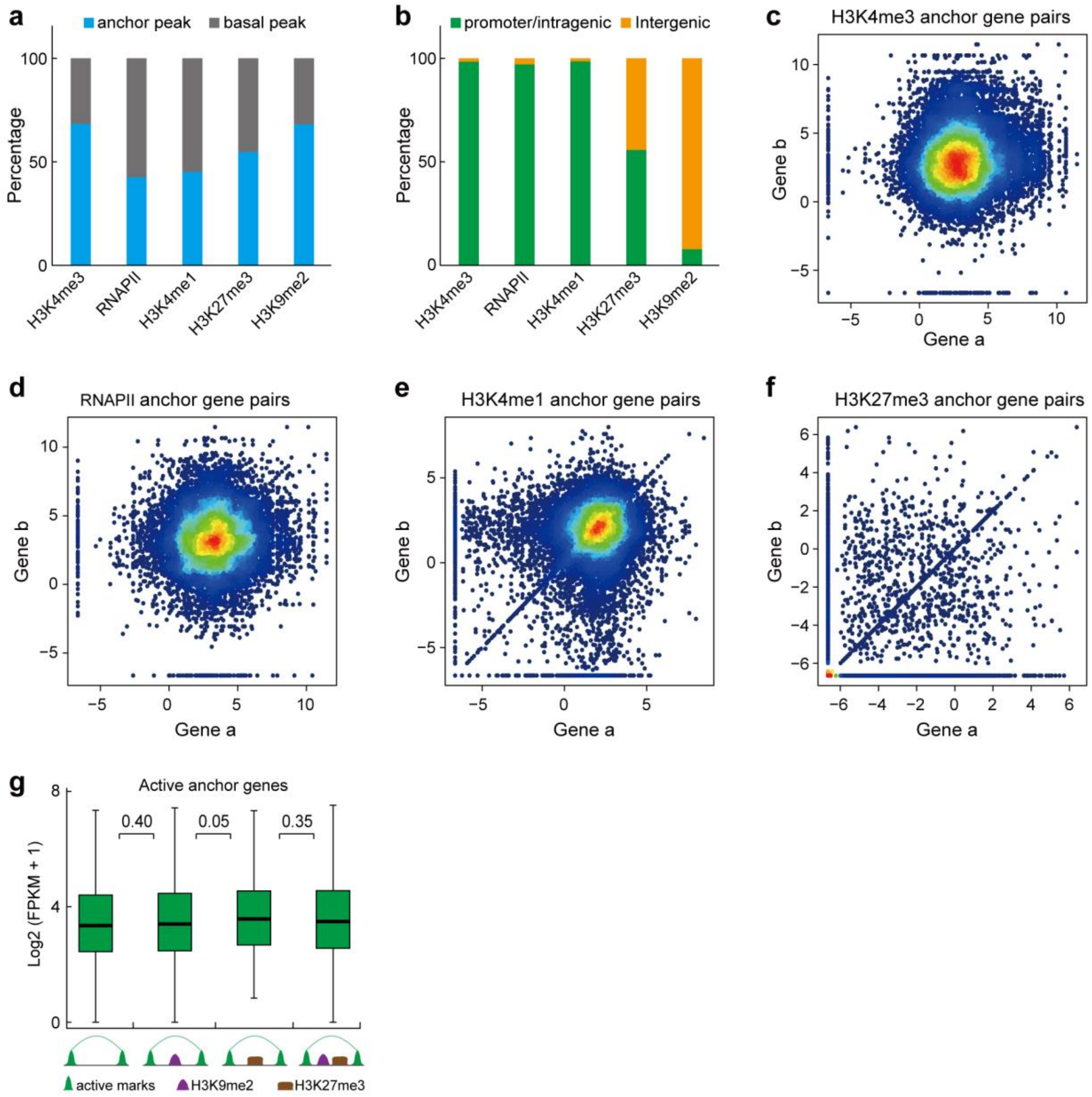

Fig S6. Characterization of chromatin interactions. **a** Percentages of anchor and basal peaks (binding sites) of the indicated factors. **b** Genome distribution of the indicated factor binding sites. **c**–**f** Scatter plot of log-transformed FPKM values for H3K4me3 (**c**), RNAPII (**d**), H3K4me1 (**e**), and H3K27me3 (**f**) anchor gene pairs. **g** Expression levels of active anchor genes with loops spanning or not spanning H3K27me3 and/or H3K9me2 peaks.

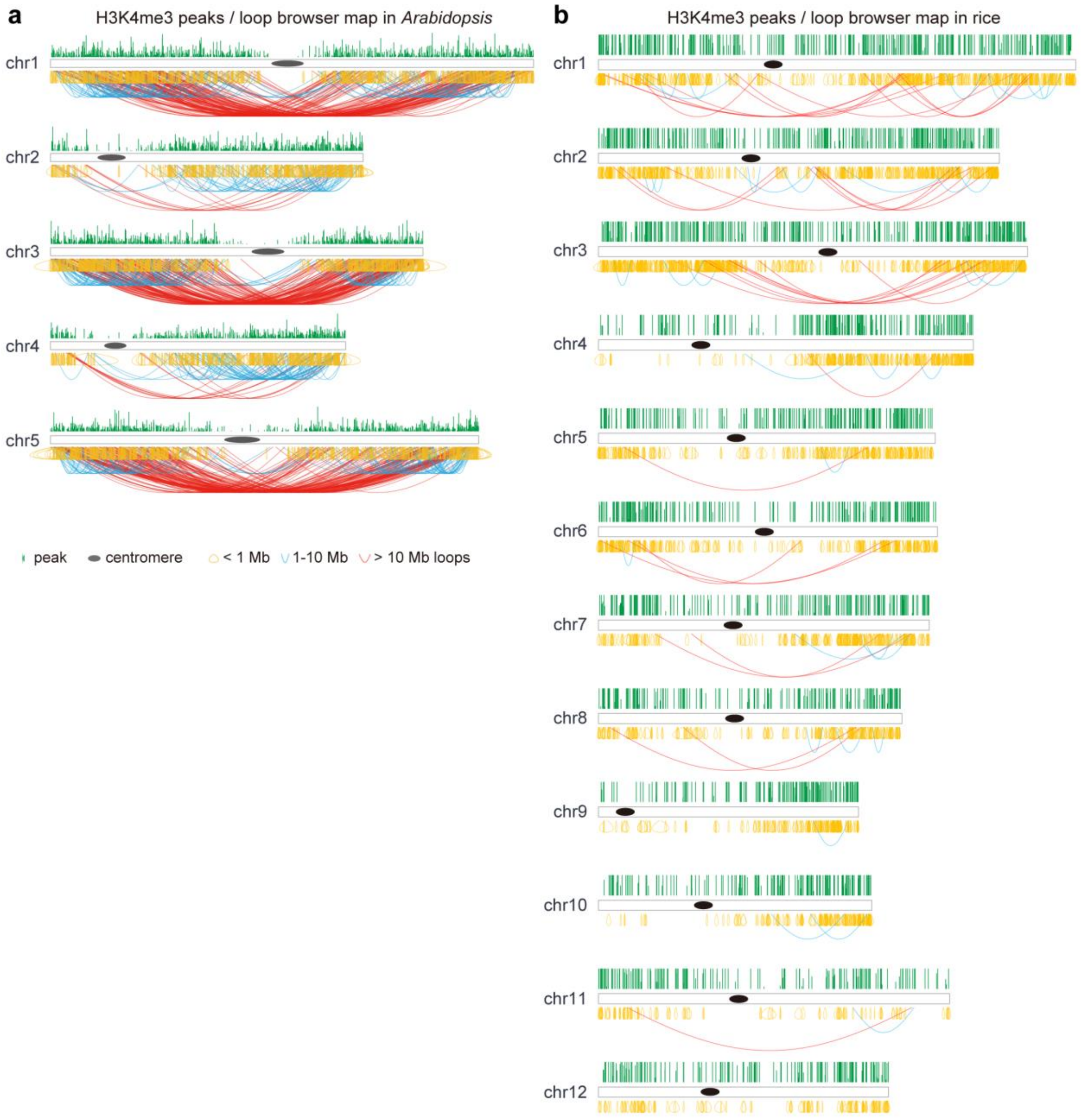

Fig S7. Global patterns of H3K4me3-associated peaks and chromatin interactions in *Arabidopsis* and rice. Global views of H3K4me3-associated peaks and intrachromosomal interactions in *Arabidopsis* (**a**) and rice (**b**). Green peaks above chromosomes indicate binding sites, and curves under chromosomes indicate interactions. Yellow, blue, and red curves indicate interaction spans smaller than 1 Mb, between 1 to 10 Mb, and larger than 10 Mb, respectively.

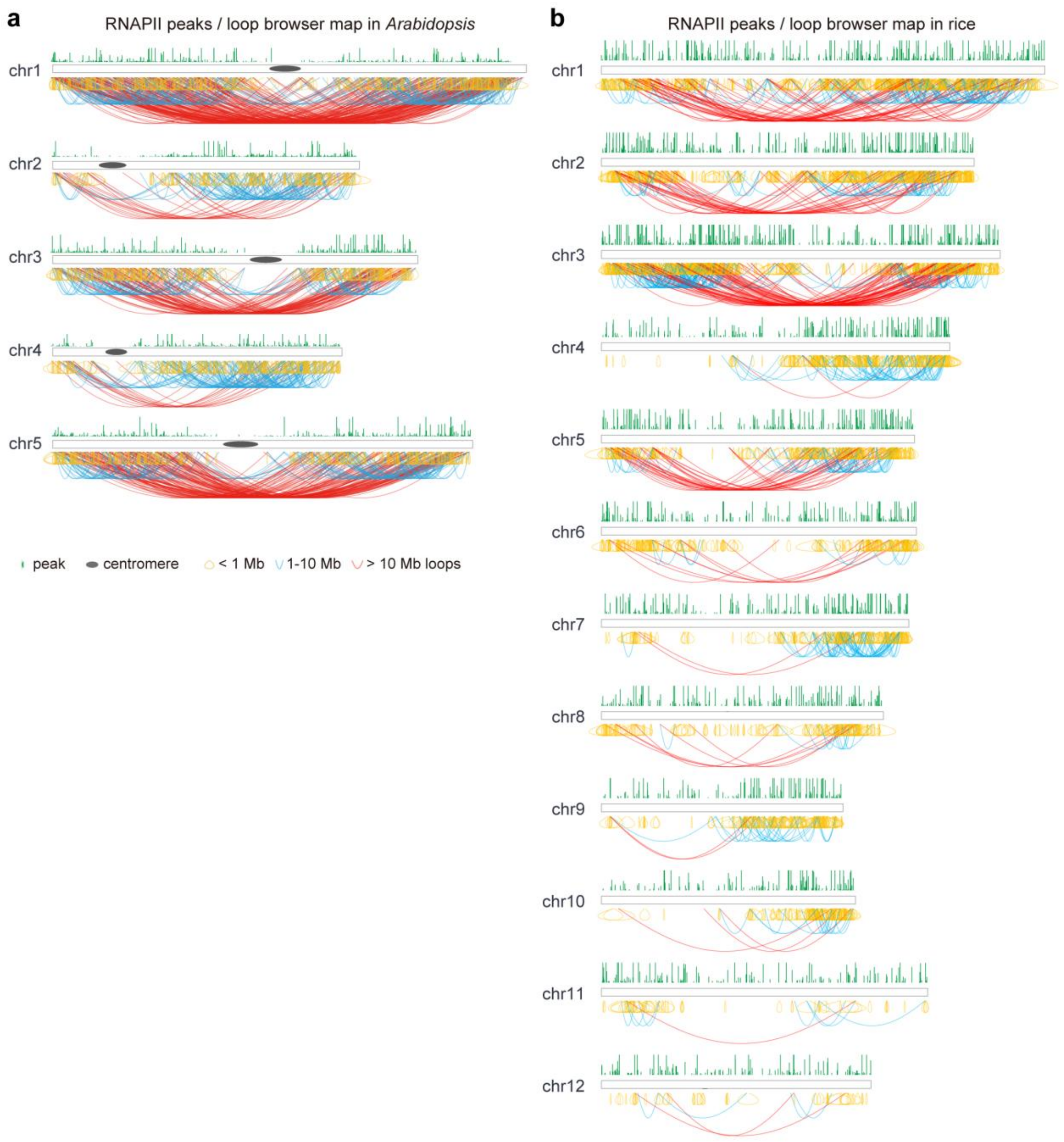

Fig S8. Global patterns of RNAPII-associated peaks and chromatin interactions in *Arabidopsis* and rice. Global views of RNAPII-associated peaks and intrachromosomal interactions in *Arabidopsis* (**a**) and rice (**b**). Green peaks above chromosomes indicate binding sites, and curves under chromosomes indicate interactions. Yellow, blue, and red curves indicate interaction spans smaller than 1 Mb, between 1 to 10 Mb, and larger than 10 Mb, respectively.

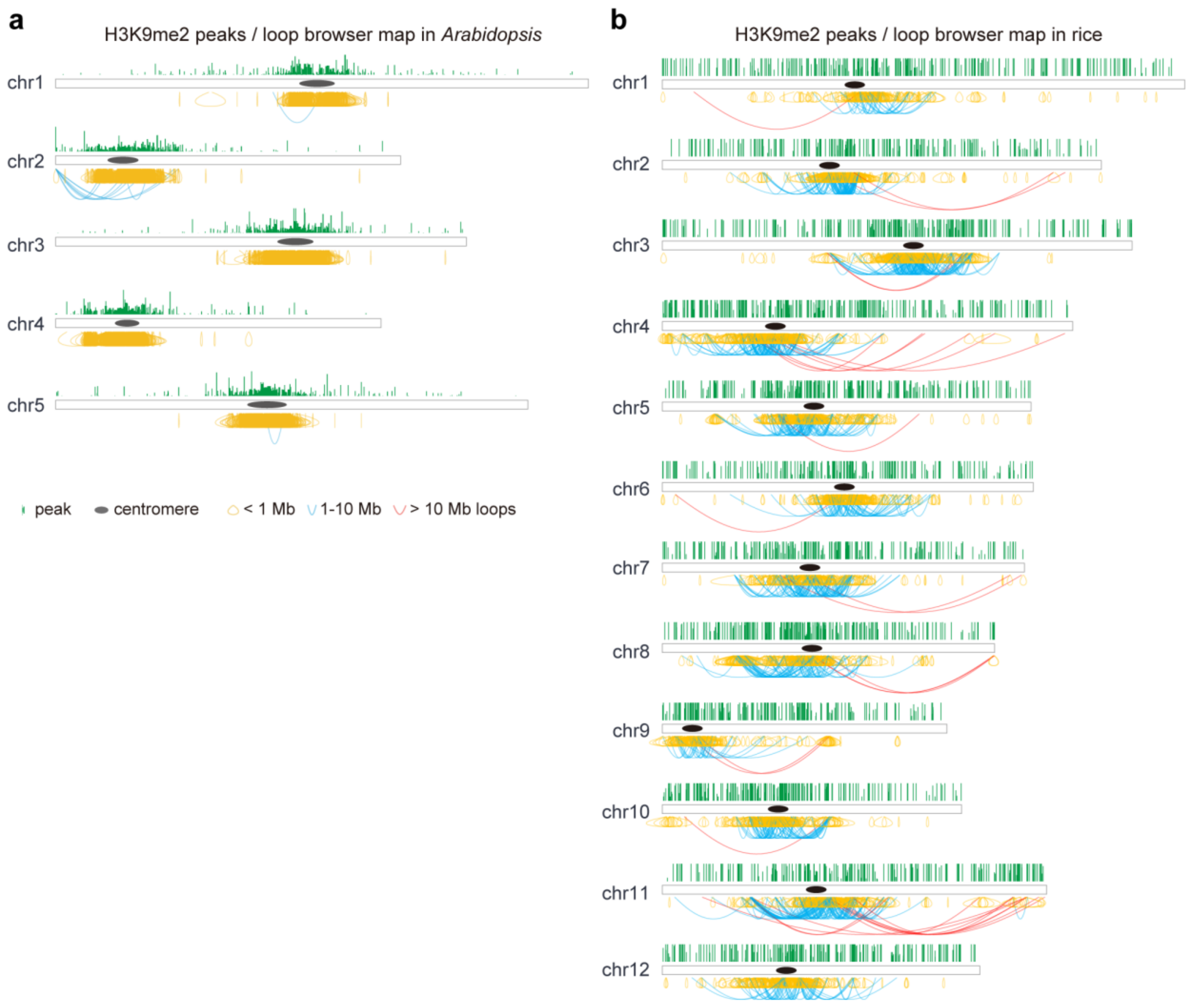

Fig S9. Global patterns of H3K9me2-associated peaks and chromatin interactions in *Arabidopsis* and rice. Global views of H3K9me2-associated peaks and intrachromosomal interactions in *Arabidopsis* (**a**) and rice (**b**). Green peaks above chromosomes indicate binding sites, and curves under chromosomes indicate interactions. Yellow, blue, and red curves indicate interaction spans smaller than 1 Mb, between 1 to 10 Mb, and larger than 10 Mb, respectively.

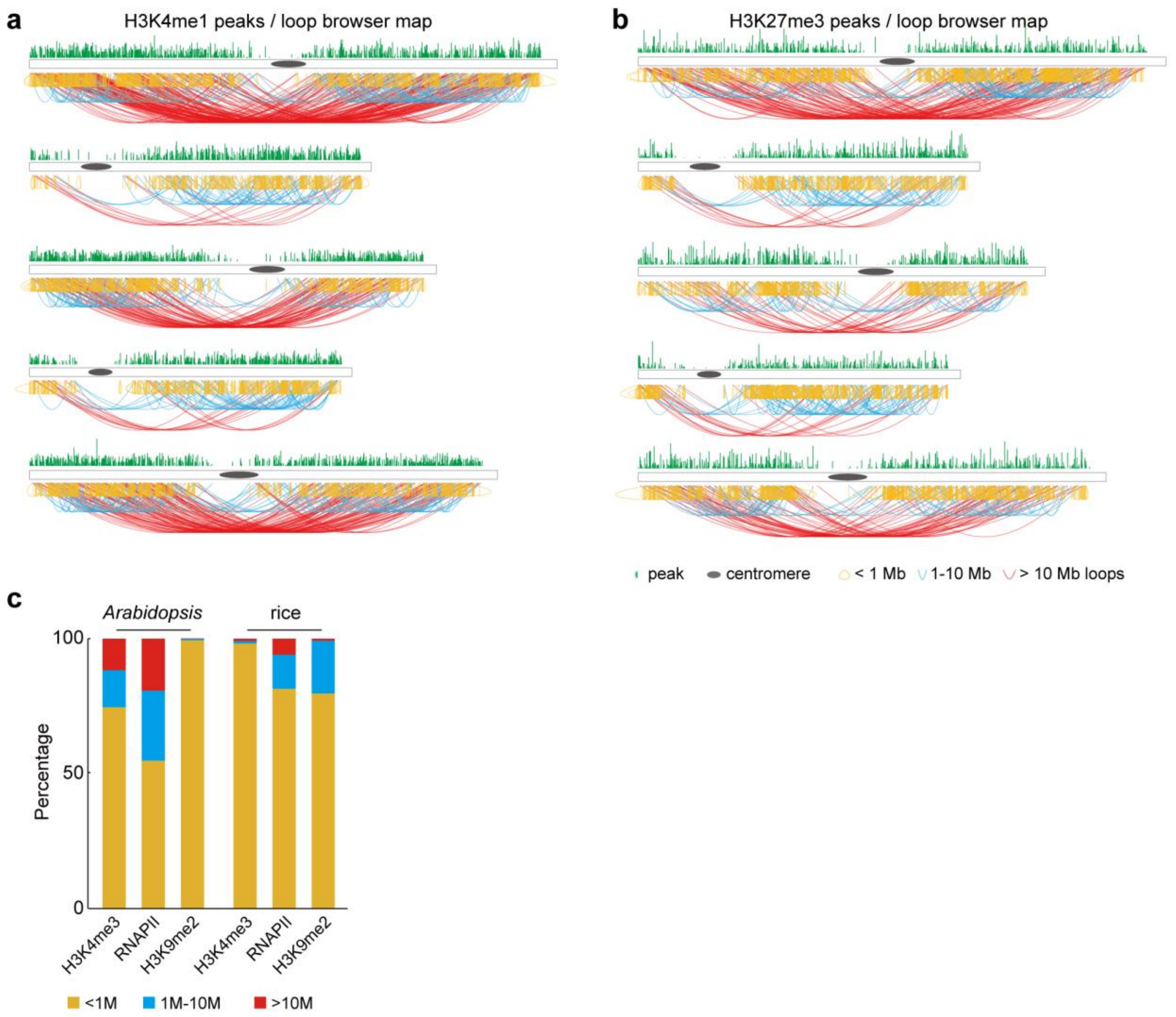

Fig S10. Global patterns of H3K4me1- and H3K27me3-associated peaks and chromatin interactions in *Arabidopsis*. **a**, **b** Global patterns of H3K4me1-associated (**a**) and H3K27me3-associated (**b**) peaks and chromatin interactions in *Arabidopsis*. Green peaks above chromosomes indicate binding sites, and curves under chromosomes indicate interactions. Yellow, blue, and red curves indicate interaction spans smaller than 1 Mb, between 1 to 10 Mb, and larger than 10 Mb, respectively. **c** Bar chart shows the percentage of H3K4me3-, RNAPII-, and H3K9me2-associated chromatin interactions with various loop spans in *Arabidopsis* and rice.

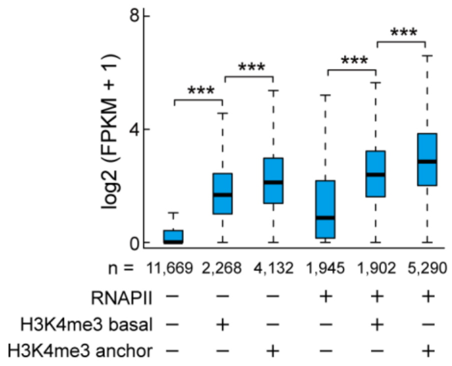

Fig S11. Expression level among genes with different binding (basal) and interactions (anchor) patterns. ****p* < 2.2e-16 from Wilcoxon test.

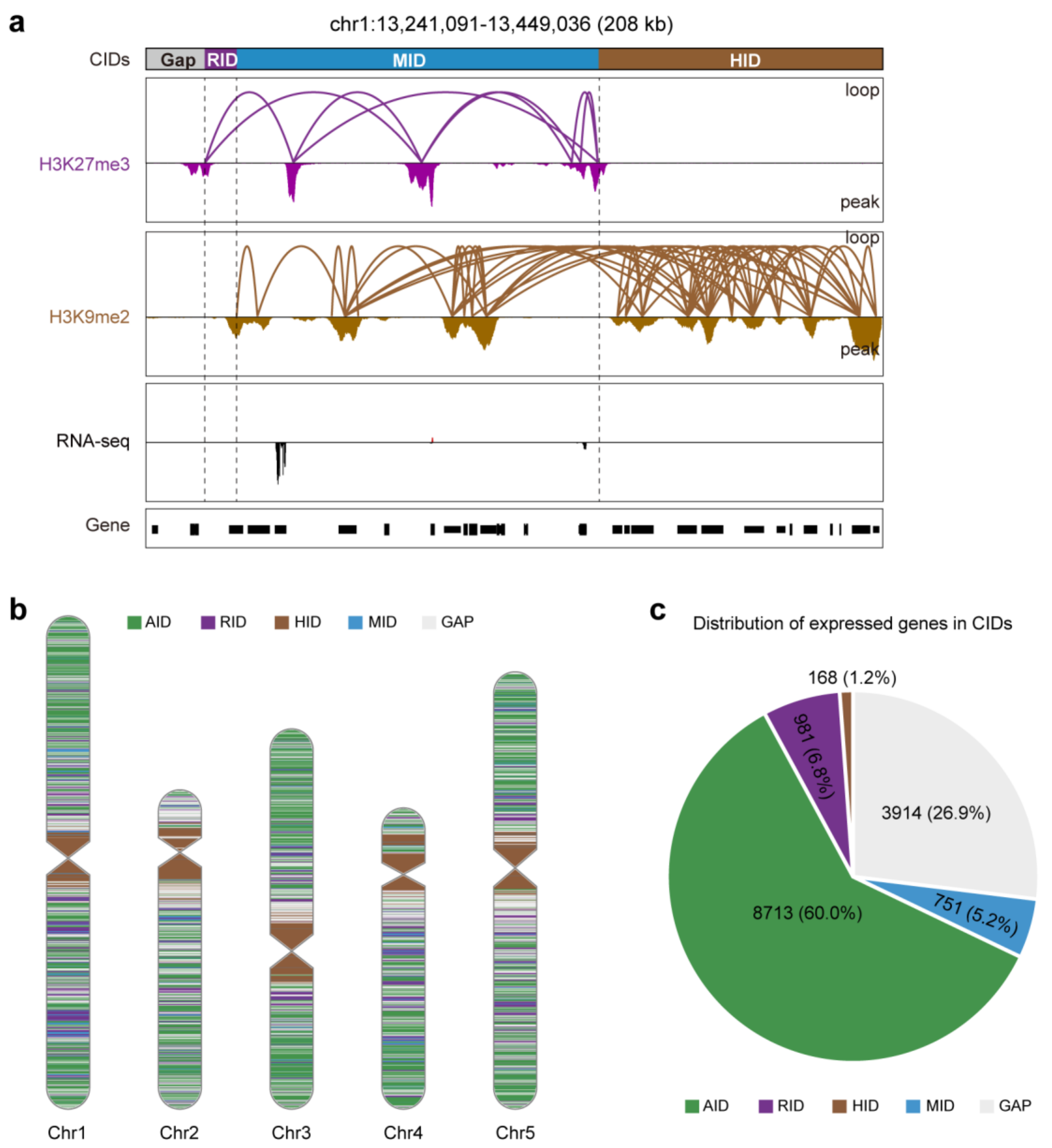

Fig S12. High-order chromatin organization in *Arabidopsis*. **a** Browser view of different CIDs showing interval arrangement. **b** Global distribution of CIDs in each *Arabidopsis* chromosome. The data tracks show CIDs, chromatin loops, profiling of representative histone modifications, and gene transcription. **c** Distribution of expressed genes (fragments per kilobase per million mapped fragments in exons > 1) in CIDs.

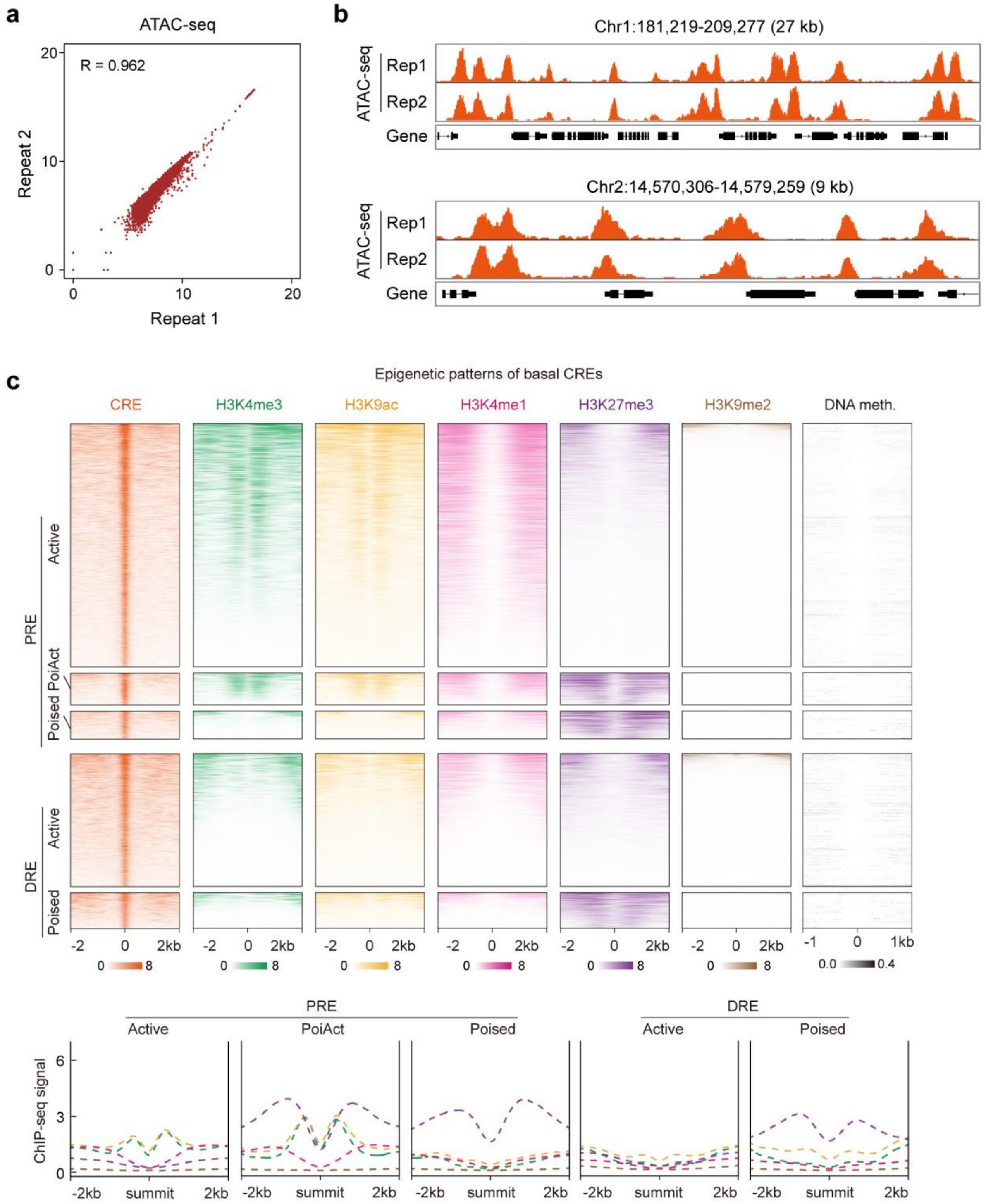

Fig S13. Reproducibility of ATAC-seq analysis and epigenomic identification of different clusters of basal CREs in *Arabidopsis*. **a** Pearson correlation for two ATAC-seq biological replicates. The R value calculated by Pearson correlation coefficient at 10 kb genomic bin is shown. **b** Browser screenshot showing the chromatin accessibility in the indicated region between two ATAC-seq replicates. **c** Epigenome heatmaps (upper) and profiles (lower) of different clusters of basal CREs. Regions shown are ± 2 kb (DNA methylation are ± 1 kb) from ATAC-seq peak summits.

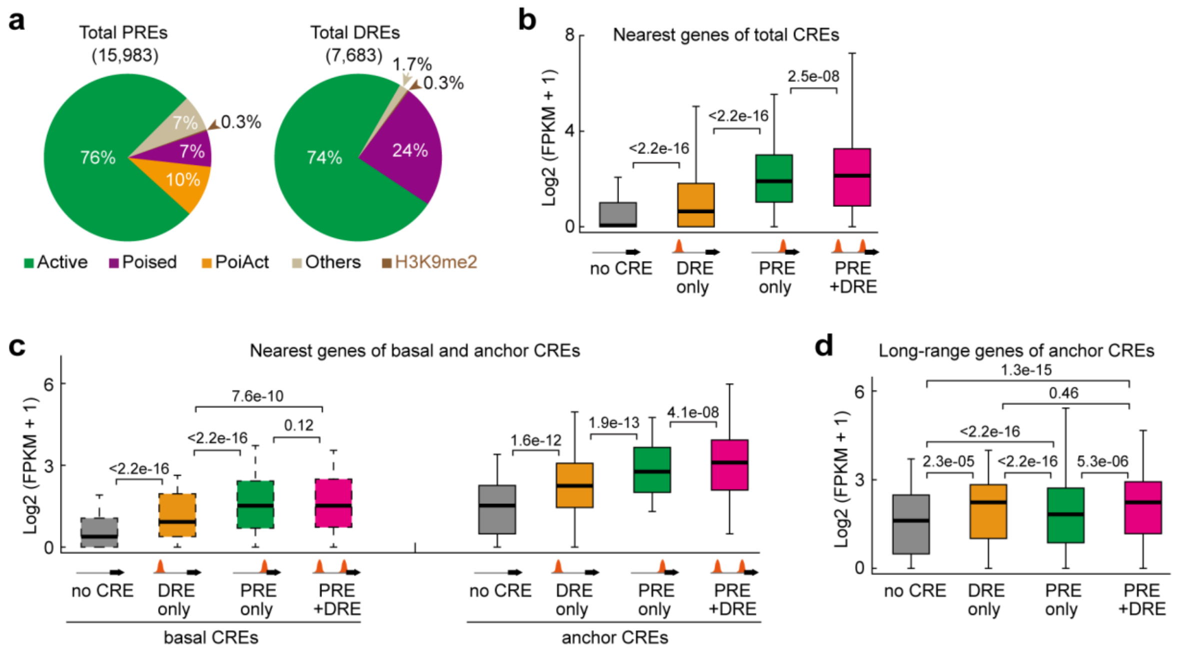

Fig S14. Effects of CREs on the expression of nearest and long-range genes. **a** Percentages of PREs and DREs that with distinct chromatin signals. **b** Expression of genes associated with only PREs, DREs, or both. Total CREs, including basal CREs and anchor CREs, were counted together in each category. **c** Expression levels of basal and anchor CREs-associated nearest genes with only PREs, DREs, or both. **d** Expression level of anchor CRE-associated long-range connecting genes with only PREs, DREs, or both.

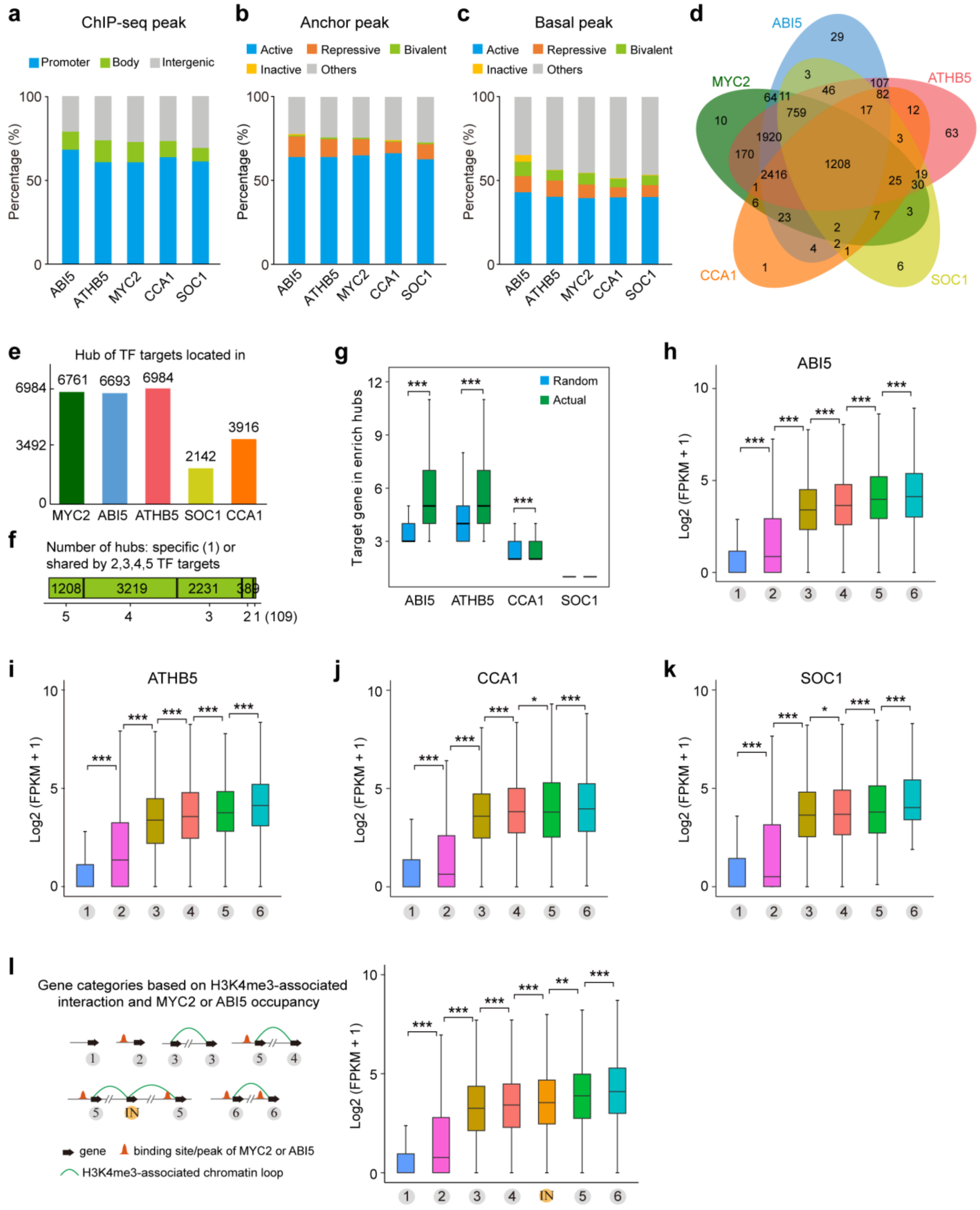

Fig S15. Transcription factors (TFs)-associated chromatin topology and its transcriptional function. **a** Genome distribution of the indicated TF-associated ChIP-seq peaks. **b** Percentages of the indicated TF ChIP-seq peak-associated anchor peaks in active, repressive, bivalent, inactive, and other regions. **c** Percentages of the indicated TF ChIP-seq peak-associated basal peaks in active, repressive, bivalent, inactive, and other regions. **d** Venn diagram showing the distribution of the indicated TF target genes in the chromatin hubs. **e**

Number of hubs of each indicated TF target genes distribution. **f** Number of hubs of each indicated TF target genes distribution, specific (1) or shared by 2, 3, 4, or 5 TF target genes. **g** Distribution of the number of the indicated TF target genes and randomly selected genes in chromatin hubs. ***$p < 2.2e\text{-}16$ from Wilcoxon test. **h–k** Expression levels of genes in different categories based on H3K4me3-associated chromatin interaction models and the indicated TF binding sites. ***$p < 0.001$, *$p < 0.05$ from Wilcoxon test. **l** Expression levels of genes in different categories based on H3K4me3-associated chromatin interaction models and MYC2 or ABI5 occupancy. IN, independent on MYC2 or ABI5 occupancy. ***$p < 0.001$, **$p < 0.01$ from Wilcoxon test.

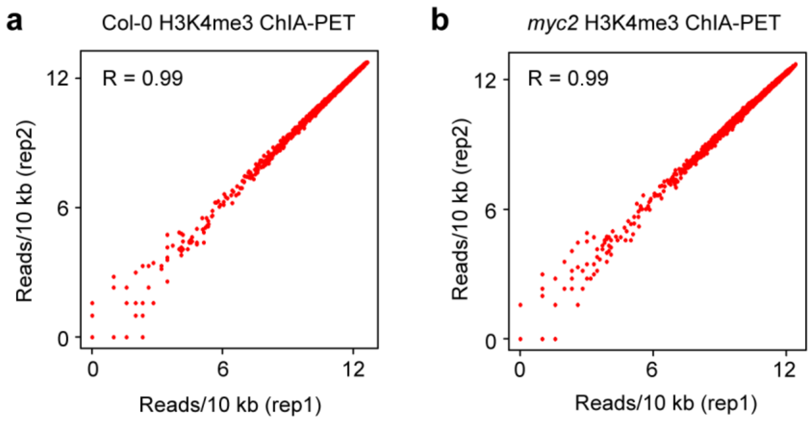

Fig S16. Assessment of reproducibility between two ChIA-PET biological replicates. **a**, **b** Scatter plots of sequence reads per 10 kb from two H3K4me3 ChIA-PET replicates in Col-0 (wild-type) (**a**) and *myc2* (**b**). Spearman correlation coefficients are shown.

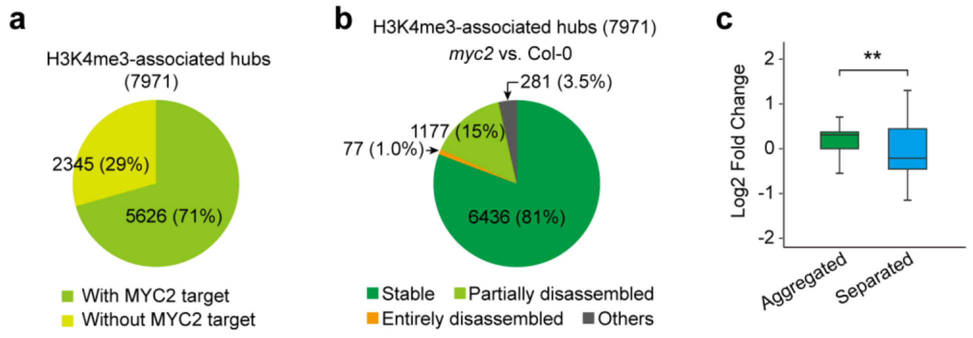

Fig S17. Characterization of chromatin hubs. **a** Number and percentage of H3K4me3-associated chromatin hubs contained or not contained MYC2 ChIP-seq target genes. **b** Changes of H3K4me3-associated chromatin hubs in *myc2* mutant compared to wild-type. These hubs were classified into four categories: stable, entirely and partially disassembled, and others. The number and proportion of hubs included in each category were given. **c** Log2 fold change in expression of aggregated and separated genes defined in the disassembled hubs. Fold-changes represent the expression changes of each gene in *myc2* mutant relative to the wild-type. ***p* < 0.01 from Wilcoxon test.

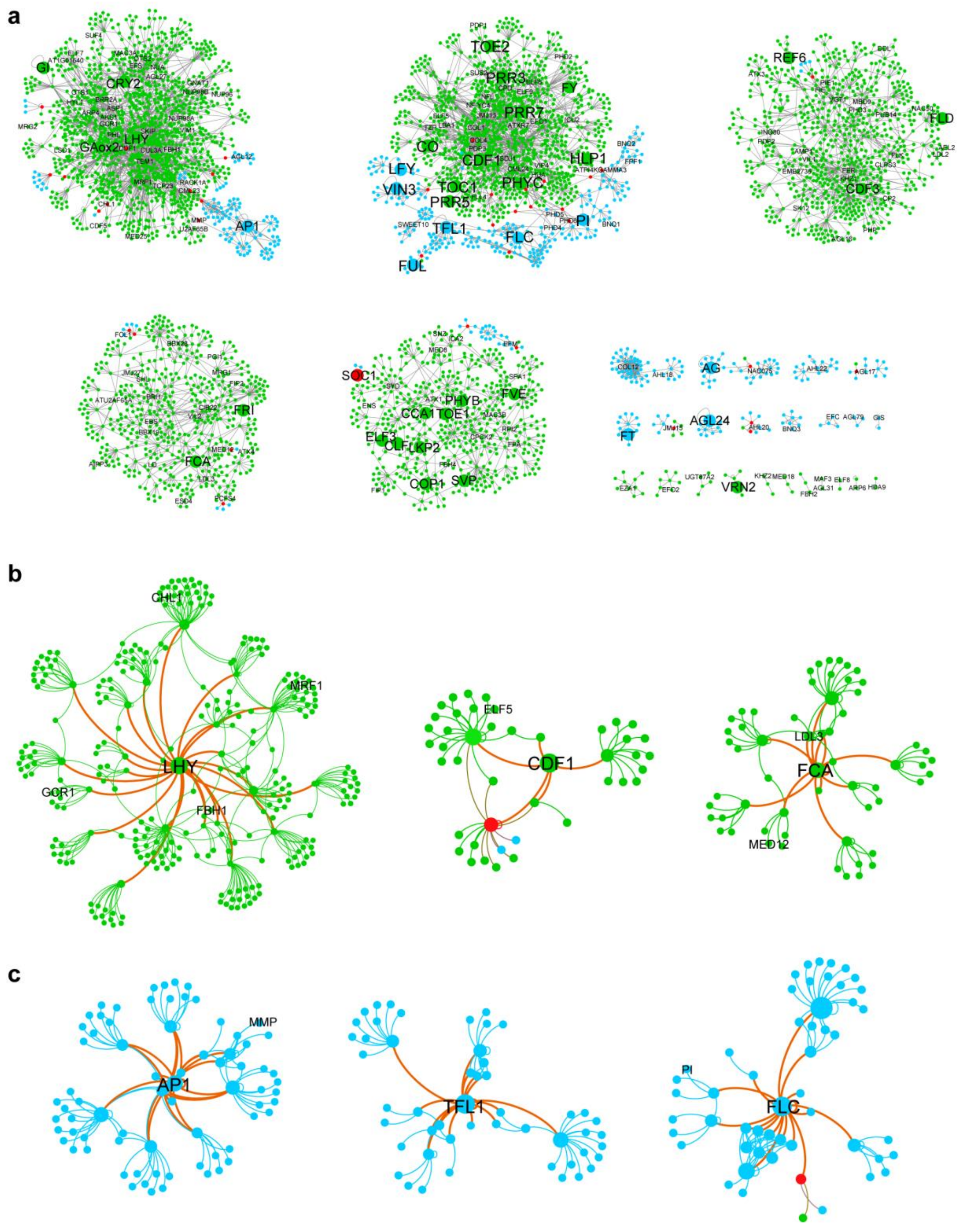

Fig S18. Connectivity networks converged by flowering-time control genes. **a** Flowering-time control gene-associated chromatin connectivity network during floral induction. The connectivity was built through two hops of all interactions mediated from 190 genes. Large circles represent the key flowering-time control genes. Small circles with gene symbol represent the node genes implicated in flowering-time control. Small circles without gene symbol represent the connecting genes. Colors represent their 3D network specificities.

b Representative of *LHY*-, *CDF1*-, and *FCA*-centric active chromatin connectivity network. Orange lines highlight *LHY*-, *CDF1*-, and *FCA*-associated interactions. **c** Representative of *AP1*-, *TFL1*-, and *FLC*-centric repressive chromatin connectivity network. Orange lines highlight *AP1*-, *TFL1*-, and *FLC*-associated interactions.

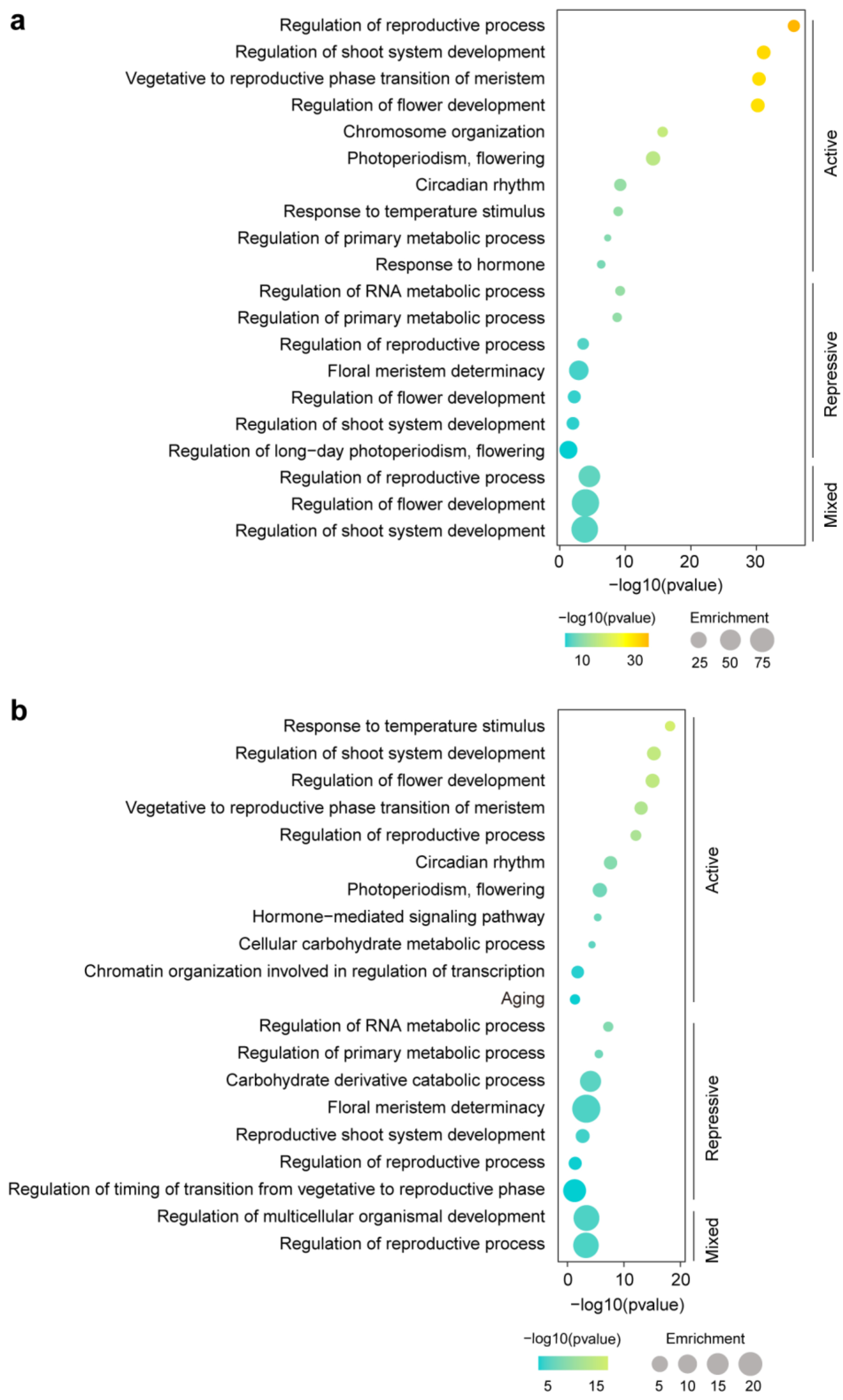

Fig S19. Enriched GO terms of genes involved in one hop (**a**) and two hops (**b**) of active mark-associated 3D network, repressive mark-associated 3D network, and mixed 3D network.
