# Supplementary material for: 3D organization of regulatory elements for transcriptional regulation in Arabidopsis
Source: Genome Biol. 2023 Aug 7;24:181. doi: 10.1186/s13059-023-03018-4 (PMC10405511; doi:10.1186/s13059-023-03018-4)
Supplement: Supplementary file 12 — Additional file 12. Review history. [file 13059_2023_3018_MOESM12_ESM.docx]

Review History

**First round of review**

**Reviewer 1**

**Were you able to assess all statistics in the manuscript, including the appropriateness of statistical tests used?**

Yes, and I have assessed the statistics in my report.

**Comments to author:**

In this manuscript, Deng et al. apply ChIA-PET method to detect Arabidopsis genome structure precisely, especially for long-range loops. Depending on these loops, the authors try to find the relationship between cis-element, modification, TF, SNP and gene expression. This study also mentioned a concept about gene hub, and suggested key roles that gene hubs play in the flowering control process. This observation may link gene locus on the chromatin to their biological function more tightly. In this study, there are many reasonable explanations are given based on data analysis, however, confirmatory experiments have not been done. Although the topic is interesting, the results in this manuscript are largely premature and insufficient to support the major conclusion.

Major point:

1. For the ChIA-PET results, I really wonder if there are something could be only detected by this technique but not Hi-C combining ChIP-seq in Arabidopsis? It should be discussed more about the advantages of ChIA-PET in Arabidopsis and compare with published Hi-C interactions.

2. Line 117-120, the author suggested Arabidopsis chromosomes have a Rabl-like configuration. However, more researchers believe rosette-like configuration fit Arabidopsis chromosomes. And in figure 1B, the ACA result just shows centromere clustering, but the model under the ACA implies telomere-to-centromere axis, while the interactions between telomeres are not shown in these results.

3. Line 182-183, for 43 different tissues, do they still have the same anchors as the aerial part of two-week-old seedlings? Which were used to define the loops and anchors? If long-range loops have function in regulating gene tissue specific expression, it is possible that loop anchor will be changed in different tissues and periods.

4. It is not surprising that H3K27me3 (Fig.2) has been widely reported to mediate long-range interactions. However, what is the molecular mechanism for RNAPII-associated long-range interactions? Are the genes at both ends of the interaction co-expressed? What are the similarities and differences between RNAPII-associated short-range and long-range interactions?

5. The authors identified so many long-range loops, it is necessary to verify they are really existed by 3D-FISH.

6. The authors should verify some of the loops in the corresponding TF mutants by 3C-PCR or 4C-seq.

Minor point：

1. Line 176-177, promoters and intergenic regions are important concepts for the author to classify the long-range loop. However, the authors do not give any statement about promoters and intergenic regions in this part. For example, how does the author define the length of the promoter?

2. In figure 3B, for the third column, should it be changed to "+ - + "(no anchor vs no basal)? The same question for 3C, does that mean part of genes have both basal and anchor modification?

3. Line 405-407, what is the co-expressed relationship if compared the genes have the same modification?

4. Line 443, "H3K9me3" may be wrong.

5. There are more than three types of activation marks, it is necessary to analyze more active marks and related interactions.

**Reviewer 2**

**Were you able to assess all statistics in the manuscript, including the appropriateness of statistical tests used?**

No, I do not feel adequately qualified to assess the statistics.

**Comments to author:**

In the manuscript entitled '3D Landscape of Regulatory Elements and Transcription Factors for Transcriptional Regulation in Arabidopsis', the authors used ChIA-PET of various histone modifications and Pol II to profile a fine-scale 3D chromatin map in Arabidopsis. They found some previously known chromatin structural feature in Arabidopsis. In addition, by integration of public available DHS and RNAseq data, they defined different types of CREs, and assigned the various types of long-range chromatin interactions. The authors also found several TFs binding sites are associated with chromatin loops and show some correlation with transcriptional level variation. Later, they take flowering regulator as example to show that several SNPs presence in the region near loop anchor or covered by loops. As well, the authors claim that flowering-time gene forming spatially distinct chromatin connective networks that coordinated with their expression regulation. In general, this work does provide a valuable data resource, but the authors' analysis of the data does not provide a deeper understanding of the how chromatin loop could contribute to transcription regulatory and eventually affects plant development.

Major concerns:

1. ChIA-PET is a technology that integrates HiC and ChIP-seq, in particular, it can provide information to link distal CREs to candidate target genes. In my opinion, such loop is dynamic with tissue specificity and developmental stage specificity. While the authors generated ChIA-PET data from the aerial part of two-week-old seedlings, other public available dataset are from different stages or tissue, e.g. DHS is from 7-day-old seedling, DNA methylation is from young shoots of two-week-old seedlings. How to incorporate those tissue or stage difference? Especially for the CRE part, since the DHS data is the main resource for defining CRE. The authors may consider to generate ChIA-PET dataset as well as DHS for different tissues or at different developmental stages to assay the dynamic of chromatin loops and better identify functional important CREs.

2. Similar as the above point, since the ChIA-PET dataset is from only one tissue of a particular developmental stage, how would the analysis of chromatin loop be informative to genes transcriptional regulation of various tissues and developmental stages, as RNAseq datasets are from multiple studies (Table S2).

3. About the coordination between chromatin interaction and TF occupancy. It is not surprised that there are higher TF binding sites in the loop anchor. But to conclude a 'TF-associated chromatin topology model for transcriptional regulation in Arabidopsis' as stated in lines 356-357, the authors need to do ChIA-PET and RNAseq in mutants of TFs.

4. In Figure7, authors connect flowering time-associated SNPs to flowering-time control genes such as FRIGIDA and FT using chromatin loop, even though those SNPs are close to loop anchor, but not right under the anchors. I think authors should test the effect of this SNPs to the loop formation, target genes expression and their influence on flowering through CRISPR-genome editing. Otherwise, the conclusion made on the functional importance of loop is not well supported. As well, I wonder how many functional verified SNPs are actually located within the loop anchors.

5. About the flowering time genes forming compartment of spatially distinct chromatin connective networks. Do the flowering activators or repressors have actually chromatin loop connection? If yes, how many of them are physically looped at 3D level? If no, please turn-down the conclusion.

Minor concerns:

1. In Figure2E, 2F, authors compared the transcription characters of anchor peaks and the basal peaks and found the difference. In line 180-182, authors found that the expression levels of active anchor genes tended to be higher than basal sites, and in Figure 2E, the expression level of H3K27me3 anchor genes tended to be lower than basal sites, which mean that the chromatin loop enhance the effect of chromatin markers on genes. How does the author think about this and what are the possible reasons behind it? Is there an additive effect on gene regulation because the chromatin loop increases the level of histone modification marks?

2. In line 170-174, authors compared the distribution pattern of chromatin loops between Arabidopsis and rice and found that the H3K4me3 and H3K9me2 mediated loop are different. I wonder if authors have thought about the underlying reasons for the differences and what are the consequences of it.

3. In line 282-285, I wonder if any of the loop captured in this manuscript have been verified in ref 48 and ref 49. I suggest the authors to do some verification test and show the ratio of loop captured by ChIA-PET vs experimentally verified.

4. The anchor peaks in ChIP-PAT data is only a subset of peaks of ChIP-seq data. As expected, characteristics such as effects on transcription and associations with CRE would not differ greatly between the two. So the novelty may need to pay on the comparison of those two peaks, and how unique is the histone modification peaks within anchors?

**Authors Response**

**Point-by-point responses to the reviewers’ comments:**

**Reviewer #1:**

In this manuscript, Deng et al. apply ChIA-PET method to detect Arabidopsis genome structure precisely, especially for long-range loops. Depending on these loops, the authors try to find the relationship between cis-element, modification, TF, SNP and gene expression. This study also mentioned a concept about gene hub, and suggested key roles that gene hubs play in the flowering control process. This observation may link gene locus on the chromatin to their biological function more tightly. In this study, there are many reasonable explanations are given based on data analysis, however, confirmatory experiments have not been done. Although the topic is interesting, the results in this manuscript are largely premature and insufficient to support the major conclusion.

Major point:

1. For the ChIA-PET results, I really wonder if there are something could be only detected by this technique but not Hi-C combining ChIP-seq in Arabidopsis? It should be discussed more about the advantages of ChIA-PET in Arabidopsis and compare with published Hi-C interactions.

***Response 1.1:*** *We thank the reviewer for the comments. Hi-C is typically employed to capture the 3D genome organization at large scale, such as compartments and topologically associating domains (TADs). However, due to its low resolution, it is insufficient to accurately identify chromatin loops between cis-regulatory elements (e.g., promoters and enhancers). In contrast, ChIA-PET can capture specific target protein-mediated chromatin interactions at nucleotide/binding-site resolution through proximity ligation and chromatin immunoprecipitation (ChIP). Thus, ChIA-PET can not only be utilized to reconstruct the genome’s comprehensive architecture, similar to the Hi-C approach, but also to identify finer-scale genome organization-chromatin loops at the nucleotide/binding-site level (Figures 1 and S4).*

*Unlike the observations that the genomes of mammals and many crops possess extensive TADs or TAD-like domains, TADs were not prominent in Arabidopsis. Thus, ChIA-PET is more appropriate than Hi-C to uncover previously obscured chromatin loops, and then establishes functional link between chromatin loops and gene regulation in Arabidopsis. In a side-by-side comparison of pooled ChIA-PET to the high-depth Hi-C data (Liu et al. 2016) in Arabidopsis, ChIA-PET recapitulated all the reported chromatin structures such as compartments and KNOTs with comparable data quality (Figures 1C–E and S3A). In addition, ChIA-PET captured extensive RNAPII- and histone modification-associated chromatin loops (Figure S3B), which allow us to investigate the features and functions of these chromatin loops in the downstream analysis.*

*Following the reviewer’s suggestions, we have added additional context to better interpret the advantages of using ChIA-PET in Arabidopsis, and also compared our ChIA-PET data with the published Hi-C data in the revised manuscript as follows:*

***Line 124-129:*** *In a side-by-side comparison of pooled ChIA-PET to the high-depth Hi-C data [62] in Arabidopsis, ChIA-PET recapitulated all the reported chromatin structures such as compartments and KNOTs with comparable data quality (Figures 1C–E and S3A). In addition, ChIA-PET captured extensive RNAPII- and histone modification-associated chromatin loops (Figure S3B), which allow us to investigate the features and functions of these chromatin loops in subsequent analysis.*

***Line 497-504:*** *Unlike chromosome conformation capture (3C)/Hi-C derivative approaches, ChIA-PET is a robust method for capturing specific target protein-associated chromatin interactions through proximity ligation and chromatin immunoprecipitation (ChIP). ChIA-PET can capture long-range chromatin loops between cis-regulatory elements, such as promoters and enhancers, at nucleotide/binding-site resolution. Since TADs or TAD-like domains were not prominent in Arabidopsis, ChIA-PET is more suitable than Hi-C to uncover previously obscured chromatin loops and thereby to establish functional connections between chromatin loops and gene regulation in Arabidopsis.*

Figure S3. Comparison of chromatin interaction patterns based on ChIA-PET and Hi-C data in *Arabidopsis*.

(A) Comparison of ChIA-PET data and Hi-C data from Liu et al. ChIA-PET recapitulates the primary chromatin structures as previously reported in Hi-C data. Pericentromeric regions are shown as heterochromatic compartments. Black dotted ovals indicate the *KNOT* nuclear structure. Genome-wide contact heatmaps in *Arabidopsis* at 50, 25, and 5 kb resolution. Elements represent normalized contact strength.

(B) Chromatin loops and binding peaks of the indicated factor in the box region of panel (A). The data tracks show chromatin loops and profiling of representative histone modifications and RNAPII occupancy.

2. Line 117-120, the author suggested Arabidopsis chromosomes have a Rabl-like configuration. However, more researchers believe rosette-like configuration fit Arabidopsis chromosomes. And in figure 1B, the ACA result just shows centromere clustering, but the model under the ACA implies telomere-to-centromere axis, while the interactions between telomeres are not shown in these results.

***Response 1.2:*** *We thank the reviewer for the comments. The cytological analyses suggest that Arabidopsis chromosomes adopt a rosette-like configuration, described by central chromocenters, from which euchromatic loops emanate (Fransz et al. 2002). Here, we suggested that Arabidopsis chromosomes adopt a Rabl-like configuration based on aggregate chromosome analysis (ACA) of the combined ChIA-PET data. Our result is similar to that obtained using Hi-C approach (Feng et al. 2014). Thus, the Arabidopsis chromosomal architecture revealed by cytological observation and 3D genome mapping approach are not exactly the same.*

*Although Sakamoto et al.’s study demonstrated that Arabidopsis is in a rosette-like state, strong interactions occur between different chromosome centromeres during the meiotic interval (Sakamoto et al. 2022) (Fig. 2c). We speculate that ChIA-PET or Hi-C data may capture the results of these interactions. Additionally, significant strong interactions between centromeres can be observed based on the Hi-C results in Sakamoto et al.’s study (2022) (Fig. S6). Thus, it is possible that the 3D genome architectures captured by Hi-C and ChIA-PET method may reflect ensemble structures from different cell types in Arabidopsis shoots or leaves, which including mesophyll, meristematic, epidermal, vasculature, and guard cells. To minimize the emphasis on this portion of the content, Figure 1B has been moved to the Supplementary Figures (Figure S2B), and the description of ACA result was also revised for better clarity as follows:*

***Line 132-137:*** *The ACA map exhibited prominent contacts within and between centromeres, and marginally frequent chromatin interactions* *both between the two telomeres of each single chromosome and among telomeres of different chromosomes (Figure S2B), which suggests that Arabidopsis chromosomes* *adopt a Rabl-like configuration,* *at least in some developmental stages or types of cells,* *similar to previous studies [23,64].*

Fig. 2c. Dynamics of centromeres visualized by *p35S::CENH3-tdTomato* during metaphase phase. Nuclei are visualized in green by *pRPS5a::H2B-GFP*. The optical section closest to the centre of the nucleus is shown. Scale bars, 5 μm. Adopted from Sakamoto et al. (2022).

Fig. S6. Visualization of Hi-C interaction frequency. Merged data were used. Red arrowheads indicate the chromatin interactions between centromeres/pericentromeres. Bin size = 50 kb. Adopted from Sakamoto et al. (2022).

3. Line 182-183, for 43 different tissues, do they still have the same anchors as the aerial part of two-week-old seedlings? Which were used to define the loops and anchors? If long-range loops have function in regulating gene tissue specific expression, it is possible that loop anchor will be changed in different tissues and periods.

***Response 1.3:*** *We agree with the reviewer’s comments. Both the linear epigenetic modifications and the 3D genome organization (including the anchors and chromatin loops) are dynamic in different tissues and periods, that is, the ‘4D Nucleome’. The 4D Nucleome Network aims to develop and apply approaches to map the structure and dynamics of the human and mouse genomes in space and time, with the goal of* *gaining deeper mechanistic insights into how the nucleus is organized and functions (Dekker et al. 2017). We propose that the long-term goal in plants should also be the same.*

*There may be some misunderstandings about the anchors used here. In this study, we focus on the organization and functions of Arabidopsis 3D genome in a representative tissue, aerial part of two-week-old seedlings. Basal and anchor peaks/genes were defined based on the seedling ChIA-PET data. Actually, the expression patterns (e.g., expression breadth and cooperative transcription) are properties of the basal and anchor genes, similar to the peak intensity and gene expression level. The transcriptome data from 43 different tissues are only used to characterize the expression patterns of the basal and anchor genes defined in seedlings. Our findings show that active anchor genes showed wider expression breadth than the active basal genes, while more Polycomb-repressive anchor genes were in tissue-specific categories compared to Polycomb-repressive basal genes (Figure 2F). Furthermore, most paired genes with active mark-associated chromatin interactions were highly expressed together (Figures 2G* *and S6C–E), and the mean Pearson correlation coefficient of active anchor gene pairs was significantly higher than that of randomly simulated gene pairs (Figure 2H), indicating that gene pairs involved in promoter-promoter interactions tended to be transcribed cooperatively.*

*To make it clearer, we have revised the following sentences in the updated manuscript as below:*

***Line 199-203:*** *We further adopted the transcriptome data from 43 different tissues (Table S3) to characterize the expression patterns (including expression breadth and cooperative transcription) of the basal and anchor genes defined in seedlings. We found that the active anchor genes showed wider expression breadth than the active basal genes (Figure 2F).*

4. It is not surprising that H3K27me3 (Fig.2) has been widely reported to mediate long-range interactions. However, what is the molecular mechanism for RNAPII-associated long-range interactions? Are the genes at both ends of the interaction co-expressed? What are the similarities and differences between RNAPII-associated short-range and long-range interactions?

***Response 1.4****: We appreciate the reviewer's comments. Investigating the molecular mechanism of RNAPII in 3D genome organization by using an RNAPII null mutant is almost impossible due to the essential role of RNAPII in plant survival. However, we can still explore the mechanism by examining the characteristics of RNAPII in 3D genome organization in wild-type plants.*

*In our previous research, we mapped the long-range chromatin interactions mediated by RNAPII in human cells (Li et al. 2012). Our results uncovered that promoter-centered chromatin interactions are widespread and serve as a topological foundation for regulating transcription (Li et al. 2012). Specifically, we observed that* *RNAPII-mediated chromatin interactions between promoters and distal regulatory elements occur within discrete foci of aggregated RNA polymerases in mammalian nuclei (Li et al. 2012). This suggests that active genes may be organized into transcription factories in 3D nuclear space to coordinate transcription.*

*In this study, we mapped the long-range chromatin interactions mediated by RNAPII and associated with distinct epigenetic states in Arabidopsis. Our results indicate that the vast majority (96%) of RNAPII-mediated chromatin interactions occur between promoters (Figure 2C), and the expression levels of RNAPII-associated paired anchor genes were highly correlated (Figures 2G and S6D). Furthermore, we found that RNAPII-, H3K4me3-, and H3K4me1-associated chromatin interactions shared the most anchors (Figure 3A) and reconstructed the active chromosome interacting domains (AIDs). Additionally, RNAPII and H3K4me3-associated spatial connectivity cooperatively facilitated transcription (Figures 3C and S11). In summary, our results suggest that RNAPII-tethered chromatin loops are involved in the co-expression of paired interacting genes. Active genes may be organized into transcription factories in 3D nuclear space to coordinate transcription. Additionally, our study sheds light on the interplay between active epigenetic marks (such as H3K4me3) and RNAPII-associated chromatin interactions in facilitating gene transcription.*

*As mentioned above, the anchor genes located at both ends of RNAPII-mediated chromatin loops, also known as paired anchor genes, exhibit a tendency to be highly expressed and co-expressed (Figures 2G and S6D). This suggests that RNAPII-mediated chromatin looping plays a role in the transcriptional regulation of the genes involved in chromatin interactions.*

*The next question concerns short-range and long-range chromatin interactions. In the present study, all chromatin interactions that we examined were long-range, as the distance between the paired anchors was greater than 3 kb. The long-range chromatin interactions were classified into three categories based on the loop span, including those smaller than 1 Mb, between 1 to 10 Mb, and larger than 10 Mb. No significant differences were found in GC content among the basal and* *all three categories of anchor sites (Fig. R1A). The peak intensity of anchors and the expression levels of anchor genes tended to be higher than those not involved in interactions (Fig. R1B and R1C). Anchors with loop span smaller than 1 Mb exhibited significantly lower peak intensity compared to those with loop span between 1 to 10 Mb or larger than 10 Mb (Fig. R1B).* *However, there was no significant difference in peak intensity between the latter two* *categories (Fig. R1B). The expression levels of anchor genes across three categories demonstrated a similar trend as their peak intensity (Fig. R1C).*

Fig. R1. Comparation of peaks/genes involved in RNAPII-mediated chromatin interactions with different loop spans.

(A-C) Boxplots for the GC content (A) and peak intensities (B) of RNAPII basal and anchor peaks involved in different chromatin loop spans, and the expression levels of RNAPII-occupied genes (C). ****p* < 0.001 from Wilcoxon test. NS, no significant difference.

5. The authors identified so many long-range loops, it is necessary to verify they are really existed by 3D-FISH.

***Response 1.5:*** *We appreciate the reviewer’s constructive suggestion. Following the reviewer’s suggestion, three chromatin interactions were validated by DNA fluorescence in situ hybridization. We have added the results, including figures (Figures 1B and S2A), table (Table S2), and methods in the revised manuscript.*

***Line 116-120:*** *To confirm the robustness of ChIA-PET and the existence of the chromatin interactions, three paired anchor loci involved in chromatin interactions were validated by DNA fluorescence in situ hybridization (FISH) in Arabidopsis seedling nuclei. As expected, DNA FISH confirmed a high frequency of interaction between the two anchor loci than randomly selected regions (Figures 1B and S2A, Table S2).*

Fig. 1B. Examples of DNA FISH-analyzed nuclei. Anchor regions are stained red and green, whereas DNA is stained blue. Bar = 2 µm.

Table S2. Chromatin interactions validated by DNA fluorescence in situ hybridization.

6. The authors should verify some of the loops in the corresponding TF mutants by 3C-PCR or 4C-seq.

***Response 1.6:*** *We appreciate the reviewer for the constructive comment. In this study, we used five published TF (ABI5, ATHB5, MYC2, CCA1, and SOC1) ChIP-seq data (Table S10) to investigate the relationship between TF-associated chromatin topology, TF occupancy and gene transcription in wild-type plants. Compared to 3C-PCR (one versus one) and 4C-seq (one versus many), ChIA-PET can capture genome-wide chromatin interactions (many versus many). Thus, we took TF mutant myc2 as an example and generated chromatin interactome (H3K4me3 ChIA-PET) data in myc2 and wild-type plants (Figure S16 and Table S5), which enabled us to investigate chromatin interactions at the whole genome level and elucidate the regulatory roles of MYC2 in 3D genome organization and gene transcription. In summary, we found that approximately one fifth of MYC2 ChIP-seq target gene-associated chromatin hubs underwent changes in myc2 compared to the wild-type, including disassembled (18%) or reorganized (1.5%) hubs. This result demonstrates that MYC2 may not impact all chromatin interactions associated with H3K4me3, but plays a critical role in shaping the spatial architecture of the Arabidopsis genome, particularly in relation to chromatin hubs associated with MYC2 targets. Additionally, our results reveal that MYC2 occupancy and its associated chromatin interactions may cooperatively facilitate gene transcription. Overall, our study uncovers the roles of TF, using MYC2 as an example, in regulating 3D genome architecture and gene transcription in plants.*

*These results have been added to the revised manuscript as follows:*

***Line 375-427:*** ***Roles of transcription factor MYC2 in regulating 3D genome architecture and gene transcription***

*To explore whether transcription factors, such as MYC2, may have a regulatory role in 3D genome organization and gene transcription, we generated chromatin interactome (H3K4me3 ChIA-PET) and transcriptome (RNA-seq) data using an improved method in myc2 mutant [70] and wild-type plants (Table S5). Considering the high reproducibility (> 0.95) of biological replicates for each ChIA-PET and RNA-seq dataset category (Figure S16 and Table S5), we combined the replicate data for downstream analysis.*

*We identified 32,312 H3K4me3-associated chromatin loops in myc2. Compared to the wild-type, approximately 19% H3K4me3-associated chromatin loops were lost in myc2, suggesting that MYC2 is* *partially responsible for the formation of chromatin loops. To investigate the impact of chromatin interaction and TF occupancy on gene expression, we evaluated the expression changes (myc2 vs. Col-0) of looping genes within those lost loops in myc2. Based on the MYC2 occupancy model, we categorized those lost loops into three categories (Figure 6D). As expected, the Log2 fold change of the looping genes with MYC2 binding were lower than those without MYC2 binding (Figure 6D). These results suggest that MYC2 occupancy and chromatin interaction play a crucial role in positively regulating gene transcription.*

*We next explored whether chromatin hubs mediated by MYC2 will be disassembled if the MYC2 activity is abolished. Of the total 7,971 H3K4me3-associated chromatin hubs detected in myc2 and wild-type, approximately 71% (5,626) contained MYC2 ChIP-seq target genes (Figure S17A). These MYC2 target-associated hubs were classified into four categories based on the change ratios of connecting genes between myc2 and wild-type: stable, entirely and partially disassembled, and others (Figures 6E and 6F). Of the 5,626 MYC2 target-associated hubs, 80% were stable hubs where most of their connecting genes were identical between wild-type and myc2 (e.g., CRB and RAN1 hubs); 17% were partially disassembled hubs in myc2* *compared to wild-type (e.g., ICE1 and GRP2 hubs); 1% were entirely disassembled hubs where most of their connecting genes were disassembled in myc2 (e.g., MBD10 and BAK1 hubs). The remaining hubs (1.5%, others) either were formed newly or exhibited significant changes in size in myc2 (Figures 6E and 6F). These results suggest that MYC2 may not impact all chromatin interactions associated with H3K4me3 but plays a crucial role in determining the spatial architecture of the Arabidopsis genome, particularly in relation to chromatin hubs associated with MYC2 targets (Figures 6E and S17B).*

*Furthermore, we classified the genes within the disassembled hubs into two categories: genes that retained chromatin interaction in myc2 compared to the wild-type, referred to as aggregated genes, and genes that lost chromatin interaction in myc2, referred to as separated genes. Notably, the expression of the separated genes was significantly inhibited in myc2 compared to the aggregated genes (Figure S17C). This observation suggests a positive correlation between chromatin interactions and gene transcription within the high-order 3D genome organization, specifically in chromatin hubs. For instance, AtPUB19, a U-Box E3 ubiquitin ligase involved in regulating abscisic acid and drought responses [71], was bound by MYC2 and served as the node gene within the* *AtPUB19-centric hub in wild-type (Figure 6H). The absence of MYC2 led to* *reduction of* *chromatin connectivity within the AtPUB19-associated hub and also* *downregulation of AtPUB19 gene expression (Figure 6H). These results suggest that TFs may create a conducive microenvironment for gene transcription by reorganizing the spatial genome organization, particularly in chromatin hubs.*

*Collectively, our results demonstrate the regulatory roles of TFs, exemplified by MYC2, in governing 3D genome organization and gene transcription (Figure 6I). We propose that MYC2, in conjunction with other TFs and mediators, orchestrates the formation of specialized microenvironments called chromatin* *hubs, which actively facilitate gene transcription in the wild-type. Notably, MYC2 is necessary for a subset of these chromatin hubs. Therefore, the removal of MYC2 disrupts this microenvironment, leading to the disassembly of MYC-centric hubs and a subsequent downregulation of gene transcription.*

**Figure 6.** **TF-associated chromatin topology and its transcriptional function.**

(D) Log2 fold change in expression of looping genes associated with lost loops in *myc2* mutant compared to the wild-type. Fold-changes represent the expression changes of each gene in *myc2* mutant relative to the wild-type. The lost loops were divided into three categories based on the MYC2 occupancy model. **p* < 0.05 from Wilcoxon test.

(E) Changes of MYC2 target-associated chromatin hubs in *myc2* mutant compared to wild-type. These hubs were classified into four categories: stable, entirely and partially disassembled, and others. The number and proportion of hubs included in each category were given.

(F) Representative examples of stable, partially, and entirely disassembled hubs in *myc2* and wild-type.

(G) Percentages of shared connecting genes in the indicated hub categories between *myc2* and wild-type. ****p* < 0.001 from Wilcoxon test.

(H) Browser screenshot showing the chromatin connectivity and gene transcription within AtPUB19-centric hub. Light orange box indicates reduction of chromatin connectivity within the AtPUB19-centric hub and downregulation of *AtPUB19* gene expression. Each MYC2 binding site, chromatin interaction, H3K4me3 peak, and RNA-seq track represent the normalized loops and read coverage in *myc2* and wild-type.

(I) A proposed model for the impacts of MYC2 in 3D genome organization and gene transcription. In the MYC2 dominated chromatin hub, MYC2 coupled with other TFs and mediators to create microenvironment that facilitates gene transcription in the wild-type. As MYC2 activity is abolished, the MYC2-centric hub is disassembled and the gene expression decreases. More red wavy lines indicate higher expression.

Minor point:

1. Line 176-177, promoters and intergenic regions are important concepts for the author to classify the long-range loop. However, the authors do not give any statement about promoters and intergenic regions in this part. For example, how does the author define the length of the promoter?

***Response 1.7:*** *Following the reviewer’s suggestion, we define promoters and intergenic regions in the revised manuscript.*

***Line 1091-1093:*** *The regions of 1 kb upstream to 0.5 kb downstream of the transcription start site (TSS) of annotated protein-coding genes were defined as promoters. Intergenic regions referred to the regions excluding 1 kb upstream of the TSS to the transcription termination site.*

2. In figure 3B, for the third column, should it be changed to "+ - + "(no anchor vs no basal)? The same question for 3C, does that mean part of genes have both basal and anchor modification?

***Response 1.8:*** *Thank you for the comment. We apologize for the mistake and have revised figures 3B, 3C, and S11 in the revised manuscript as suggested.*

3. Line 405-407, what is the co-expressed relationship if compared the genes have the same modification?

***Response 1.9:*** *We thank the reviewer for the comment. Based on our existing analysis, we have analyzed the co-expression of genes that do not control flowering time but have the same modification. Our results indicate that the subnetworks only associated with flowering time genes exhibit higher co-expression than those associated with all interacting genes or non-flowering time genes (Fig. R2). These results suggest that genes controlling flowering time are more likely to be organized together to coordinate transcription within the 3D nuclear space, as compared to other mixed gene pathways.*

Fig. R2. Co-expression analysis of flowering time and non-flowering time genes-associated subnetworks. The PCC of subnetworks were calculated. Random, randomly selected gene pairs with the same physical distance. Total, all representative chromatin mark-associated anchor gene pairs. ***p* < 0.01, **p* < 0.05 from Wilcoxon test. NS, no significant difference.

4. Line 443, "H3K9me3" may be wrong.

***Response 1.10:*** *Thank you for pointing out the mistake. The sentence has been revised as "Heterochromatin regions form liquid-like foci mediated by H3K9me2/3 and its readers [79,80], acetylated chromatin form multivalent interactions with multi-bromodomain proteins and facilitate the phase separation of chromatin [81], and the transcriptional machinery at genomic loci are formed by phase-separated proteins that contain intrinsically disordered domains, such as RNAPII, TFs, and coactivators [82,83]* *in mammal cells" (line 517-522).*

5. There are more than three types of activation marks, it is necessary to analyze more active marks and related interactions.

***Response 1.11:*** *Thank you for the comment. Yes, there are several types of active histone modifications, such as H3K4me1, H3K4me3, H3K9ac, H3K27ac, H3K9me3, and H3K36me3, which play important roles in regulating gene expression in plants (Berr et al. 2011). We evaluated H3K9ac and H3K27ac ChIP-seq data, and found that their peak profiles are highly consistent with the H3K4me3 pattern (Fig. R3). In the previous studies, we demonstrated that these active markers change synchronously in the DNA methylation-free mutants when investigating the role of DNA methylation in regulating histone modifications (Zhao et al. 2022). These results suggest that these active marks may perform similar functions in gene transcription. Moreover, the genome distribution of other typical active markers tends to be enriched in promoters or gene bodies, which is similar to the active markers used in this study (Xiao et al. 2016). Therefore, we selected representative marks with distinct genome distributions and transcriptional functions for further analysis, which include H3K4me3, H3K4me1, H3K27me3, H3K9me2, and RNAPII.*

Fig. R3. Genome-wide distribution of H3K4me3, H3K9ac, and H3K27ac in the aerial part of two-week-old seedlings.

(A) Genome-wide profile of H3K4me3, H3K9ac, and H3K27ac. Regions shown are ± 2 kb from H3K4me3 peak summits.

(B) Features of H3K4me3, H3K9ac, H3K27ac, and gene expression at the indicated genome region. Each H3K4me3, H3K9ac, H3K27ac, and RNA-seq track represents the normalized read coverage (wiggle plot).

**Reviewer #2:**

In the manuscript entitled '3D Landscape of Regulatory Elements and Transcription Factors for Transcriptional Regulation in Arabidopsis', the authors used ChIA-PET of various histone modifications and Pol II to profile a fine-scale 3D chromatin map in Arabidopsis. They found some previously known chromatin structural feature in Arabidopsis. In addition, by integration of public available DHS and RNAseq data, they defined different types of CREs, and assigned the various types of long-range chromatin interactions. The authors also found several TFs binding sites are associated with chromatin loops and show some correlation with transcriptional level variation. Later, they take flowering regulator as example to show that several SNPs presence in the region near loop anchor or covered by loops. As well, the authors claim that flowering-time gene forming spatially distinct chromatin connective networks that coordinated with their expression regulation. In general, this work does provide a valuable data resource, but the authors' analysis of the data does not provide a deeper understanding of the how chromatin loop could contribute to transcription regulatory and eventually affects plant development.

Major concerns:

1. ChIA-PET is a technology that integrates Hi-C and ChIP-seq, in particular, it can provide information to link distal CREs to candidate target genes. In my opinion, such loop is dynamic with tissue specificity and developmental stage specificity. While the authors generated ChIA-PET data from the aerial part of two-week-old seedlings, other public available dataset are from different stages or tissue, e.g. DHS is from 7-day-old seedling, DNA methylation is from young shoots of two-week-old seedlings. How to incorporate those tissue or stage difference? Especially for the CRE part, since the DHS data is the main resource for defining CRE. The authors may consider to generate ChIA-PET dataset as well as DHS for different tissues or at different developmental stages to assay the dynamic of chromatin loops and better identify functional important CREs.

***Response 2.1:*** *We appreciate the reviewer’s constructive comments. Following the reviewer’s suggestions, we have replaced the DNase-seq data from 7-day-old seedlings with the ATAC-seq (assay for transposase-**accessible chromatin using sequencing) data from the aerial part of two-week-old seedlings to define the accessible chromatin regions. The scatter plot and genome browser screenshot reveal the high quality and reproducibility between the ATAC-seq biological replicates generated in this study* *(Figures S13A and S13B). The accessible chromatin map produced by ATAC-seq is equivalent to that of DNase-seq (Fig. R4), and thus, the conclusions drawn from the ATAC-seq data align with those of the DNase-seq data. We have revised all ATAC-seq-related figures and conclusions in the revised manuscript (Figures 4, 5, S13, and S14). In addition, the DNA methylation data and ChIA-PET data used in this study were both generated in* *our laboratory and obtained from the aerial part of two-week-old seedlings. Therefore, these data used in this study can be compared to each other.*

*The aerial part of two-week-old seedlings is chosen as a representative tissue to explore the Arabidopsis 3D genome architecture. Our future research will focus on exploring the dynamics and functions of chromatin accessibility and 3D genome architecture in different tissues and developmental stages.*

Fig. S13A. Pearson correlation for two ATAC-seq biological replicates. The R value calculated by Pearson correlation coefficient at 10 kb genomic bin is shown.

Fig. S13B. Browser screenshot showing the chromatin accessibility in the indicated region between two ATAC-seq replicates.

Fig. R4. Comparation of accessible chromatin regions defined by ATAC-seq (this study) and DNase-seq (Sullivan et al. 2014).

(A) Correlation of peak intensities between ATAC-seq and DNase-seq. Pearson correlation coefficient at 10 kb genomic bin is shown.

(B) A Venn diagram showing the overlap of accessible regions between ATAC-seq and DNase-seq.

2. Similar as the above point, since the ChIA-PET dataset is from only one tissue of a particular developmental stage, how would the analysis of chromatin loop be informative to genes transcriptional regulation of various tissues and developmental stages, as RNA-seq datasets are from multiple studies (Table S2).

***Response 2.2:*** *We thank the reviewer for the comments. Actually, both the linear epigenetic modifications and the 3D genome organization, including the anchors and chromatin loops, exhibit dynamic changes across different tissues and developmental stages, that is, the ‘4D Nucleome’. The 4D Nucleome Network aims to develop and apply approaches to map the structure and dynamics of the human and mouse genomes in space and time with the goal of gaining deeper mechanistic insights into how the nucleus is organized and how it functions (Dekker et al. 2017). This is also the goal of our plant 3D genome research in the future.*

*There may be some misunderstandings regarding the anchors used in this study. Here, we first defined the anchor and basal genes based on the ChIA-PET data obtained from the aerial part of two-week-old seedlings, which serves as a representative plant tissue. Next, we characterized the expression patterns (e.g., expression breadth and cooperative transcription) of the anchor and basal genes defined in seedlings. Of note, the expression patterns, similar to the peak intensity and gene expression level, are the properties of basal and anchor genes defined in seedlings. The transcriptome data from 43 different tissues are only used to characterize the expression patterns of the basal and anchor genes defined in seedlings. Our findings revealed that active anchor genes showed wider expression breadth than the active basal genes (Figure 2F). However, more Polycomb-repressive anchor genes were in tissue-specific categories compared to Polycomb-repressive basal genes (Figure 2F). Furthermore, most paired genes with active mark-associated chromatin interactions were highly expressed together (Figures 2G and S6C–E), and the mean Pearson correlation coefficient of active anchor gene pairs was significantly higher than that of randomly simulated gene pairs (Figure 2H). These results indicate that paired genes involved in chromatin interactions are more likely to be transcriptionally coordinated. Overall, we utilized RNA-seq data derived from 43 different tissues to reveal the characteristics and potential regulatory function of chromatin loops defined in one representative plant tissue.*

*To make it clearer,* *we have added the following sentence in the revised manuscript as below:*

***Line 199-203:*** *We further adopted the transcriptome data from 43 different tissues (Table S3) to characterize the expression patterns (including expression breadth and cooperative transcription) of the* *basal and anchor genes defined in seedlings. We found that the active anchor genes showed wider expression breadth than the active basal genes (Figure 2F).*

3. About the coordination between chromatin interaction and TF occupancy. It is not surprised that there are higher TF binding sites in the loop anchor. But to conclude a 'TF-associated chromatin topology model for transcriptional regulation in Arabidopsis' as stated in lines 356-357, the authors need to do ChIA-PET and RNA-seq in mutants of TFs.

***Response 2.3:*** *We* *thank the reviewer for the constructive suggestion. Following the reviewer’s suggestion, we have generated H3K4me3 ChIA-PET and RNA-seq data in TF mutant myc2 (Figure S16 and Table S5), which enabled us to investigate chromatin interactions at the whole genome level and elucidate the regulatory functions of MYC2 in 3D genome organization and gene transcription. In summary, we found that approximately one fifth of MYC2 target gene-associated chromatin hubs underwent changes compared to the wild-type, including disassembly (18%) or reorganization (1.5%). This result demonstrates that although MYC2 does not impact all chromatin interactions associated with H3K4me3, it plays a critical role in shaping the spatial architecture of the Arabidopsis genome, particularly in relation to chromatin hubs associated with MYC2 targets. Additionally, our results reveal that MYC2 occupancy and its associated chromatin interactions may cooperatively facilitate gene transcription. Overall, our study uncovers the roles of TF, using MYC2 as an example, in regulating 3D genome architecture and gene transcription in plants.*

*These results have been added to the revised manuscript as follows:*

***Line 375-427:*** ***Roles of transcription factor MYC2 in regulating 3D genome architecture and gene transcription***

*To explore whether transcription factors, such as MYC2, may have a regulatory role in 3D genome organization and gene transcription, we generated chromatin interactome (H3K4me3 ChIA-PET) and transcriptome (RNA-seq) data using an improved method in myc2 mutant [70] and wild-type plants (Table S5). Considering the high reproducibility (> 0.95) of biological replicates for each ChIA-PET and RNA-seq dataset category (Figure S16 and Table S5), we combined the replicate data for downstream analysis.*

*We identified 32,312 H3K4me3-associated chromatin loops in myc2. Compared to the wild-type, approximately 19% H3K4me3-associated chromatin loops were lost in myc2, suggesting that MYC2 is partially responsible for the formation of chromatin loops. To investigate the impact of chromatin interaction and TF occupancy on gene expression, we evaluated the expression changes (myc2 vs. Col-0)* *of looping genes within those lost loops in myc2. Based on the MYC2 occupancy model, we categorized those lost loops into three categories (Figure 6D). As expected, the Log2 fold change of the looping genes with MYC2 binding were lower than those without MYC2 binding (Figure 6D). These results suggest that MYC2 occupancy and chromatin interaction play a crucial role in positively regulating gene transcription.*

*We next explored whether chromatin hubs mediated by MYC2 will be disassembled if the MYC2 activity is abolished. Of the total 7,971 H3K4me3-associated chromatin hubs detected in myc2 and wild-type, approximately 71% (5,626) contained MYC2 ChIP-seq target genes (Figure S17A). These MYC2 target-associated hubs were classified into four categories based on the change ratios of connecting genes between myc2 and wild-type: stable, entirely and partially disassembled, and others (Figures 6E and 6F). Of the 5,626 MYC2 target-associated hubs, 80% were stable hubs where most of their connecting genes were identical between wild-type and myc2 (e.g., CRB and RAN1 hubs); 17% were partially disassembled hubs in myc2 compared to wild-type (e.g., ICE1 and GRP2 hubs); 1% were entirely disassembled hubs where most of their connecting genes were disassembled in myc2 (e.g., MBD10 and BAK1 hubs). The remaining hubs (1.5%, others) either were formed newly or exhibited significant changes in size in myc2 (Figures 6E and 6F). These results suggest that MYC2 may not impact all chromatin interactions associated with H3K4me3 but plays a crucial role in determining the spatial architecture of the Arabidopsis genome, particularly in relation to chromatin hubs associated with MYC2 targets (Figures 6E and S17B).*

*Furthermore, we classified the genes within the disassembled hubs into two categories: genes that retained chromatin interaction in myc2 compared to the wild-type, referred to as aggregated genes, and genes that lost chromatin interaction in myc2, referred to as separated genes. Notably, the expression of the separated genes was significantly inhibited in myc2 compared to the aggregated genes (Figure S17C). This observation suggests a positive correlation between chromatin interactions and gene transcription within the high-order 3D genome organization, specifically in chromatin hubs. For instance, AtPUB19, a U-Box E3 ubiquitin ligase involved in regulating abscisic acid and drought responses [71], was bound by MYC2 and served as the node gene within the AtPUB19-centric hub in wild-type (Figure 6H). The absence of MYC2 led to reduction of chromatin connectivity within the AtPUB19-associated hub and also downregulation of AtPUB19 gene expression (Figure 6H). These results suggest that TFs may create a conducive microenvironment for gene transcription by reorganizing the spatial genome organization, particularly in chromatin hubs.*

*Collectively, our results demonstrate the regulatory roles of TFs, exemplified by MYC2, in governing 3D genome organization and gene transcription (Figure 6I). We propose that MYC2, in conjunction with other TFs and mediators, orchestrates the formation of specialized microenvironments called chromatin hubs, which actively facilitate gene transcription in the wild-type. Notably, MYC2 is necessary for a subset* *of these chromatin hubs. Therefore, the removal of MYC2 disrupts this microenvironment, leading to the disassembly of MYC-centric hubs and a subsequent downregulation of gene transcription.*

**Figure 6. TF-associated chromatin topology and its transcriptional function.**

(D) Log2 fold change in expression of looping genes associated with lost loops in *myc2* mutant compared to the wild-type. Fold-changes represent the expression changes of each gene in *myc2* mutant relative to the wild-type. The lost loops were divided into three categories based on the MYC2 occupancy model. **p* < 0.05 from Wilcoxon test.

(E) Changes of MYC2 target-associated chromatin hubs in *myc2* mutant compared to wild-type. These hubs were classified into four categories: stable, entirely and partially disassembled, and others. The number and proportion of hubs included in each category were given.

(F) Representative examples of stable, partially, and entirely disassembled hubs in *myc2* and wild-type.

(G) Percentages of shared connecting genes in the indicated hub categories between *myc2* and wild-type. ****p* < 0.001 from Wilcoxon test.

(H) Browser screenshot showing the chromatin connectivity and gene transcription within AtPUB19-centric hub. Light orange box indicates reduction of chromatin connectivity within the AtPUB19-centric hub and downregulation of *AtPUB19* gene expression. Each MYC2 binding site, chromatin interaction, H3K4me3 peak, and RNA-seq track represent the normalized loops and read coverage in *myc2* and wild-type.

(I) A proposed model for the impacts of MYC2 in 3D genome organization and gene transcription. In the MYC2 dominated chromatin hub, MYC2 coupled with other TFs and mediators to create microenvironment that facilitates gene transcription in the wild-type. As MYC2 activity is abolished, the MYC2-centric hub is disassembled and the gene expression decreases. More red wavy lines indicate higher expression.

4. In Figure7, authors connect flowering time-associated SNPs to flowering-time control genes such as FRIGIDA and FT using chromatin loop, even though those SNPs are close to loop anchor, but not right under the anchors. I think authors should test the effect of this SNPs to the loop formation, target genes expression and their influence on flowering through CRISPR-genome editing. Otherwise, the conclusion made on the functional importance of loop is not well supported. As well, I wonder how many functional verified SNPs are actually located within the loop anchors.

***Response 2.4:*** *We thank the reviewer for the constructive comments. The definition of anchor is as follows: in the genome browser visualization, and conceptually speaking, anchor is the genomic region covered by the specific protein factor-binding peaks involved in chromatin interactions. In the data analysis, each inter-ligation PET was derived from two ChIP DNA fragments. We provide the specific protein factor-binding peaks to represent the virtual DNA fragments in pairs. These peaks are considered equivalent to “anchors” (Li et al. 2012). We apologize for the confusion that “SNPs are close to loop anchor, but not right under the anchors” caused by our unclear presentation in Figure 7. We have revised Figures 7B and 7C with SNP-located light-green box covering the associated anchor peak, which reveals SNPs are within the anchor regions.*

*In this study, we utilized a combination of the genome-wide association studies (GWAS)-defined phenotype-associated significant single nucleotide polymorphisms (SNPs) and ChIA-PET data. Through* *this approach, we discovered long-range chromatin interactions that connect distal elements, where the SNPs are located, to target genes. These findings provide possible mechanistic explanations for the role of these elements in agronomic traits. We propose that SNPs potentially influence phenotypic variation by affecting target genes through chromatin interactions, thus highlighting the connection between genotype, spatial chromatin interaction, and phenotype. As suggested in our manuscript and by the reviewer, further experimental validation is necessary to confirm this functional chromatin interaction model, such as conducting transgenic or genome editing assays. However, introducing point mutations at SNP sites using genome editing tools like CRISPR is challenging and time-consuming. This work should be the primary focus of a separate project. To the best of our knowledge, no reports exist regarding the functional validation of the functional chromatin interaction model (functional SNP-chromatin loop-phenotype) through genetic editing. Nonetheless, our research has identified numerous potential sites for spatial functional interactions, which warrant the initiation of new projects for genetic verification in future follow-up studies.*

5. About the flowering time genes forming compartment of spatially distinct chromatin connective networks. Do the flowering activators or repressors have actually chromatin loop connection? If yes, how many of them are physically looped at 3D level? If no, please turn-down the conclusion.

***Response 2.5:*** *We thank the reviewer for the comments. Approximately 241 flowering time genes were collected (Table S7), and 119 flowering activators and 122 flowering repressors were identified based on the flowering phenotype. Of the 119 flowering activators, 98 genes were integrated to the chromatin connectivity maps; Of the 122 flowering repressors, 92 genes were integrated to the chromatin connectivity maps. We next constructed one-hop chromatin connectivity networks using active mark- and H3K27me3-associated interacting gene pairs originating from flowering activators or repressors, respectively. Our results show that both the flowering activators and repressors are implicated in both the active- and H3K27me3-associated connectivity networks (Fig. R5). When the network analysis was extended from one to two hops of connectivity, the flowering-time control genes were found to be connected within five major subnetworks, except for a small fraction of sporadic interactions (Fig. R6).*

*As presented in Figures 7D and S18, most of the expressed (active) and repressed flowering time genes are involved in the corresponding active- and H3K27me3-associated connectivity networks, respectively, in line with the notion that the chromatin conformation coordinates the initiation of the floral induction programs and repression of inflorescence meristem identification programs to properly control vegetative to reproductive phase switches. These results also indicate that 3D genome organization could accurately* *reflect the chromatin states and expression levels of genes, which match well with the* *spatio-temporal activity of functionally related genes. Thus, it is plausible here to conduct analysis based on the higher-order genome architecture-related transcriptional activity rather than the flowering phenotype function, which is controlled by not only the epigenetic/3D landscape but also mixed gene regulatory networks.*

Fig. R5. Flowering activator- or repressor-associated chromatin connectivity network during floral induction. The connectivity was built through one-hop of all interactions mediated from 98 flowering activators or 92 flowering repressors. Colors represent their 3D network specificities.

Fig. R6. Flowering activator- or repressor-associated chromatin connectivity network during floral induction. The connectivity was built through two-hop of all interactions mediated from 98 flowering activators or 92 flowering repressors. Colors represent their 3D network specificities.

Minor concerns:

1. In Figure2E, 2F, authors compared the transcription characters of anchor peaks and the basal peaks and found the difference. In line 180-182, authors found that the expression levels of active anchor genes tended to be higher than basal sites, and in Figure 2E, the expression level of H3K27me3 anchor genes tended to be lower than basal sites, which mean that the chromatin loop enhance the effect of chromatin markers on genes. How does the author think about this and what are the possible reasons behind it? Is there an additive effect on gene regulation because the chromatin loop increases the level of histone modification marks?

***Response 2.6:*** *We thank the reviewer for the comments. It is possible that chromatin loop has additive effect on gene transcription via increasing the peak intensity of histone modification marks, since the anchor peaks have higher peak intensity than the basal peaks.*

*Indeed, a central debate in the 3D genome field revolves around the causal relationships between the 3D chromatin architecture, chromatin states, and transcription. The presence of a specific chromatin structure is not sufficient to initiate gene transcription. For instance, tethering a gene locus to the nuclear lamina does not necessarily induce gene repression (van Steensel and Belmont 2017) and promoter–promoter interactions can occur without subsequent gene activation (Jin et al. 2013; Ghavi-Helm et al. 2014). However, experimental manipulation of 3D genome architecture has shown that inducing promoter–enhancer proximity or disrupting TAD boundary results in altered gene expression (Deng et al. 2012; Lupianez et al. 2015). On the other hand, inhibition of transcription does not disrupt existing genome conformation (Mitchell and Fraser 2008; Palstra et al. 2008). Therefore, it appears that function (transcription) frequently follows structure (genome architecture).*

*The interplay between chromatin interaction and chromatin states (e.g., histone modifications) is complex and interdependent. Each of the biochemical activities of histone modifications (e.g., opening chromatin and recruiting cofactors), may affect 3D genome organization. For instance, histone modifications were sufficient to induce A–B compartment switching (Therizols et al. 2014; Wijchers et al. 2016; van Steensel and Belmont 2017). Conversely, chromosome conformation affects histone modification function and the pairing of histone modification-associated DNA elements (Kim and Shendure 2019).*

*In summary, there is a constant interplay between chromatin interaction, chromatin states, and transcription. Spatial genome (re)organization does not strictly control transcription, but alterations of genome conformation can create a permissive substrate for subsequent transcriptional changes. Thus, genome folding plays a significant role in shaping gene expression programs by providing instructive cues.*

2. In line 170-174, authors compared the distribution pattern of chromatin loops between Arabidopsis and rice and found that the H3K4me3 and H3K9me2 mediated loop are different. I wonder if authors have thought about the underlying reasons for the differences and what are the consequences of it.

***Response 2.7:*** *We thank the reviewer for the comments. The distinct distribution patterns of chromatin loops associated with H3K4me3 and H3K9me2 in Arabidopsis and rice may be attributed to the differences in the distribution and content of transposons/repeat elements and active genes. The differences in genome composition led to significant variations in chromatin states and affect 3D genome organization across different plant species. Consistent with these differences, H3K9me2-associated chromatin interaction domains (HIDs) were exclusively enriched in centromeric and pericentromeric regions in Arabidopsis, whereas approximately 20% of HIDs were located in euchromatin in rice (Figure S9). The loop span of active gene-related H3K4me3 chromatin interactions is greater in Arabidopsis than in rice (Figure S7) (Zhao et al. 2019). This could also be a reason why TADs were not observed in Arabidopsis, but were present in rice. Thus, the organization of the genome reflects functional compartmentalization, with different types of chromatin interaction domains exhibiting varying transcriptional activities based on their distinct epigenetic and spatial features.*

3. In line 282-285, I wonder if any of the loop captured in this manuscript have been verified in ref 48 and ref 49. I suggest the authors to do some verification test and show the ratio of loop captured by ChIA-PET vs experimentally verified.

***Response 2.8:*** *We thank the reviewer for the suggestion. In ref 48 and ref 49 (now ref 47 and ref 48), the authors verified the predicted enhancers using a β-glucuronidase (GUS) reporter assay rather than 3D genome mapping methods. This study aims to validate the predicted enhancers using ChIA-PET approach. In total, we found 3,783 active DREs as enhancer candidates, which play significant roles in the interacting genes through long-range chromatin loops. Furthermore, several chromatin interactions were validated by DNA fluorescence in situ hybridization (Figures 1B and S2A, Table S2).*

4. The anchor peaks in ChIP-PAT data is only a subset of peaks of ChIP-seq data. As expected, characteristics such as effects on transcription and associations with CRE would not differ greatly between the two. So the novelty may need to pay on the comparison of those two peaks, and how unique is the histone modification peaks within anchors?

***Response 2.9:*** *We thank the reviewer for the comments. To explore the relationship between anchor and basal peaks, we compared the peak intensity, peak length, and GC content of basal and anchor peaks, with anchor peak involved in loop with span smaller than 1 Mb, between 1 to 10 Mb, and larger than 10 Mb. No significant differences in GC content were observed among the basal and all three categories of anchor peaks (Fig. R7A). The peak intensity of anchor sites tended to be higher than those not involved in interactions (Basal) (Fig. R7B). In addition, the peak intensity of anchor sites with loop span smaller than 1 Mb were significantly lower than those loop span between 1 to 10 Mb and larger than 10 Mb. However, there was no significant difference in peak intensity between the latter two categories (Fig. R7B). The peak length of anchor sites and the expression levels of anchor genes across three categories demonstrated a similar trend as their peak intensity (Fig. R7C and R7D).*

Fig. R7. Comparation of the indicated factor-associated peaks/genes involved in chromatin interaction with different loop spans.

(A-D) Boxplots for the peak intensity (A), GC content (B), and peak length (C) of the indicated factor-associated basal and anchor peaks involved in different chromatin loop spans, and the expression levels of the indicated factor-occupied genes (D). ****p* < 0.001 from Wilcoxon test. NS, no significant difference.

***References***

*Berr A, Shafiq S, Shen WH. 2011. Histone modifications in transcriptional activation during plant development. Biochim Biophys Acta* ***1809****: 567-576.*

*Dekker J, Belmont AS, Guttman M, Leshyk VO, Lis JT, Lomvardas S, Mirny LA, O'Shea CC, Park PJ, Ren B et al. 2017. The 4D nucleome project. Nature* ***549****: 219-226.*

*Deng W, Lee J, Wang H, Miller J, Reik A, Gregory PD, Dean A, Blobel GA. 2012. Controlling long-range genomic interactions at a native locus by targeted tethering of a looping factor. Cell* ***149****: 1233-1244.*

*Feng S, Cokus SJ, Schubert V, Zhai J, Pellegrini M, Jacobsen SE. 2014. Genome-wide Hi-C analyses in wild-type and mutants reveal high-resolution chromatin interactions in Arabidopsis. Mol Cell* ***55****: 694-707.*

*Fransz P, De Jong JH, Lysak M, Castiglione MR, Schubert I. 2002. Interphase chromosomes in Arabidopsis are organized as well defined chromocenters from which euchromatin loops emanate. Proc Natl Acad Sci U S A* ***99****: 14584-14589.*

*Ghavi-Helm Y, Klein FA, Pakozdi T, Ciglar L, Noordermeer D, Huber W, Furlong EE. 2014. Enhancer loops appear stable during development and are associated with paused polymerase. Nature* ***512****: 96-100.*

*Jin F, Li Y, Dixon JR, Selvaraj S, Ye Z, Lee AY, Yen CA, Schmitt AD, Espinoza CA, Ren B. 2013. A high-resolution map of the three-dimensional chromatin interactome in human cells. Nature* ***503****: 290-294.*

*Kim S, Shendure J. 2019. Mechanisms of interplay between transcription factors and the 3D Genome. Mol Cell* ***76****: 306-319.*

*Li G, Ruan X, Auerbach RK, Sandhu KS, Zheng M, Wang P, Poh HM, Goh Y, Lim J, Zhang J et al. 2012. Extensive promoter-centered chromatin interactions provide a topological basis for transcription regulation. Cell* ***148****: 84-98.*

*Liu C, Wang C, Wang G, Becker C, Zaidem M, Weigel D. 2016. Genome-wide analysis of chromatin packing in Arabidopsis thaliana at single-gene resolution. Genome Res* ***26****: 1057-1068.*

*Lupianez DG, Kraft K, Heinrich V, Krawitz P, Brancati F, Klopocki E, Horn D, Kayserili H, Opitz JM, Laxova R et al. 2015. Disruptions of topological chromatin domains cause pathogenic rewiring of gene-enhancer interactions. Cell* ***161****: 1012-1025.*

*Mitchell JA, Fraser P. 2008. Transcription factories are nuclear subcompartments that remain in the absence of transcription. Genes Dev* ***22****: 20-25.*

*Palstra RJ, Simonis M, Klous P, Brasset E, Eijkelkamp B, de Laat W. 2008. Maintenance of long-range DNA interactions after inhibition of ongoing RNA polymerase II transcription. PLoS One* ***3****: e1661.*

*Sakamoto T, Sakamoto Y, Grob S, Slane D, Yamashita T, Ito N, Oko Y, Sugiyama T, Higaki T, Hasezawa S et al. 2022. Two-step regulation of centromere distribution by condensin II and the nuclear envelope proteins. Nat Plants* ***8****: 940-953.*

*Sullivan AM, Arsovski AA, Lempe J, Bubb KL, Weirauch MT, Sabo PJ, Sandstrom R, Thurman RE, Neph S, Reynolds AP et al. 2014. Mapping and dynamics of regulatory DNA and transcription factor networks in A. thaliana. Cell Rep* ***8****: 2015-2030.*

*Therizols P, Illingworth RS, Courilleau C, Boyle S, Wood AJ, Bickmore WA. 2014. Chromatin decondensation is sufficient to alter nuclear organization in embryonic stem cells. Science* ***346****: 1238-1242.*

*van Steensel B, Belmont AS. 2017. Lamina-associated domains: links with chromosome architecture, heterochromatin, and gene repression. Cell* ***169****: 780-791.*

*Wijchers PJ, Krijger PHL, Geeven G, Zhu Y, Denker A, Verstegen M, Valdes-Quezada C, Vermeulen C, Janssen M, Teunissen H et al. 2016. Cause and consequence of tethering a SubTAD to different nuclear compartments. Mol Cell* ***61****: 461-473.*

*Xiao J, Lee US, Wagner D. 2016. Tug of war: adding and removing histone lysine methylation in Arabidopsis. Curr Opin Plant Biol* ***34****: 41-53.*

*Zhao L, Wang S, Cao Z, Ouyang W, Zhang Q, Xie L, Zheng R, Guo M, Ma M, Hu Z et al. 2019. Chromatin loops associated with active genes and heterochromatin shape rice genome architecture for transcriptional regulation. Nat Commun* ***10****: 3640.*

*Zhao L, Zhou Q, He L, Deng L, Lozano-Duran R, Li G, Zhu JK. 2022. DNA methylation underpins the epigenomic landscape regulating genome transcription in Arabidopsis. Genome Biol* ***23****: 197.*

**Second round of review**

**Reviewer 1**

In the revised version submitted by Deng & Zhou et al., the authors added ChIA-PET and RNA-seq data from myc2 mutant and further discussed the function of TF and chromatin configuration regulating gene expression. The FISH results and the published Hi-C data were helpful to prove ChIA-PET availability. ATAC-seq data replaced the DNase-seq and made the data more comparable. In the point-to-point response, the author resolved the majority of the concerns. However, I still have some confusion about the response and the added myc2 part.

1. For Figure 6D, in the first box, the Log2 fold change of the looping genes which lost loops in myc2 was higher than 0, even 1. How to explain the function of these loops in expression regulation? Did that mean the loops indirectly regulated by MYC2 repress gene expression?

2. In response 1.4, the authors mentioned “the expression levels of RNAPII-associated paired anchor genes were highly correlated”, and “RNAPII-, H3K4me3-, and H3K4me1-associated chromatin interactions shared the most anchor”. But in response 1.9, they think “only associated with flowering time genes exhibit higher coexpression than those associated with all interacting genes or non-flowering time genes”. I’m not sure what is the real relation between the same modification and the co-expression. Or do “correlated expression” and “co-expression” mean different?

3. In line 59-60, the authors claimed that TAD-like structures were not observed in Arabidopsis which is inaccurate. The presence of TAD-like structures has been reported in many papers since 2015 (Wang et al., 2015), especially the H3K27me3-related TAD-like structures (Feng et al., 2014; Nutzman et al., 2020; Huang et al., 2021; Yin et al., 2023).

**Reviewer 2**

Most of my concerns have been properly addressed with new data or explaination. Especially the ChIA-PET and RNAseq in myc2 mutant clearly indicates the importance of MYC2 in regulation of the target gene-associated chromatin hubs. As well, the authors used ATAC-seq data from the aerial part of two-weed-old seedings instead of DNase-seq data from 7-day-old seedlings, which is highly commendable. Although the conclusions are similar, it is more reasonable to use data obtained from the same tissues and the same developmental stages.

I have a minor comment on the original concern #4 I have raised before: I agreed that the CRISPR is time-consuming and the authors do not want to perform that in this paper. But I do not believe that the authors claim that genetic editing has not been used to verify the function of chromatin interaction models so far. In fact, there have been reports of using CRISPR technology to edit SNPs in intergenic regulatory elements associated with human diseases to test the function of these intergenic regulatory elements. Take the following two articles for example.

Guo Y, Perez A A, Hazelett D J, et al. CRISPR-mediated deletion of prostate cancer risk-associated CTCF loop anchors identifies repressive chromatin loops[J]. Genome biology, 2018, 19: 1-17.

Luo Z, Rhie S K, Lay F D, et al. A prostate cancer risk element functions as a repressive loop that regulates HOXA13[J]. Cell reports, 2017, 21(6): 1411-1417.

Further, direct manipulation of the chromatin loop has also been reported.

Hao N, Shearwin K E, Dodd I B. Programmable DNA looping using engineered bivalent dCas9 complexes[J]. Nature Communications, 2017, 8(1): 1628.

**Authors Response**

**Point-by-point responses to the reviewers’ comments:**

**Second round of review**

Reviewer #1:

In the revised version submitted by Deng & Zhou et al., the authors added ChIA-PET and RNA-seq data from *myc2* mutant and further discussed the function of TF and chromatin configuration regulating gene expression. The FISH results and the published Hi-C data were helpful to prove ChIA-PET availability. ATAC-seq data replaced the DNase-seq and made the data more comparable. In the point-to-point response, the author resolved the majority of the concerns. However, I still have some confusion about the response and the added *myc2* part.

1. For Figure 6D, in the first box, the Log2 fold change of the looping genes which lost loops in *myc2* was higher than 0, even 1. How to explain the function of these loops in expression regulation? Did that mean the loops indirectly regulated by MYC2 repress gene expression?

***Response 1.1:*** *We appreciate your attention to detail and insightful question regarding the Log2 fold change of looping genes associated with lost loops in myc2. In this study, we calculated the expression changes of looping genes associated with lost loops in myc2 mutant compared to the wild-type. As you noted, we observed an overall upregulation in the median fold change of the anchor genes without MYC2 occupancy in myc2 compared to Col-0. We agree with your suggestion that these MYC2-indirect chromatin interactions tend to be associated with suppressed expression of looping genes.*

*It is important to note that the transcriptional regulation is highly complex, and the effects observed in the absence of MYC2 may involving in intricate regulatory mechanisms. The loss of MYC2 activity may lead to alterations in modulating long-range chromatin interactions and gene expression via other associated transcription factors and regulatory elements in a direct or indirect manner. Therefore, we believe that these indirect regulatory interactions through long-range chromatin interactions may serve multifaceted functions within the regulatory network. Further research is needed to fully understand the underlying mechanisms involved.*

2. In response 1.4, the authors mentioned “the expression levels of RNAPII-associated paired anchor genes were highly correlated”, and “RNAPII-, H3K4me3-, and H3K4me1-associated chromatin interactions shared the most anchor”. But in response 1.9, they think “only associated with flowering time genes exhibit higher coexpression than those associated with all interacting genes or non-flowering time genes”. I’m not sure what is the real relation between the same modification and the co-expression. Or do “correlated expression” and “co-expression” mean different?

***Response 1.2:*** *We thank the reviewer for the comments. In this study, we observed that transcriptionally active marks (H3K4me3, H3K4me1 and RNAPII)-associated chromatin interactions shared the most anchors. We found that the expression levels of active mark-associated paired anchor genes were simultaneously highly expressed and active interacting gene pairs tended to be transcribed cooperatively or co-expression.*

*We apologize for the misunderstanding caused by the statement of "only associated with flowering time genes exhibit higher co-expression than those associated with all interacting genes or non-flowering time genes". Our intention was to highlight that among the active mark-associated interacting genes, those involved in flowering time control exhibited higher co-expression than all interacting genes or those non-flowering time genes. Our results imply that genes controlling flowering time are more likely to be organized together in some specific chromatin connectivity subnetworks for coordinated transcription. We hope this clarification aligns with our previous response have addressed the concerns you raised.*

3. In line 59-60, the authors claimed that TAD-like structures were not observed in Arabidopsis which is inaccurate. The presence of TAD-like structures has been reported in many papers since 2015 (Wang et al., 2015), especially the H3K27me3-related TAD-like structures (Feng et al., 2014; Nutzman et al., 2020; Huang et al., 2021; Yin et al., 2023).

***Response 1.3:*** *Thank you for the comment. We have revised the sentence in the revised version of manuscript as follows:* *TAD-like structures had been identified in a broad range of plant species [22,24,26-29,32-36]. In Arabidopsis, the TAD-like structures were proposed to correspond to heterochromatic compartments and Polycomb repressive histone modifications-associated interacting domains, suggesting that they should rather be referred to as compacted chromatin domains, as they are not functionally equivalent to TADs in animals [23,25,36-38].*

***References***

*22.	Jia J, Xie Y, Cheng J, Kong C, Wang M, Gao L, Zhao F, Guo J, Wang K, Li G, et al. Homology-mediated inter-chromosomal interactions in hexaploid wheat lead to specific subgenome territories following polyploidization and introgression. Genome Biol 2021,* ***22****:26.*

*23.	Grob S, Schmid MW, Grossniklaus U. Hi-C analysis in Arabidopsis identifies the KNOT, a structure with similarities to the flamenco locus of Drosophila. Mol Cell 2014,* ***55****:678–693.*

*24.	Dong Q, Li N, Li X, Yuan Z, Xie D, Wang X, Li J, Yu Y, Wang J, Ding B, et al. Genome-wide Hi-C analysis reveals extensive hierarchical chromatin interactions in rice. Plant J 2018,* ***94****:1141–1156.*

*25.	Feng S, Cokus SJ, Schubert V, Zhai J, Pellegrini M, Jacobsen SE. Genome-wide Hi-C analyses in wild-type and mutants reveal high-resolution chromatin interactions in Arabidopsis. Mol Cell 2014,* ***55****:694–707.*

*26.	Liu C, Cheng YJ, Wang JW, Weigel D. Prominent topologically associated domains differentiate global chromatin packing in rice from Arabidopsis. Nat Plants 2017,* ***3****:742–748.*

*27.	Dong P, Tu X, Chu PY, Lu P, Zhu N, Grierson D, Du B, Li P, Zhong S. 3D chromatin architecture of large plant genomes determined by local A/B compartments. Mol Plant 2017,* ***10****:1497–1509.*

*28.	Wang M, Wang P, Lin M, Ye Z, Li G, Tu L, Shen C, Li J, Yang Q, Zhang X. Evolutionary dynamics of 3D genome architecture following polyploidization in cotton. Nat Plants 2018,* ***4****:90–97.*

*29.	Dong P, Tu X, Li H, Zhang J, Grierson D, Li P, Zhong S. Tissue-specific Hi-C analyses of rice, foxtail millet and maize suggest non-canonical function of plant chromatin domains. J Integr Plant Biol 2020,* ***62****:201–217.*

*32.	Xie T, Zhang FG, Zhang HY, Wang XT, Hu JH, Wu XM. Biased gene retention during diploidization in Brassica linked to three-dimensional genome organization. Nat Plants 2019,* ***5****:822–832.*

*33.	Pei L, Huang X, Liu Z, Tian X, You J, Li J, Fang DD, Lindsey K, Zhu L, Zhang X, et al. Dynamic 3D genome architecture of cotton fiber reveals subgenome-coordinated chromatin topology for 4-staged single-cell differentiation. Genome Biol 2022,* ***23****:45.*

*34.	Wang L, Jia G, Jiang X, Cao S, Chen ZJ, Song Q. Altered chromatin architecture and gene expression during polyploidization and domestication of soybean. Plant Cell 2021,* ***33****:1430–1446.*

*35.	Concia L, Veluchamy A, Ramirez-Prado JS, Martin-Ramirez A, Huang Y, Perez M, Domenichini S, Rodriguez Granados NY, Kim S, Blein T, et al. Wheat chromatin architecture is organized in genome territories and transcription factories. Genome Biol 2020,* ***21****:104.*

*36.	Yin X, Romero-Campero FJ, Yang M, Baile F, Cao Y, Shu J, Luo L, Wang D, Sun S, Yan P, et al. Binding by the Polycomb complex component BMI1 and H2A monoubiquitination shape local and long-range interactions in the Arabidopsis genome. Plant Cell 2023,* ***35****:2484–2503.*

*37. Dong P, Tu X, Liang Z, Kang BH, Zhong S. Plant and animal chromatin three-dimensional organization: similar structures but different functions. J Exp Bot 2020,* ***71****:5119–5128.*

*38. Huang Y, Sicar S, Ramirez-Prado JS, Manza-Mianza D, Antunez-Sanchez J, Brik-Chaouche R, Rodriguez-Granados NY, An J, Bergounioux C, Mahfouz MM, et al. Polycomb-dependent differential chromatin compartmentalization determines gene coregulation in Arabidopsis. Genome Res 2021,* ***31****:1230–1244.*

Reviewer #2:

Most of my concerns have been properly addressed with new data or explaination. Especially the ChIA-PET and RNAseq in *myc2* mutant clearly indicates the importance of MYC2 in regulation of the target gene-associated chromatin hubs. As well, the authors used ATAC-seq data from the aerial part of two-weed-old seedings instead of DNase-seq data from 7-day-old seedlings, which is highly commendable. Although the conclusions are similar, it is more reasonable to use data obtained from the same tissues and the same developmental stages.

I have a minor comment on the original concern #4 I have raised before: I agreed that the CRISPR is time-consuming and the authors do not want to perform that in this paper. But I do not believe that the authors claim that genetic editing has not been used to verify the function of chromatin interaction models so far. In fact, there have been reports of using CRISPR technology to edit SNPs in intergenic regulatory elements associated with human diseases to test the function of these intergenic regulatory elements. Take the following two articles for example.

Guo Y, Perez A A, Hazelett D J, et al. CRISPR-mediated deletion of prostate cancer risk-associated CTCF loop anchors identifies repressive chromatin loops[J]. Genome biology, 2018, 19: 1-17.

Luo Z, Rhie S K, Lay F D, et al. A prostate cancer risk element functions as a repressive loop that regulates HOXA13[J]. Cell reports, 2017, 21(6): 1411-1417.

Further, direct manipulation of the chromatin loop has also been reported.

Hao N, Shearwin K E, Dodd I B. Programmable DNA looping using engineered bivalent dCas9 complexes[J]. Nature Communications, 2017, 8(1): 1628.

***Response:*** *We agree with the reviewer’s comments regarding the utilization of CRISPR technology to edit SNPs in intergenic regulatory elements and assess their association with disease susceptibility through chromatin loop/chromosome configuration**. Furthermore, the manipulation of chromatin loop formation using engineered bivalent dCas9 complexes is also feasible. Our intention was to highlight the scarcity of* *studies focused on validating the functional chromatin interaction model through genetic editing in higher plants, particularly in relation to the flowering time-associated SNP–chromatin loop–flowering time gene model (as list in Additional file 7: Table S6). We apologize for any confusion caused by the unclear definition in the Point-by-point responses to the reviewers’ comments (Response 2.4).*
